# Co-optimized volumetric muscle design for large dynamic deformations in versatile biohybrid robots

**DOI:** 10.1038/s41467-026-77655-1

**Published:** 2026-09-11

**Authors:** Aiste Balciunaite, Mike Y. Michelis, Miriam Filippi, Pablo Paniagua, Oncay Yasa, Robert K. Katzschmann

**Affiliations:** 1 Soft Robotics Laboratory, ETH Zurich, Zürich, Switzerland; 2 ETH AI Center, ETH Zurich, Zürich, Switzerland; 3 Thrust of Robotics and Autonomous Systems and Thrust of Bioscience and Biomedical Engineering, The Hong Kong University of Science and Technology (Guangzhou), Guangzhou, China

**Keywords:** Biomaterials - cells, Computational models, Musculoskeletal models

## Abstract

Biohybrid robots use engineered living muscle tissue as actuators, offering self-organization, adaptability, and self-healing. Current designs focus on improving muscle physiology but overlook the interplay between muscle and scaffold in the robot’s function. We present a computational and experimental pipeline to co-optimize the form and function of centimeter-scale bioactuators. Using a soft-body simulation framework, we modeled unified muscle–scaffold systems and applied an evolutionary algorithm and targeted parameter sweeps to maximize range of motion. The resulting bioactuators, fabricated by integrating skeletal muscle tissue with microgrooved hydrogel scaffolds, feature a continuous interface enabling efficient force transmission and large, rapid deformations. They achieved up to an eleven-fold increase in range of motion over previous designs of similar muscle volume, powering robots capable of jumping, swimming, walking, and gripping, and scalable into multi-unit systems. By unifying modeling, optimization, and fabrication, we demonstrate how such a pipeline accelerates the development of high-performance biohybrid robots.

## Abstract (web summary)

The authors combine computer simulation and experimental fabrication to co-design living muscle and its scaffold, optimizing the range of motion of bioactuators to power biohybrid robots that can jump, swim, walk and grip.

## Introduction

Biohybrid robots are an emerging class of soft robots powered by living muscle instead of traditional, non-living actuation mechanisms^1^. Integrating muscle cells with soft artificial materials produces biological actuators (bioactuators) with advanced features such as adaptability and self-healing that are difficult to engineer with conventional abiotic actuators^2,3^. Biohybrid robots can take a variety of forms, such as swimming biohybrids in the shape of fish^4^, rays^5–7^, or jellyfish^8^ fabricated from single-cell layers ( < 10 µm thickness) of cardiac cells combined with soft scaffold materials to propel themselves through fluid using beating motions. Other designs use ring- or block-shaped skeletal muscle tissues (5–16 mm in length, 100–500 µm thickness) coupled to artificial skeletons to crawl^9–13^, swim^14^, or achieve pick-and-place tasks in an antagonistic arrangement that mimics musculoskeletal anatomy^15^.

Recent work has focused on increasing the functionality of biohybrid robots by improving the biological state of the tissue engineered muscle^13,16^ or using an arrangement of multiple bioactuators^17,18^. But in natural systems, muscle architecture and geometry are also tuned to muscle function; these design dimensions remain underexplored in biohybrid systems. To further push the boundaries of biohybrid robotics, we must start to consider the effects of all elements of the bioactuator together: the living biological muscle and non-living materials that serve as a scaffold to guide muscle growth and a skeleton for the muscle to act against. Expanding the design space to include diverse muscle geometries and scaffold properties demands computational modeling, as biofabrication itself is costly and time-consuming. While the biomechanics community has developed methods to model the interaction between different body parts in a soft deformable matter, biohybrid robotics still lacks modeling frameworks that can capture the mechanics and actuation behavior at the appropriate complexity, resolution, and speed for optimization^19–22^.

Models developed for volumetric biohybrid muscle actuators must enable a bottom-up combination of constitutive materials to successfully probe the entire parameter space. A basic approach represents bioactuators as serially connected rod-like structures where actuation is modeled as a traveling wave generating force along the fiber-like structure^10^. However, this method neglects the role of the surrounding matrix in dissipating energy during contraction and cannot be used for arbitrary muscle geometries, while a biological muscle can maintain its contractive ability in a variety of shapes. Soft body modeling frameworks with voxel-based representations can be used instead to account for material properties and more complex muscle shapes^23^. The voxels contract with spring-like mechanics, defined by a rest length of compression. The voxel-based approach was used for morphological optimization within these actuated biohybrid soft robots^24^. Due to the choice of double Euler integration, the number of timesteps can become prohibitively large to maintain the stability of the simulation. Moreover, the use of Euler-Bernoulli beams to connect the voxels assumes a linear beam theory, reducing the accuracy at larger deformations. A further iteration of the voxel-based approach, used in this work, uses a spring-like contraction model with irregular meshes in a custom, open-source finite element simulation. We incorporate stable neo-Hookean constitutive laws that enable accurate large deformations^25^, in addition to implicit Euler time integration that guarantees numerical stability and allows larger timesteps for computational efficiency.

Optimizing biohybrid robot designs requires search methods to explore the large configuration space. Evolutionary algorithms are well-suited to the task due to the lack of first-order gradient information in the simulation models, but they require rapid simulations. To date, only two studies have demonstrated optimization of biohybrid designs. Kriegman et al. used a voxel-based approach to optimize microscale reconfigurable organisms ( < 1 mm in all dimensions)^24^. Zimmerman et al. showed a purely geometric, biologically agnostic model to optimize contractile thin films in fluid that did not consider the shape or properties of the muscle layer^26^. The contractile films were only a few millimeters in the longest dimension, and the active layer was a thin, single-cell layer of approximately 10 µm. Therefore, the optimization of centimeter-scale biohybrid actuators accounting for volumetric muscle and scaffold material behavior has not yet been achieved.

In this work, we present a pipeline to co-optimize the volumetric muscle and scaffold elements of a biohybrid muscle-driven robot to achieve centimeter-scale bioactuators optimized for dynamic movements at 1 to 3 Hz frequencies with a wide range of motion up to 6.6 mm. We modeled bioactuators using a soft-body simulation framework and optimized the muscle and scaffold geometry for rapid large deformations. Then, we established a fabrication method for the computationally optimized bioactuators by stably integrating a muscle tissue layer with a soft hydrogel scaffold, enabling continuous force transmission. Our pipeline resulted in bioactuators with up to eleven times larger ranges of motion than previous versions of comparable muscle volume. The versatility of our bioactuator design is highlighted by showcasing biohybrid robots that could jump, swim, walk, and grip. Finally, we extended our pipeline to more complex geometries by making bioactuators consisting of multiple muscle units.

## Results

**Fig. 1:**
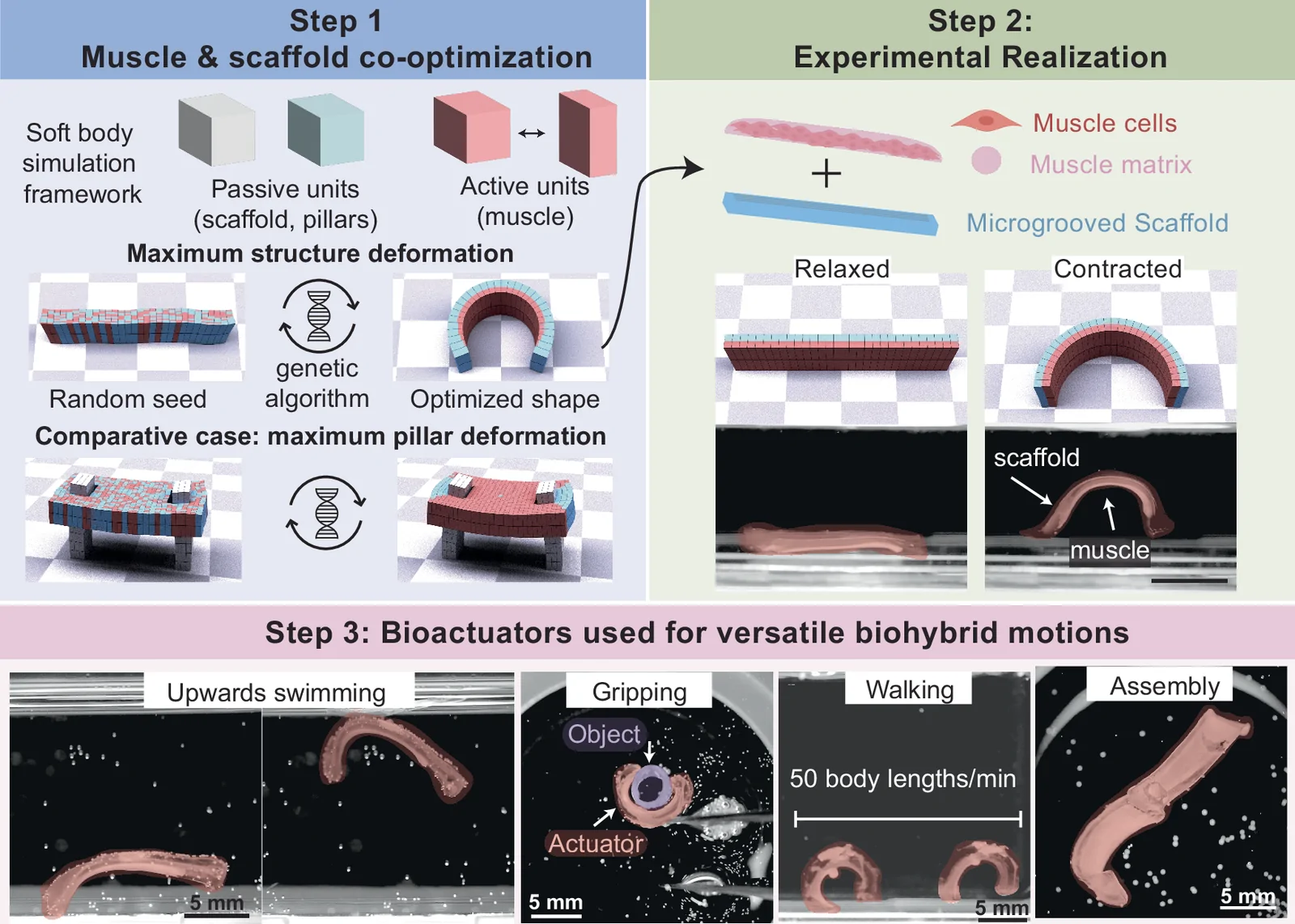
Computationally guided experimental pipeline for bioactuators with large dynamic deformations. In step 1, we designed biohybrid systems through volumetric co-optimization of muscle and scaffold elements using a soft-body simulation framework combined with an evolutionary algorithm. We evolved actuator geometries to maximize the range of motion, finding a volumetric bilayered architecture. As a comparative case, we showed that our algorithm can also be used for optimizing pillar deformation with volumetric muscle. In step 2, we fabricated volumetric bilayers. Finally, we experimentally demonstrated the advantages of the optimized bioactuators through diverse locomotion and manipulation tasks, including swimming and gripping, and further illustrated scalability through modular assembly.

We designed biohybrid systems through volumetric co-optimization of muscle and scaffold elements using a soft body simulation framework combined with an evolutionary algorithm (Fig. 1). We evolved actuator geometries to maximize range of motion, finding a volumetric bilayered architecture. As a comparative case, we showed that our algorithm can be used for optimizing pillar deformation with volumetric muscle, a common biohybrid geometry. In step 2, we fabricated volumetric bilayers by stably integrating a volumetric muscle layer into a hydrogel scaffold. Finally, we experimentally demonstrated the advantages of the optimized bioactuators through diverse locomotion and manipulation tasks and further illustrated scalability through modular assembly.

### Co-optimization of volumetric muscle and scaffold elements of biohybrid actuators

**Fig. 2:**
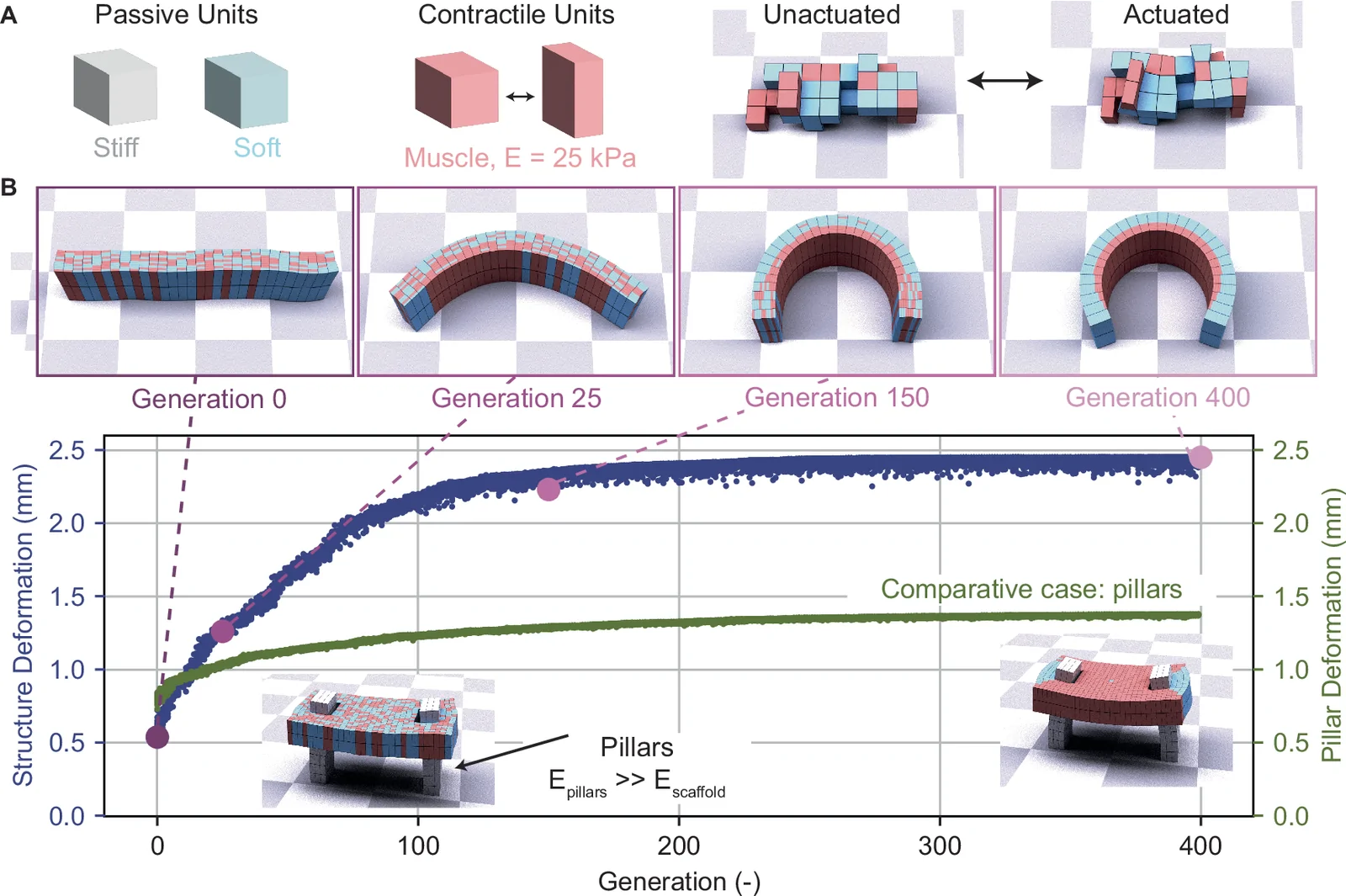
Computational co-optimization of the volumetric muscle and scaffold elements of biohybrid actuators. **A** We defined biohybrid actuators in simulation as objects consisting of contractile and non-contractile materials. **B** We used an evolutionary algorithm to optimize for a geometry that generated the maximum range of movement (structure deformation). Examples of the actuator at generation 0, 25, 150, and 400 are shown above the plot. As a comparative case, we used our method to optimize the bioactuator geometry for maximum deformation of pillars (y-axis on the right), a common biohybrid setup. The pillars were modeled as a stiff, passive material. Insets in the graph show the original randomized design and the final optimized bioactuator design.

To identify bioactuator geometries that can achieve a desired performance, we present a computational algorithm based on a soft body simulation framework. Previous biohybrid computational models have made assumptions to simplify the contraction modeling that ignore the soft material aspects of a muscle for computational efficiency. While such models can be useful in specific cases, a biohybrid muscle is fundamentally a contractile soft material, and therefore a model applicable to centimeter-scale tissues must capture the soft-body dynamics in the muscle material. We developed a soft-body simulation framework to capture the material dynamics of the moving bioactuator^27^. The simulated bioactuator consisted of contractile (muscle tissue) and non-contractile (hydrogel scaffold) units with the nonlinear material properties based on experimental data (Fig. 2A and Fig. S1). The muscle contraction was modeled as a uniaxial, spring-like contraction behavior of desired voxels, recapitulating the elastic elements of biological muscle^28^. The voxel-based modeling approach enabled geometric freedom and unrestricted material selection, allowing for a variety of bioactuator designs to be explored within the simulation framework, as well as modeling aspects such as empty space between muscle and scaffold.

An individual simulation took less than 5 s, which allowed us to couple the bioactuator model with a parallelized evolutionary algorithm to optimize a generic rectangular bioactuator for deformation (Fig. 2B). We used the Non-dominated Sorting Genetic Algorithm (NSGA-II) from the Parallel Global Multiobjective Optimizer (PAGMO) package^29^ to search a binary voxel grid determining whether a finite element, a hexahedron in this case, is an actively actuated muscle or not. The genetic algorithm uses a population size to run multiple simulations in parallel; we fixed this value at 20. We encouraged a broader, more random search space by changing the default setting of the distribution index for mutation from 50 to 20. This can be seen in Fig. 2B as the different samples in a population have a wide spread, whereas the default mutation distribution index setting would result in a too exploitative search that might converge to a local optimum. We fixed the search space to a cuboid domain, since our additional experiments (Fig. S2) showed a convergence towards as-thin-as-possible two-layered structures if material is allowed to be removed, and we would like to further understand the muscle allocation within such thin bioactuators.

We randomly seeded an initial generation and continuously permuted the assembly of contractile and scaffold elements selected for maximum deformation. To quantify the deformation, we observed the contraction after 0.25 s of simulation time and then waited until it had fully stopped oscillating after a total of 0.4 s. The maximal deformation was calculated as the maximum distance between the initial and the final mesh vertex positions. The final algorithm converged on a bilayered geometry with an inner muscle layer that decreases in size towards the edges and an outer scaffold layer that resulted in a bending motion upon contraction of the inner active layer.

As a comparative case, we demonstrated the applicability of our algorithm to common biohybrid set-ups like a bioactuator around two pillars, maximizing for pillar deformation (Fig. 2B). In this case, our algorithm converged to a muscle and scaffold distribution where passive elements were on the outside of the pillars, while the remainder of the structure was muscular.

**Fig. 3:**
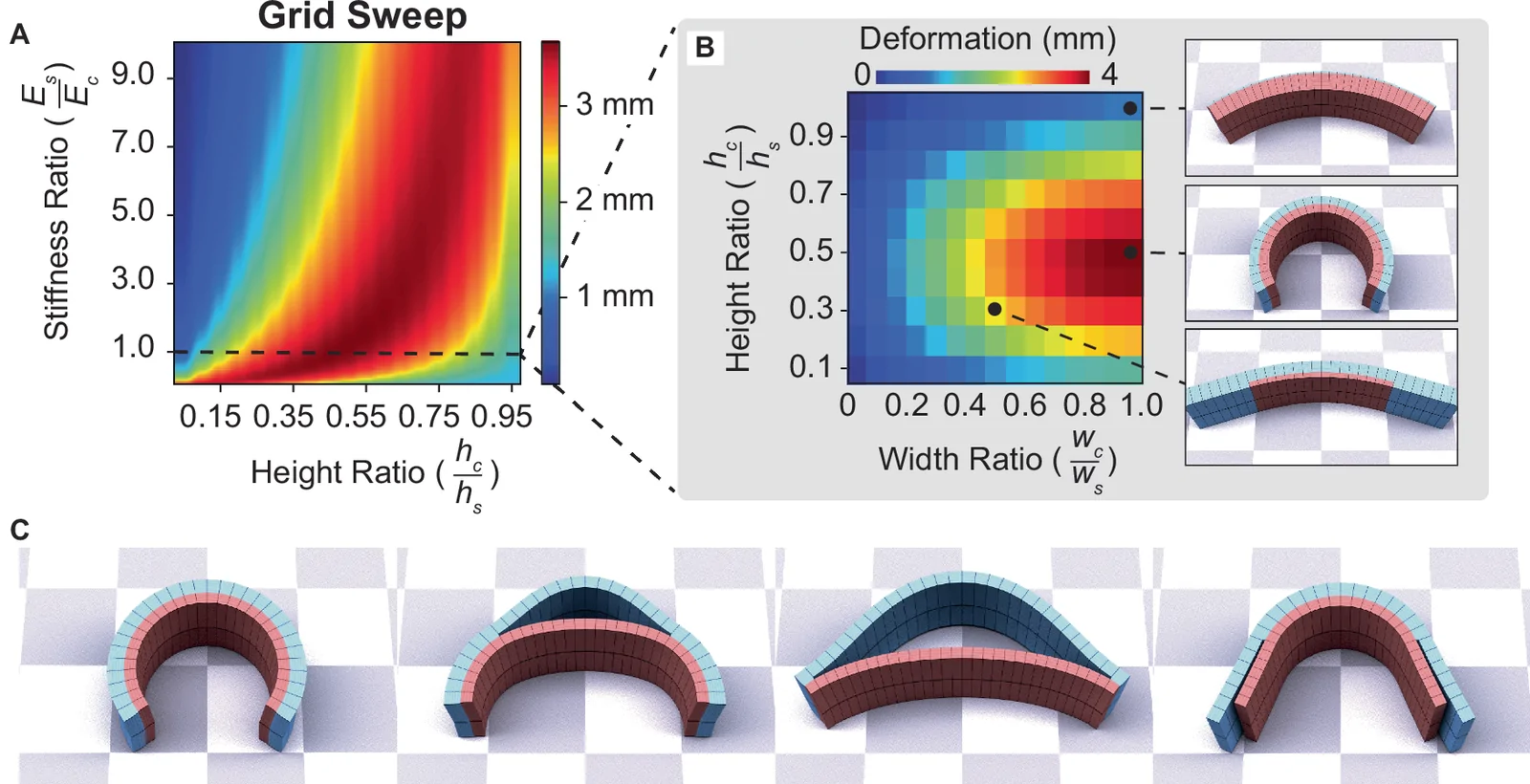
Computational analysis on designing volumetric bilayer bioactuators. **A** Assuming a bilayered design, the distribution of muscle and scaffold of the bioactuator was refined by a grid optimization study to look at the effect of scaffold stiffness ratios and height ratios on deformation. **B** For a fixed ratio of 1 between the scaffold and contractile unit stiffnesses, the optimal bioactuator geometry was where the contractile layer comprises 40% of the height and spans 100% of the length. **C** Various structures where the muscle detaches from the scaffold. From left to right: A fully connected bilayer deforming 2.44 mm, approximately 50% of the muscle is detached in the middle deforming 1.70 mm, only the two ends are attached between muscle and scaffold deforming 1.46 mm, and lastly roughly half the muscle is detached on the outer edges deforming 2.16 mm.

We continued on this finding that a bilayered design maximizes the range of motion, simplified the design with a constant-profile bilayer for easier analysis of the bioactuator, and further investigated specific distributions of the muscle and scaffold components. We used our bioactuator model to run a grid sweep on the ratio of scaffold to muscle stiffness and the ratio of the height of the muscle and scaffold layers (Fig. 3A). The contraction force was applied as a uniaxial compressive stress in each hexahedral contractile unit and kept constant throughout the sweeps. Optimal deformation of the bioactuators was dependent on both muscle volume and material stiffness. As the stiffness of the scaffold increased, a greater height of muscle was necessary for maximum deformation upon contraction. For soft scaffolds, small muscle heights were optimal because when the muscle height was larger, the movement profile was a linear contraction rather than bending. However, stiffer scaffolds also resulted in less motion, despite increasing muscle volume that would provide more contraction force.

As our aim was to model bioactuators, we informed our computational investigation by preliminary experiments with bioactuators of different stiffnesses and found that a muscle to scaffold stiffness ratio of 1 was appropriate for the interaction of the scaffold with the muscle layer (Fig. S1). Therefore, we conducted a more detailed grid sweep of muscle and scaffold geometries for a fixed stiffness ratio of 1. We found that a bioactuator where the active layer comprises 40–50% of the height and spans 100% of the length maximizes deformation upon contraction (Fig. 3B). This optimal active layer height heavily depends on the stiffness ratio between both materials and has to be taken into account during bioactuator fabrication and choice of muscle and scaffold material.

Lastly, we investigated the influence of adhesion between muscle and scaffold (Fig. 3C). We modeled removing connective tissue between the two layers by using empty voxels between muscle and scaffold layers to model a separated tissue and scaffold. We found that full adhesion results in the largest range of motion, where especially the central area matters and the muscle’s outer edges have a smaller impact on the final deformation.

### Optimizing volumetric bilayer fabrication for biohybrid interface stability

Based on our bioactuator motion optimization algorithm, we aimed to produce volumetric bilayer biohybrid actuators on a hydrogel scaffold of 15 × 3.5 × 1 mm. In our modeling results, we found that the muscle tissue should cover the surface area of the scaffold and comprise approximately 40–50% of the thickness of the entire bioactuator for optimal deformation. Single-cell layers of muscle cells would not be sufficient; we needed to grow a tissue on the scaffold. Further, the modeling demonstrated the importance of perfect adhesion between the muscle and scaffold layers for efficient force transfer; to achieve the same results experimentally, our fabrication method needed to stably integrate a muscle layer onto the soft hydrogel. Finally, the fabrication method needed to be robust enough to differentiate functional, contractile skeletal muscle tissue in a repeatable fashion.

**Fig. 4:**
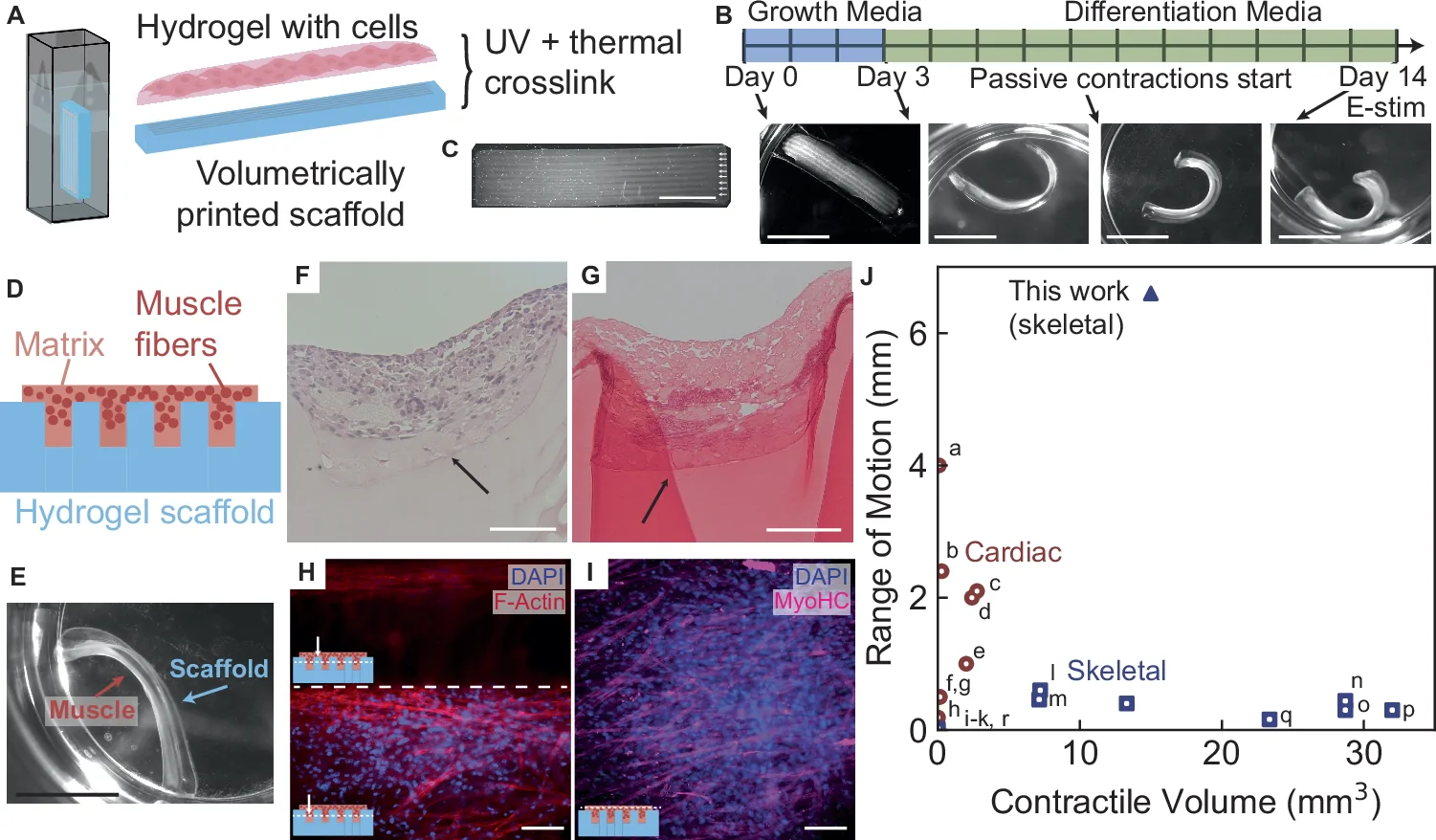
Volumetric bilayer bioactuator fabrication and characterization. **A** Bioactuators (15 mm length, 3.5 mm width, 1.5 mm height) were prepared by post-seeding a hydrogel containing C2C12 mouse myoblasts to a hydrogel scaffold and cross-linking with UV and temperature. **B** After fabrication, bioactuators were grown and differentiated using a two-week protocol. **C** Image of the hydrogel scaffold prior to seeding, with arrows indicating the grooves. **D** Sketch of the cross-section of muscle and scaffold layers. Groove dimensions are 300 µm in width, 500 µm in depth, and 12 mm in length. **E** Labeled stereomicroscope image of the bioactuator (bottom). **F** Histological cross-section stained with H&E of a groove, showing the distribution of cells in the muscle layer (purple nuclei), corresponding to the view in (**D**). The scaffold-muscle interface is indicated with an arrow. **G** Histological cross-section stained with picrosirius red showing a groove, corresponding to the view in (**D**). **H** Confocal microscopy image of the lower part of the muscle layer, showing myofibers stained with F-actin (red) inside of a groove. Dashed line shows the border of a microgroove. Cell nuclei are shown in blue. **I** Confocal microscopy image of upper muscle layer of bioactuator, with mature myofibers stained with MyoHC (purple) and nuclei stained in blue (DAPI). **J** Comparison of the maximum range of motion for bioactuators of different contractile volumes. See Table S1 for values and references of previously reported biohybrid robots. Scale bars in (**B**), (**C**), and (**G**) are 1 mm, scale bars in (**E**), (**F**), (**H**), (**I**) are 100 µm.

Building on the concept of a volumetric bilayer bioactuator first introduced in Balciunaite et al.^30^, microgrooved hydrogel scaffolds were prepared by xolographic printing^31^, a linear volumetric printing technique recently adapted for use with hydrogels^32–34^. Advantageously, the use of xolography allowed quick prototyping of scaffold geometries with microscale resolution, as each scaffold took approximately 60 s of printing, and the fabrication method was robust to changes in geometry. A skeletal muscle tissue layer of dimensions 12 × 3 × 0.3 mm was formed on top of the scaffold by depositing a mixture of 5 wt% GelMA, 0.1 wt% LAP, and 66% Matrigel with 5 × 10^6^ C2C12 cells onto the scaffold and then following a growth and differentiation protocol to form contractile biohybrid muscle (Fig. 4A, B). We use the term “skeletal muscle tissue layer” throughout this manuscript to refer to the engineered, contractile layer of the bioactuator; while this layer is consistent with 3D biohybrid muscle tissues, it is not fully representative of a physiological tissue, which would require multiple cell types, perfusability, and other features. The fabrication method was optimized by changing the cell-containing hydrogel material to contain only GelMA and Matrigel and the treatment of the scaffold prior to cell seeding to maximize the yield of functional bioactuators to 85%.

The preparation method of the scaffold prior to the deposition of the cell-containing hydrogel was optimized to maximize adhesion stability between the two layers. If the adhesion was insufficient, the muscle tissue layer would float off the scaffold within 48 h of seeding (Fig. S3). First, we found that the presence of microgrooves on the scaffold aided stable adhesion (Fig. 4C, D). The tissue layer on scaffolds without microgrooves did not integrate over the length of the scaffold and instead peeled off from the ends (Fig. S4). Further, a drying step before applying the cell-laden hydrogel to the 3D-printed hydrogel scaffold was crucial for stable adhesion, to avoid the presence of a water layer that impeded the interaction of the two materials. Lack of post-crosslinking with UV resulted in reduced adhesion of the tissue hydrogel to the scaffold, though initial adhesion was still observed (Fig. S5). We assessed the biocompatibility of the hydrogel preparation process by Live/Dead staining 24 h after cell seeding (Fig. S6). We found the best procedure to be soaking in PBS overnight to equilibrate pH and remove unreacted photoinitiator molecules, then washing in DI water before drying to avoid the formation of a salt layer (Fig. S6).

During differentiation, skeletal muscle cells undergo maturation whereby they remodel their environment and fuse into multinucleated myofibers, causing the tissue to shrink^9^. Engineered skeletal muscle tissue requires stabilizing structures, often pillars, that provide a mechanical force to keep the tissue from shrinking into itself^9,15^. In our system, the hydrogel scaffold acted as a soft exoskeleton that provided the necessary mechanical stability for the skeletal muscle tissue to maintain its shape over the maturation phase (Fig. 4E). Prior to optimization of the fabrication protocol, the unsuccessful adhesion of the tissue and scaffold resulted in tissue shrinking within 48 h of the growth phase (Fig. S7). Therefore, we identified that the biohybrid interface between the scaffold and muscle tissue was critical to the formation of functional bioactuators. To investigate the morphology of the muscle layer and the interface, we stained cross-sections of bioactuators with H&E (Fig. 4F). This revealed that the hydrogel scaffold had no presence of cells, whereas the muscle tissue layer is heterogeneously stained with porous structures and cells marked by dark purple nuclei. Picrosirius Red staining of collagenous connective tissue revealed two distinct regions within the tissue layer: a darker red area below and a section above that is stained lighter and more porous (Fig. 4G). Because the muscle tissue layer at the interface with the scaffold did not contain a large proportion of cells, we concluded that the largest influence on tissue layer adhesion to the scaffold arises from a stable hydrogel-hydrogel adhesion. H&E staining of a coronal section of the scaffold showed a similar morphology to the perpendicular cross-section (Fig. S8). Myofibers, identified by the elongated nuclei in the upper layer of the muscle tissue, were localized towards the top 100 µm of the tissue layer.

The mechanical and topographical features of the hydrogel scaffold also influenced the morphology of the skeletal muscle tissue. The grooves served as topographical cues to promote uniaxial myofiber alignment at the grooves (Fig. 4H, I and S9). Myofibers within the microgrooves were aligned in parallel with the direction of the grooves (Fig. 4H), and the alignment propagated upwards as the muscle tissue layer above the microgrooves was aligned in the direction of the grooves (Fig. 4I). As markers of maturation, we measured an average myofiber thickness of 14.4 ± 5.5 µm and a fusion index of 75 ± 11% (Fig. S10). While the imaging approach was not specifically intended to evaluate sarcomeric organization, features consistent with sarcomeric structure were nevertheless observed (Fig. S11). The specificity of the MyoHC staining was confirmed using undifferentiated C2C12 cells, which lack mature myofibers and showed no MyoHC signal with identical staining antibodies (Fig. S12). An isotype control further confirmed the absence of nonspecific binding attributable to the antibody isotype (Fig. S13). From a mechanical perspective, a scaffold stiffness of 16.5 kPa allowed the bioactuators to slightly curve over the differentiation process, without curling into a helical shape as occurred with softer scaffolds (Fig. S1).

We compared the range of motion achieved in our work with previously reported biohybrid robots to evaluate whether the volumetric bilayered shape effectively improved range of motion, as predicted by the computational modeling pipeline (Fig. 4J). An analysis of biohybrid robots published over the last two decades shows that bioactuators with increased tissue contractile volume exhibit a decreased absolute range of motion. Tissue contractile volume was taken from the text or estimated based on images (Table S1). For purposes of comparison, the largest deformation of the biohybrid actuator was taken to be the largest movement of the actuator; for planar actuators, this would be a bending motion. Importantly, the previously reported bioactuators were not optimized and validated for range of motion by a computational pipeline. Therefore, we highlight that by using our computational pipeline to co-optimize muscle and scaffold geometry and developing a suitable fabrication method that enabled continuous force transmission across the biohybrid muscle interface, our bioactuators demonstrated a substantial increase in range of motion for actuators with similar contractile volumes.

### Bilayer biohybrid robots

Having optimized bioactuators for large deformations, we investigated their performance for biohybrid robots. The application of an electric field in the construct media induced a uniaxial contraction of the muscle layer, which then caused bending of the actuator due to coupling with the non-contractile elastic scaffold. Once the muscle contraction finished, the construct returned to its passive state due to the relaxation of the muscle layer as well as the elastic energy stored in the hydrogel scaffold. We analyzed the performance of five different bioactuators produced using a scaffold size of 15 × 3.5 × 1 mm with 300 µm width microgrooves. The individual contraction behavior of each actuator was consistent, with standard deviations up to 6% when comparing consecutive contractions (Table S2). However, we observed a large inter-actuator variability in range of motion ranging from 0.5 mm of the least-performant bioactuator to 6.6 mm for the most-performant one.

#### Propulsion upwards

**Fig. 5:**
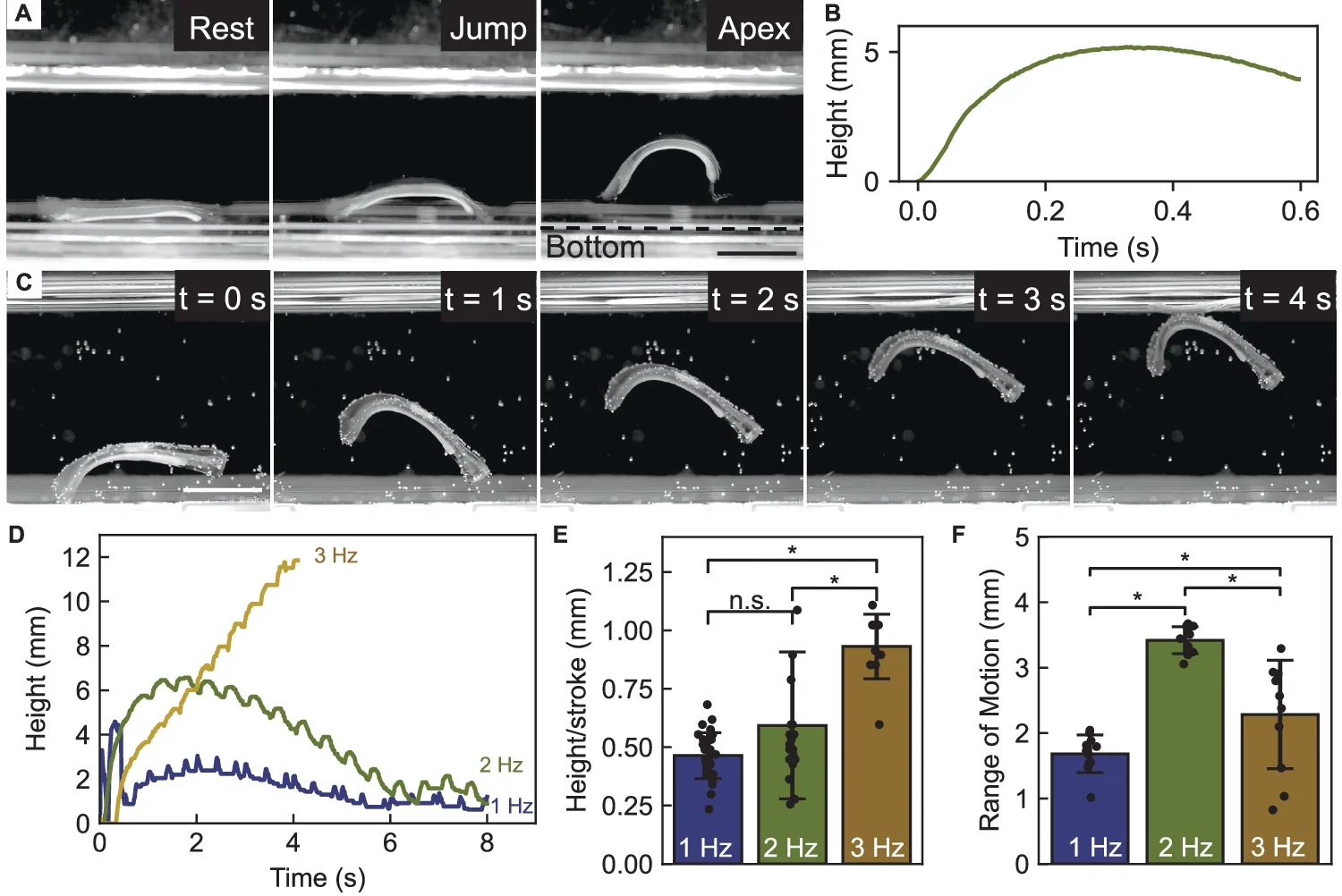
Swimming and jumping biohybrid robots from optimized bioactuators. **A** A time course of a jumping biohybrid robot consisting of a bioactuator that, upon contraction, propelled the robot from the bottom of the dish. **B** Track of the height gain of bioactuator. **C** Bioactuators were tuned to exhibit swimming behavior moving from the bottom of the dish to the air-liquid interface. The swimming behavior was frequency dependent (**D**), due to the different characteristics of **E** height gain per stroke and **F** range of motion at different actuation frequencies. Error bars show standard deviation. All scale bars are 5 mm.

We found that a single stroke movement generated enough thrust to cause a bilayered bioactuator to jump off the bottom of a dish (Fig. 5A). Upon application of an electric stimulus, the actuator curved inwards, with a range of motion of 6.6 mm. The upward motion from a single stroke was 5.1 mm (Fig. 5B), corresponding to 33% of the actuator’s body length. After jumping, the bioactuator returned to the bottom of the dish (Supplementary Movie 1).

We then investigated the ability of our bioactuators to locomote in different buoyancy regimes: at the bottom of the dish (sinking) and in transition (swimming upwards) (Fig. 5C). Without modification, our bioactuators were denser than the surrounding fluid and thus rested on the bottom of the dish. Therefore, we increased the buoyancy of a bioactuator by adding a buoyancy-adjusting material to the hydrogel scaffold after the maturation protocol. At rest, the bioactuator was still denser than the surrounding fluid and thus equilibrated to rest at the bottom of the dish. When stimulated to contract at 1 Hz, the bioactuator first swam upwards but then sank again (Fig. 5D and Supplementary Movie 2). When stimulated at 2 Hz, the movement profile was similar: the bioactuator swam upwards, then sank again (Fig. 5D and Supplementary Movie 3). At 3 Hz actuation frequency, however, the bioactuator swam upwards and reached the air-liquid interface, where it remained (Fig. 5D and Supplementary Movie 4). As expected, the comparison of upward movement per stroke of each actuation at 1 Hz, 2 Hz, and 3 Hz showed that more height is gained per stroke in the 3 Hz case, resulting in the actuator swimming to the surface (Fig. 5E, Fig. S14, and Table S3). However, this is not due to a corresponding change in the amplitude of the bioactuator contraction: the actuator had a smaller contraction amplitude at 1 and 3 Hz frequencies than at 2 Hz (Fig. 5F and Table S4).

#### Interaction with macroscale objects in the environment

**Fig. 6:**
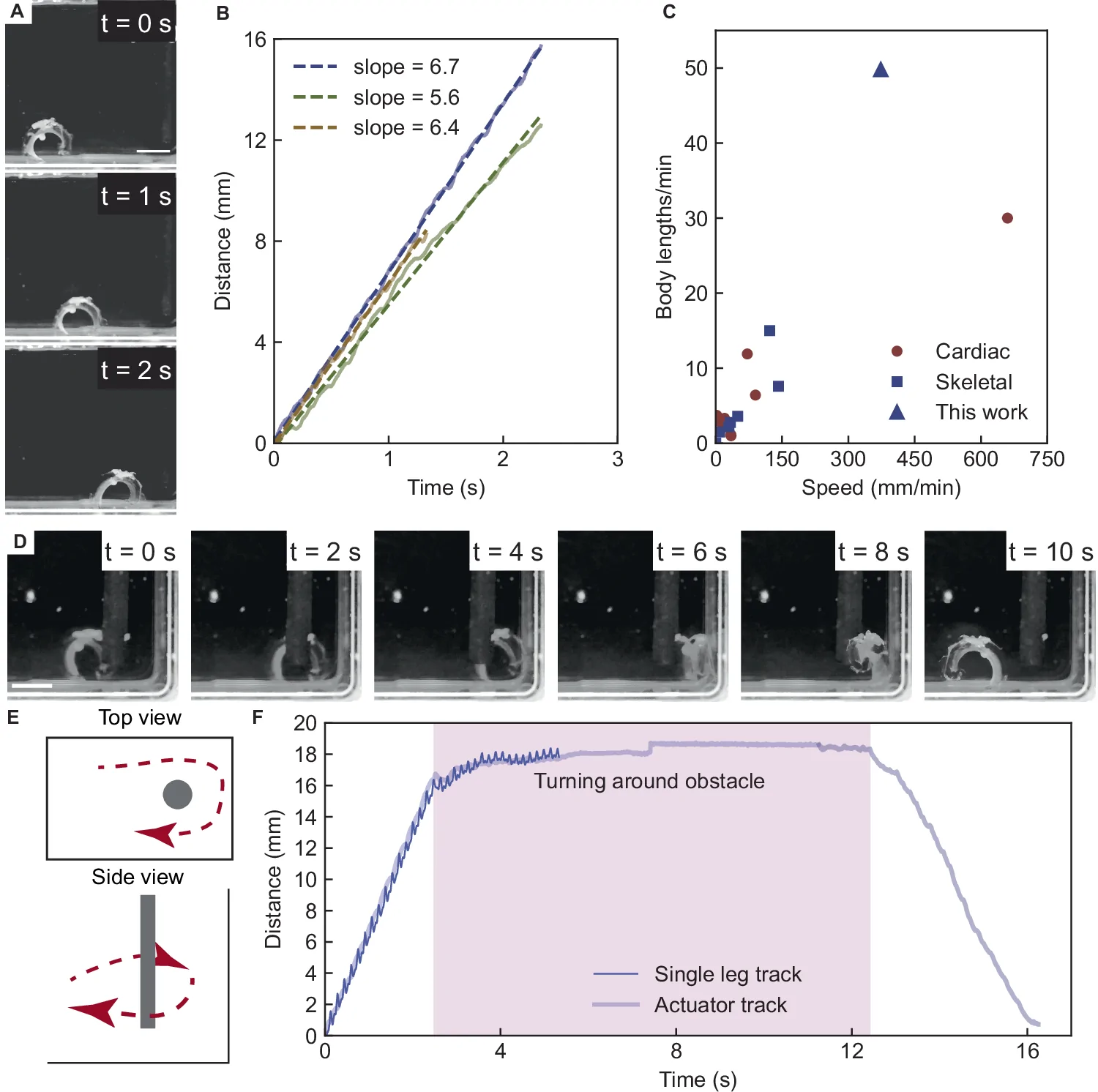
Walking biohybrid robot turning a corner. **A** A walking biohybrid robot consisting of a bilayered bioactuator balanced to walk along the bottom of a dish. **B** The speed of the biohybrid robot was consistent for multiple walking tracks. **C** Comparison of the speed of our walking biohybrid robot to prior biohybrid robots. **D**, **E** The walking biohybrid robot was able to navigate around a corner. **F** Analysis of the walking behavior of the biohybrid robot around a corner showed that it took approximately 10 s to make the turn (shaded portion). The frequency of actuation did not change in response to the obstacle (dark purple line). All scale bars are 5 mm.

Next, we probed the ability of our bioactuators to interact with macroscale objects. We hypothesized that the centimeter-scale size and larger range of motion would allow inertia-dominated behaviors. We generated a walking biohybrid robot consisting of a bioactuator with tuned buoyancy so that it would stand and walk along the bottom of a petri dish (Fig. 6A). Interestingly, we found the most efficient propulsion to result from spontaneous twitching of the bioactuator rather than a paced stimulus, likely because the spontaneous contractions were occurring at the optimum pace of 3 Hz while maintaining the same amount of contraction as in the case of pacing (Fig. S15 and Supplementary Movie 5). The robot propelled itself forward at a speed of 6.2 ± 0.6 mm/s based on multiple walking tracks (Fig. 6B), which outperformed all previously reported biohybrid robots in terms of speed normalized to body lengths per minute (Fig. 6C and Table S5).

To test the ability of our walking biohybrid robot to interact with its environment, we used a corner and pillar course (Fig. 6E). When the robot reached the pillar and corner, it maintained its frequency of contraction but slowed to navigate around the corner (Fig. 6F and Supplementary Movie 6). The corner navigation took approximately 10 s (Fig. 6F). The robot's walking speed was similar before and after corner navigation (Fig. 6B). The navigation of the robot was not actively controlled; if the robot did not manage to properly enter the passage between the corner and pillar, it bounced off the front of the pillar (Supplementary Movie 7).

**Fig. 7:**
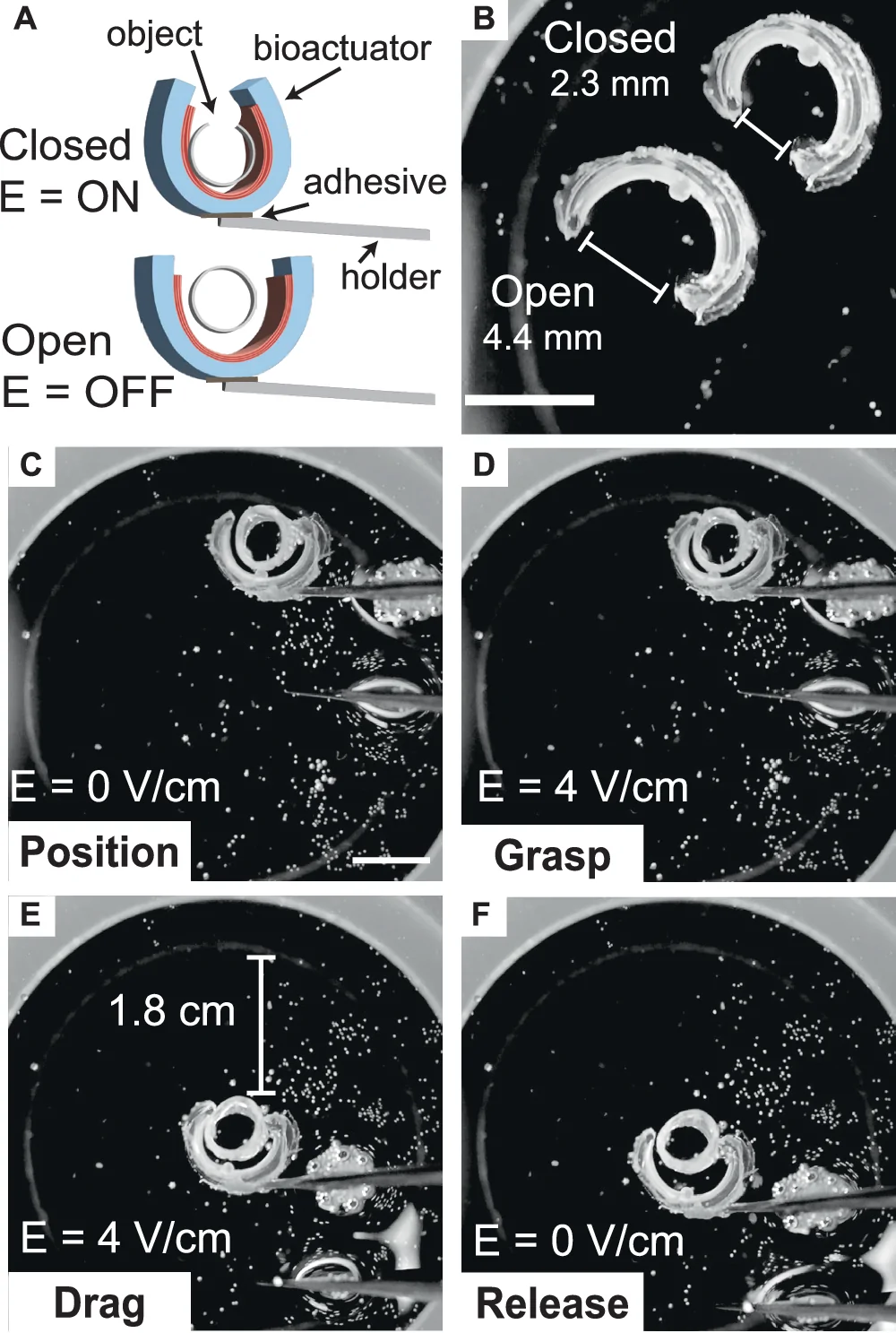
Biohybrid gripper. **A** The biohybrid gripper consisted of a bilayered bioactuator attached to a holding device. The gripper would close around an object when actuated and open when the actuation signal was turned off. **B** The gripper behavior relied on the open and closed states of the bilayered bioactuator. **C** The biohybrid gripper was positioned around an object smaller than the open state. **D** Upon stimulation, the actuator closed around the object, and **E** dragged the object 1.8 cm. **F** After the electrical signal was turned off, the object was released. All scale bars are 5 mm.

We tested whether our bioactuators could facilitate a controllable action by creating a biohybrid gripper (Fig. 7A). The function of the biohybrid gripper relied on the difference between the unactuated, open state of our bioactuator and the actuated, closed state. The biohybrid gripper was produced by adhering a bilayered bioactuator to a holding device that served as an arm to move the gripper (Fig. 7A). In the open state, the gripper had an opening of 4.4 mm (Fig. 7B). Upon application of an electric stimulus to induce a tetanic contraction, the gripper contracted 1.9 mm to have an opening of 2.3 mm (Fig. 7B and Supplementary Movie 8). We chose an object to grip that was 3.3 mm in diameter (Fig. 7C). When actuated, the gripper adapted to the size of the object (Fig. 7D) with a 2.7 mm opening. The object was moved 1.8 cm (Fig. 7E and Supplementary Movie 8) and was able to release the object when the stimulus was turned off (Fig. 7F and Supplementary Movie 8).

### Complex geometry and assembly of biohybrid actuators

**Fig. 8:**
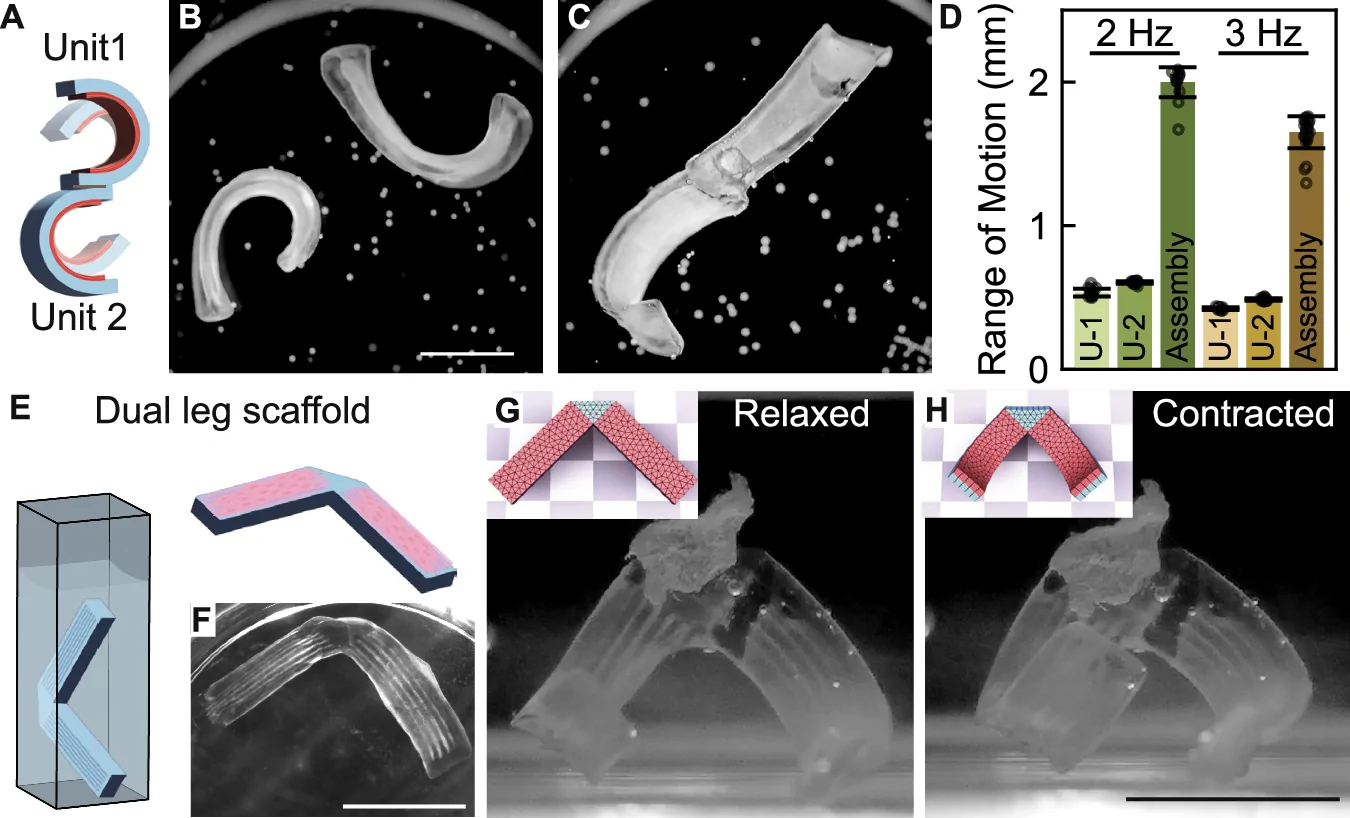
The developed bioactuator modeling and fabrication platform supports modular assembly and complexity in scaffold design. **A** Single units were assembled using a tissue adhesive to attach the scaffold layers of two bilayered bioactuators. **B** The bioactuators were evaluated as single units, and **C** after attachment. **D** The contraction deformation was smallest for the single units and increased for the assembled units. Error bars show standard deviation. **E** Dual-leg bioactuator was volumetrically printed as a single construct, and cells were post-seeded after. **F** Stereomicroscope image of leg bioactuator 24 h after fabrication. **G**, **H** Contraction behavior of a multi-leg bioactuator, with corresponding model shown as inset. All scale bars are 5 mm.

Finally, we moved from single-unit actuators to dual-unit actuators to scale the size and complexity of achievable biohybrid robots (Fig. 8). Single-unit actuators could be assembled post-maturation by using tissue glue on the hydrogel scaffold layer to attach individually matured units without damaging the muscle tissue layer (Fig. 8A–C). As expected, coupling the bioactuators in series increased the observed deformation (Fig. 8D, Fig. S16, and Supplementary Movies 9–10). The contraction time did not change between the single- and dual-unit bioactuators, meaning that multi-unit actuators have a comparatively faster contraction (Fig. S17). Another trend that emerged was that the single-unit and multi-unit actuators had a slightly decreased range of motion at 3 Hz than at 2 Hz. This trend was also true for the swimming biohybrid robots (Fig. 5F) and was reported from 4 Hz for a previous generation of swimming biohybrid robot^30^.

Finally, to demonstrate that our platform can be used for more complex muscle geometries on one construct, we prototyped a bioactuator geometry with two legs attached at a 90° angle (Fig. 8E, F). Notably, the dual-leg bioactuator was fabricated as a single construct, without requiring post-fabrication assembly. The combination of light-based scaffold printing and the developed volumetric bilayer fabrication strategy enables modification of scaffold geometry to tailor the final bioactuator shape, while maintaining the same overall fabrication process. Since we integrated microgrooves onto the scaffold that were parallel to the long edges of the legs, we expected that each leg would contract as an individual unit, perpendicularly to the others. Analysis of the movement behavior showed that this was the case, demonstrating that the microgrooves and material properties of the scaffold are sufficient to determine the direction of muscle contraction for multidirectional alignment (Fig. 8G and Supplementary Movies 11–12). The leg contraction was synchronous when paced, but spontaneous twitching of each leg was not synchronized (Supplementary Movie 11). Further computational optimization of the movement profile, also including the environmental interactions, is a promising strategy to develop more complex motion in the constructs.

## Discussion

In this work, we report the development of a computational and experimental pipeline that co-optimizes the muscle and scaffold geometries of biohybrid actuators according to performance and function. While recent work in the field has focused on increasing the force production of an engineered muscle construct to enable more performant biohybrid robots, our method highlights the importance of matching bioactuator design with its desired function to achieve the full potential of biohybrid robots.

Tissue-engineered muscle can maintain its function in a variety of shapes, given the proper mechanical and chemical cues to develop contractile muscle fibers. Therefore, the geometry and shape of the muscle should be considered design parameters of bioactuators, alongside the properties of the non-living support materials. However, broadening the design space of bioactuators to include the shape and material properties of both the living and non-living components requires a coupled computational modeling method to inform the design of bioactuators, since a trial-and-error approach is too resource- and time-intensive.

Hence, we modeled bioactuators using a soft-body simulation framework that defined contractile and non-contractile elements on a voxel-by-voxel basis. A simple spring-like element was used to model the muscle contraction, which captured the elastic dynamics of muscle^28^. Further, the simulation of a single actuation was fast enough to use a genetic algorithm to evolve bioactuators for range of motion and optimize for the distribution and material properties of muscle and scaffold layers. While the computational model informed the bioactuator design, more accurate modeling of muscle biomechanics and environmental interactions is necessary to enable sim-to-real fidelity. Advantageously, our current framework is customizable and could readily integrate future computational models of muscle function and maturation. Incorporating such models would strengthen the conclusions drawn from our framework, for example, by enabling a more mechanistic investigation of how scaffold stiffness influences bioactuator functionality through the interplay between scaffold material properties and muscle tissue maturation.

An important aspect of translating the computational modeling results to experimental fabrication was the stable biohybrid interface between living and non-living materials. Up to now, for bioactuators made from muscle tissue rather than single cell layers, connection to the scaffold was achieved by mechanical integration around structures^11,12^. In contrast, the bilayer geometry allowed continuous transmission of the muscle contraction force to the hydrogel scaffold. Moreover, we observed an acellular-to-cellular gradient from the scaffold to the highly cellularized upper layer of the muscle tissue. This is reminiscent of the muscle-tendon unit, which has a gradient of increasing stiffness to transmit the muscle contraction to the rigid bone^35^. Furthermore, the microgrooved interface of our scaffold layer is similar to the protrusions visible in the interface of muscle tissue and tendon, which increase surface area for efficient adhesion and force transfer^36,37^.

The fabrication of bioactuators from skeletal muscle tissue is challenging in part because myoblasts must be differentiated in situ to contractile myofibers. This is a complex biological process that requires a balance of biochemical, mechanical, and topographical cues and brings inherent variability to the final engineered muscle tissue^38,39^. In our system, we used a pattern of microgrooves to aid uniaxial alignment of the myofibers. The scaffold also provided a spring-like restoring force to further enhance muscle differentiation, similar to the effect observed in Guix et al.^14^ However, the formation of three-dimensional muscle tissue still has some variability, and our bilayered geometry heightened this variability due to the larger range of possible deformations. We observed final deformations of our bioactuators spanning 0.5–6.6 mm. Future work will focus on better control over the variability to enable rapid and parallelized production of bioactuators for biohybrid robotics. The maturation markers of the final bioactuators were varied: the fusion index was in line with mature engineered muscle constructs^40,41^, but the MyoHC+ myofiber diameter was lower than expected for mature muscle^42^. Therefore, future work to optimize culture conditions and/or topographical cues provided by the scaffold would aid in improving the differentiation process and could improve the bioactuator performance^39,43^.

The fundamental scaling challenge of growing larger muscle tissues is also evident in our work. Histological images showed that cells accumulated in the upper 100 µm of the muscle tissue layer, due to the nutrient and oxygen diffusion limit of approximately 100 µm in engineered muscle tissue^41^. Therefore, we also demonstrated the use of multiple units of our actuators to scale up the deformation or for more complex geometries. Advantageously, our co-optimization framework can readily accommodate biologically motivated constraints such as diffusion-limited tissue thickness, and we identify this as a promising next step to inform the design of larger-scale, perfusable tissues.

Overall, we have presented a different approach for biohybrid actuator design, one that considers the muscle shape and properties as a design parameter instead of a fixed constraint to design around. The computational co-optimization of muscle and scaffold geometry and experimental realization showcased this strategy in the context of engineering bioactuators with a larger range of motion for biohybrid robots that swam, walked, and gripped objects. By showing the scalability and modularity of the fabrication process, we envision that our unified approach to modeling, optimization, and fabrication of biohybrid actuators will accelerate the development of high-performance biohybrid robots.

## Methods

### Computational modeling

The deformable-body simulation solves for a nonlinear neo-Hookean material model for passive tissue materials and a spring-like actuation model for contractile materials^27,44–46^. The stable neo-Hookean formulation^25^ is robust to large deformations, which might cause mesh inversions in the standard neo-Hookean constitutive law. The energy density for each finite element is formulated as follows in Eq. 1.

1 $${\varPsi }_{e}=\frac{\mu }{2}\left({{{\rm{tr}}}}\left({{{{\bf{F}}}}}^{T}{{{\bf{F}}}}\right)-3\right)+\frac{\lambda }{2}{\left(\det \left({{{\bf{F}}}}\right)-\alpha \right)}^{2}-\frac{\mu }{2}{{\mathrm{ln}}}\left({{{\rm{tr}}}}\left({{{{\bf{F}}}}}^{T}{{{\bf{F}}}}\right)+1\right)$$

where **F** is the deformation gradient of the finite element, λ and μ are the rescaled first and second Lamé parameters, and $\alpha=1+\frac{3\mu }{4\lambda }$. Similarly, we can define the actuation energy for muscle elements based on Lee et al.^47^ as Eq. 2.

2 $${\varPsi }_{m}=\frac{k}{2}{\left\|\left(1-r\right){{{\bf{Fm}}}}\right\|}^{2}$$

where we define a stiffness *k* for the muscle, a contractile direction ${{{\bf{m}}}}$, and an actuation signal $r=\frac{1-e}{\left\|{{{\bf{Fm}}}}\right\|}$ such that any actuation level $e\in \left[{{\mathrm{0,1}}}\right]$ corresponds to a percentage of contraction for the muscle element.

These internal energy densities are integrated over the finite element mesh of hexahedrons, after which we solve an energy minimization problem. We define analytical gradients and hessians of the energy *E* to solve the following Newton step iteratively until the increment on vertex positions $\Delta {{{\bf{x}}}}$ is small enough (Eq. 3).

3 $${\nabla }^{2}E\left({{{\bf{x}}}}\right)\,\Delta {{{\bf{x}}}}=-\nabla E\left({{{\bf{x}}}}\right)$$

In our case we solved a time-dependent simulation, adding an inertia term to balance the internal stresses, where we introduced an implicit Euler timestepping scheme for the equations of motion (Eq. 4),

4 $$m\frac{{{{{\bf{v}}}}}_{t}-{{{{\bf{v}}}}}_{t-1}}{\Delta t}=-\nabla {E}_{{{\mathrm{int}}}}\left({{{{\bf{x}}}}}_{t}\right)$$

where we similarly discretized the velocity using implicit Euler (Eq. 5).

5 $${{{{\bf{v}}}}}_{t}=\frac{{{{{\bf{x}}}}}_{t}-{{{{\bf{x}}}}}_{t-1}}{\Delta t}$$

The final energy gradient that we try to find a root for using Newton’s method follows in Eq. 6.

6 $$\nabla E\left({{{{\bf{x}}}}}_{t}\right)=m\frac{{{{{\bf{v}}}}}_{t}-{{{{\bf{v}}}}}_{t-1}}{\Delta t}+\nabla {E}_{{{\mathrm{int}}}}\left({{{{\bf{x}}}}}_{t}\right)!=0$$

### Evolutionary optimization

All designs in the computational optimization were actuated using the same actuation signal, which specifies how much each contractile hexahedral element in the mesh should contract. The actuation signal was chosen to be a smoothed step function that reaches half deformation e=1.0 at 0.25 s, where we let the simulation run until 0.4 s to allow any oscillations to settle. The discretized mesh used in the genetic algorithm contained 600 hexahedral elements, and we quantified the maximum deformation over the simulation time of 0.4 s. The discretized mesh was 2 hexahedral elements tall in the z-direction, and we only allowed the genetic algorithm to allocate contractile material on the xy-plane (30 elements by 10 elements), so along the z-direction, we would not see varying materials. This was mainly done due to computational efficiency to minimize the mesh size. Codes used for modeling can be found here: https://github.com/srl-ethz/bilayered_optimization^48^. Both the simulation framework and the optimization algorithm are provided as open source. This will allow researchers to easily define search spaces and objective metrics for their own applications.

For the optimization, we used the PAGMO package in C + +, running the genetic algorithm “NSGA-II” to search over a binary mask corresponding to the muscle allocation of the hexahedral mesh with a population size of 20 and a distribution index for mutation of 20. The metric we use is the average distance norm of all vertices between the rest shape and the final contracted shape. But we first align the final shape with the initial shape through the Kabsch algorithm^49^ to find an optimal rotation to match the zero-centered meshes. Asymmetric actuation patterns might cause nonzero net torques on the deformable body, which we do not want to consider, and can be removed with this alignment during metric computation.

### Computational analysis of volumetric bilayer designs

The same metric is used as described in the previous section; however, we now change the design space to only consider one muscle group, which we change in width and height. The grid search over the width and height of the muscle presented in Fig. 3B is applied to the same 30 × 10 × 2 voxel grid as for the evolutionary algorithm. To change the stiffness of the scaffold, we ran at a much finer resolution to search over more height ratios, i.e., how many cells in the y-direction are muscle compared to scaffold. For the stiffness sweep in Fig. 3A we ran the simulation with a 15 × 50 × 2 voxel grid for the bioactuator, staying with the same physical dimensions as before, of 15  × 3.5 × 1 mm. The muscle is always full width.

### Pillar-attached bioactuator simulation

To compare a more standard experimental setup for bioactuators, we attached a cuboid with two holes to two stiff pillars and observed the deformation of the pillars in response to the muscle contractions in the bioactuator. We restrict contraction to the x-direction, i.e., the vector pointing from one pillar to the other. Both pillars are modeled with a Young’s modulus of 1 MPa, while, similar to before, the active muscle and passive scaffold in the bioactuator are modeled with 25 kPa stiffness. The cuboid has a resolution of 30 × 20 × 2, with physical dimension 7.5 × 3.5 × 1 mm. We attached the outer edges to the pillars and left one layer of empty space between the inner side of the pillars and the muscle. We used the same actuation signal and simulation times as for the maximal deformation optimization. We changed the objective to be the displacement between the centers of the top surfaces of the pillars.

### Cell culture

Mouse myoblast cell line C2C12 was obtained from ATCC and cultured using growth media consisting of DMEM (Catalog # 41966) with 10% fetal bovine serum (FBS, Catalog #F9665). Cells were passaged after reaching 80% confluency. Cells from passages 4–8 were used for actuator fabrication. All reagents were purchased from Sigma-Aldrich unless otherwise indicated.

### Scaffold preparation

Hydrogel scaffolds were printed using a Xube (xolo, Berlin, Germany). Gelatin methacrylate stock solution was prepared at 10 wt% gelatin methacrylate (X-Pure GelMA 160P80, Rousselot) in 1 M Bis Tris. A 25 mg/mL Stock solution of J-086 (xolo, Berlin, Germany) in polyethylene glycol diacrylate (M.W. 700, PEGDA700) was prepared. To prepare resin for printing, 10 wt% GelMA 160P80 stock solution was diluted 1:2 with desired vol% of PEGDA and the remainder PBS. Photoinitiator was added at 1:1000 dilution. Standard 1 cm cuvettes were filled with 2 mL of resin and allowed to gelate at room temperature. Printing speed and energy were optimized for print fidelity by visual inspection and then kept constant. Prints were isolated by warming and removing into warm PBS. After isolation, prints were washed twice in warm PBS. Prints were post-cured by incubation for 10 min in 0.1 wt% LAP and exposure to 15 mW/cm^2^ 365 nm light for 90 s. Scaffolds were washed twice with PBS to remove LAP and stored overnight in PBS at room temperature,

### Synthesis of gelatin methacrylate (300 g bloom)

Gelatin methacrylate at 300 bloom (GelMA-300) was synthesized^50^. Briefly, a 10 wt% solution of gelatin type A (from porcine skin, gel strength 300 g Bloom) in 0.1 M carbonate bicarbonate buffer was prepared at 50 °C. After the gelatin was fully dissolved, 1280 µL of methacrylic anhydride was added, dropwise, and the reaction was run for 2 h. Then, the solution was split into dialysis tubing (8–12 kDA) and dialyzed for 3 days at 40 °C. Samples were subsequently freeze-dried and stored.

### Bilayered actuator fabrication

Scaffolds were washed in DI water for 30 min. Then, scaffolds were placed onto hydrophobically coated glass slides (Sigma-Cote) and dried for 4 h, including a 30 min UV sterilization cycle.

Stock solutions were prepared of 16.7 wt% gelatin methacrylate (own synthesis, 300 g bloom) in DMEM and 3 wt% LAP in PBS. Cells were mixed to a final concentration of 10 × 10^6^ cells/mL in 5 wt% GelMA, 0.1 wt% LAP, and 66 vol% Matrigel (product no. 354234, Corning). 30 µL of cell hydrogel was deposited onto each dried scaffold. The bilayered constructs were cured for 30 s using 15 mW/cm^2^ 405 nm UV light, then incubated for 30 min at 37 °C. Growth media (DMEM, 10% FBS, 1% P/S) was added, and constructs were incubated overnight. After 24 h, the constructs were gently removed from the hydrophobic glass slides and transferred to 12 well plates. On day 3, the media was switched to differentiation media (DMEM, 10% horse serum, 1% P/S, 5 ng/mL IGF-1). Half of the media volume was replaced daily.

### Stimulation of constructs

Constructs were stimulated in a bath consisting of two graphene electrodes connected to a function generator (#DG812, Rigol, Beijing, China). Electric signals were applied at 3 – 5 V/cm, 1 – 10 Hz frequency, 1% duty cycle.

### Fluorescent staining & imaging

After electrical stimulation, bioactuators were fixed for 1 h in a 4% (v/v) paraformaldehyde solution at room temperature. Then, they were washed in PBS (5 min, 3x) and stained for DAPI, F-actin, and Myosin Heavy Chain (MyoHC). Briefly, samples were permeabilized with 0.1% Triton X-100 for 20 min and blocked with a 1% bovine serum albumin solution for 1 h. Bioactuators were stained with 4’-6-diamidino-2-phenylindol (DAPI, dilution 1:1000; 10 min); ActinRed 555 ReadyProbe reagent (ThermoFisher, No. R37112) for 2 h; and mouse anti-MyHC antibody (Myosin 4, eFluor 660, Clone: MF20, Affymetrix eBioscience) or IgG2b kappa isotype control (eFluor 450, ThermoFisher, No. 48-4732-82) for 8 h. All images were taken using a confocal microscope (Nikon Ti2).

### Histological preparation

After fixation, the samples were embedded in paraffin and sections of 4.5 µm thickness were cut with a microtome (Microm, HM430, Thermo Scientific). The sections were stained with hematoxylin–eosin (#GHS116 and #HT110116, respectively) or Picrosirius Red (#365548) from Sigma-Aldrich. The slides were mounted and imaged in a bright field with a light microscope (Olympus CKX41, Olympus Schweiz AG).

### Mechanical testing

Hydrogel mechanical testing was conducted using an MTS horizontal uniaxial stretching system. Hydrogel strain rate was set to 0.5 mm/s. Video was taken along with force measurements. Real displacement was calculated from video analysis to account for any slippage in the attachment points. Young’s modulus was calculated by calculating the slope of the linear portion (up to 10% strain) of the stress-strain curve. The Young’s modulus of tissue-engineered skeletal muscle was measured from muscle rings produced following the protocol of Raman et al.^43^ (described in the supplementary information) using an Aurora Scientific force measurement system consisting of a force transducer (404 C, Aurora Scientific) coupled with a linear positioner (322D) and the Aurora software (600 A). Young’s modulus was calculated in the linear region of the stress-strain curve.

### Fusion index & myofiber diameter

The fusion index is defined as the number of nuclei within MyoHC+ myotubes divided by the total number of nuclei within a pre-defined region of interest (ROI). Images from three separate samples were used, with three ROIs per image. The same images were used to determine MyoHC+ myofiber diameter, which was measured by using the measurement tool in FIJI to measure the diameter of myofibers in the MyoHC+ channel within a region of interest.

### Statistical methods

All error bars show standard deviation. Multiple group comparisons were conducted using one-way ANOVA and post hoc testing using Tukey’s honest differences test. All values are reported in the text or in the supplementary information.

## Supplementary information

Supplementary Information

Description of Additional Supplementary Files

Supplementary Movie 1: Biohybrid jumping robot (5 V/cm)

Supplementary Movie 2: Biohybrid swimming robot actuated at 1 Hz (3.3 V/cm)

Supplementary Movie 3: Biohybrid swimming robot actuated at 2 Hz (3.3 V/cm)

Supplementary Movie 4: Biohybrid swimming robot actuated at 3 Hz (3.3 V/cm)

Supplementary Movie 5: Walking bilayered bioactuator un-paced and paced contraction at 1 Hz and 2 Hz (4 V/cm)

Supplementary Movie 6: Biohybrid walker traveling around a pillar

Supplementary Movie 7: Biohybrid walker turning in front of pillar

Supplementary Movie 8: Biohybrid gripper actuated by a bilayered bioactuator (4 V/cm)

Supplementary Movie 9: Single bilayered bioactuator paced at 2 and 3 Hz (3 V/cm)

Supplementary Movie 10: Double unit bilayered bioactuator paced at 2 and 3 Hz (3 V/cm)

Supplementary Movie 11: Multi-leg bilayered bioactuator paced at 2 Hz.

Supplementary Movie 12: Multi-leg bilayered bioactuator paced at 3 Hz.

Transparent Peer Review file

## Data Availability

The paper and/or the supplementary information contain all the data needed to evaluate the conclusions. Videos used for analysis are provided as supplementary movies. Correspondence and material requests should be addressed to Robert K. Katzschmann.

## Code availability

Codes used for modeling can be found here: https://github.com/srl-ethz/bilayered_optimization, permanently referenced in a Zenodo repository^48^. Any additional information will be provided upon request.
