# Supplementary material for: Co-optimized volumetric muscle design for large dynamic deformations in versatile biohybrid robots
Source: Nat Commun. 2026 Sep 11;17:10732. doi: 10.1038/s41467-026-77655-1 (PMC13658790; doi:10.1038/s41467-026-77655-1)
Supplement: Supplementary file 1 [file 41467_2026_77655_MOESM15_ESM.pdf]

nature portfolio

Peer Review File

Co-Optimized Volumetric Muscle Design for Large Dynamic Deformations in Versatile Biohybrid Robots

Corresponding Author: Professor Robert Katzschmann

This file contains all reviewer reports in order by version, followed by all author rebuttals in order by version.

Version 0:

Reviewer comments:

Reviewer #1

(Remarks to the Author)
The manuscript presents a computational and experimental approach to the design of biohybrid actuators. While the topic is timely and relevant, and I appreciate the authors' effort to combine modelling with fabrication, I find that the study, in its current form, does not sufficiently demonstrate novelty or generate new conceptual understanding. My detailed comments are outlined below.
1) While I appreciate the benefits of performing computational modelling and the effort behind the literature review in Fig 1A, I'm not completely sure this is a fair comparison. On a first instance, the plot reduces all the characteristics of the biohybrid robots reported to contractile volume and range of motion, while there could be a myriad of other variables that affect their performance. As they claim somewhere else, traditionally the robots have been optimized for force output, or perhaps stress or force per unit length, and not necessarily for range of motion. Some works that they cite, like Pagan-Diaz (2018) or Cvetkovic (2014) are based on a very particular design based on friction with the surface, where large deflections would actually reduce contact area and compromise function. Morimoto (2018) is a complex system that is based on a pulley-like system that translates the force into displacement of a finger-like structure in a less direct way than this current work. Aydin (2019) is a biohybrid robot with a neuromuscular junction, which is even more dissimilar than any of the other ones – the purpose was to demonstrate motion with neuron-activated muscle, not to optimize range of motion. The small range of motion in that paper was probably due to inefficient translation of action potentials into force output, not due to lack of computational modelling of the scaffold and the muscle. Overall, the comparison in Fig. 1A juxtaposes systems with distinct design goals and mechanisms, and therefore does not provide a fair assessment of relative performance.
2) I appreciate the benefit of computational modelling and I fully agree with the authors that it would significantly benefit the biohybrid robotics community. However, the chosen case study does not effectively demonstrate these advantages. For instance, I would argue that the system they have chosen to computationally model is relatively simple and does not necessarily require detailed modelling. In the end, they found that the system achieving the most optimal performance is the one any researcher with knowledge of classical mechanics would have derived using basic principles. I do not see the point (for this particular system) of using a genetic algorithm, since it is very clear that the algorithm would converge to designs like those shown in Figure 2D, as they are the most optimal. The first ones shown in the insets of Figure 2B are either impossible to fabricate or make little mechanical sense (e.g., generations 0 and 50), which, of course, is expected from a genetic algorithm. However, with a system design that lacks complexity like this one, it seems unnecessary. These should have probably been incorporated as design limitations in the algorithm to avoid spending computational time on unrealistic designs. In the end, arriving at an optimal design that is "half scaffold, half muscle" is rather straightforward. Moreover, I believe this particular design does not even require finite element simulation – it could be modelled mathematically, as it is a simple symmetrical design on the XY plane that can be described as a 2D system with two regions of different stiffnesses. The contractions could be modelled as uniaxial forces acting on the muscle side of the system. It would not be elegant, but it is simple enough. The bottom line is: I agree with the authors about the power of computational modelling, but I do not think they have chosen a complex enough model to prove their point – the conclusions remain quite limited. They should have selected a more complex design to really demonstrate its potential.
3) There does not appear to be any clear novelty in the design, as it largely follows the approach of Balciunaite et al. (32). Likewise, there does not seem to be any novelty in the fabrication, or at least it was not sufficiently emphasized in the manuscript. There is some novelty in the final assembly forming a dual bioactuator, but this represents a straightforward attachment and an incremental step rather than a conceptual advance.
4) The authors give much emphasis to the comparison with other works (which as I mentioned before I think it's not a very fair comparison since there are no standard metrics in the field). At this stage, what would truly advance the field is not

competing for a faster or higher-performing robot, but a deeper understanding of how design and modelling choices influence behavior. Since these systems are still far from any practical application, competing on performance metrics that lack standardized definitions adds little value. The work would be more meaningful if it focused on generating knowledge and generalizable principles, rather than on achieving marginal improvements in speed or motion compared to earlier studies with different objectives. In this sense, the focus on competition detracts from what could otherwise be a strong contribution on the computational side.
For these main reasons, I believe the manuscript presents some important limitations in its current form – the limited novelty in design, the lack of new fundamental insights, and the absence of convincing evidence that computational modelling added value given the simplicity of the exemplar (though I fundamentally agree with the concept). I do think that if the authors work to show the benefit of computational modelling, providing more detailed information about this, without focusing that much on the fabrication itself (since that was already published) or achieving the best performance, it could be a strong paper.
Some minor comments:
1) It would be good to explain more details about the simulations, for instance about DiffPD and projective dynamics.
2) Although the fabrication should not necessarily be a focus point of this paper, it would still be good to explain what xolographic printing is, as it’s a less common form of 3D printing.
3) Future versions should prioritize the computational modelling aspect, including explicit details about the genetic algorithm – its type, implementation, parameters, and the rationale behind design choices. This should not only be described in the materials and methods but emphasized as a central contribution, as it would help other researchers implement similar computational approaches in their own designs.
4) It was good to talk about the variability in the designs as they did in page 10-11, as this is a large unaddressed limitation in the field. I’d encourage to make it even more systematic and provide more details about all of the different performances they obtained, sharing as many details as possible.
I genuinely enjoyed reading the paper and I think it has potential - it’s well written and relevant. I appreciate the effort that went into combining computational modelling and experimental work. That said, I feel the focus should shift from proving that the design performs best to showing what can be learned from it. With that adjustment, the study could make a meaningful contribution to how computational approaches are applied in biohybrid systems.

(Remarks on code availability)
The code has a README file with instructions to reproduce. It seems to contain all the files necessary for reproducing the results. However, I haven't attempted to reproduce it now.

Reviewer #2

(Remarks to the Author)
The paper “Co-Optimized Muscle Design for Large Dynamic Deformations in Versatile Biohybrid Robots” presents a computationally guided experimental pipeline for designing biohybrid robots.

The authors' work is interesting, but the overall novelty appears limited. Machine learning and evolutionary algorithms have already been applied to the optimization of biohybrid robots (e.g., Blackiston et al., Sci. Robot. 6, eabf1571, 2021; Hugo Alcaraz-Herrera et al., https://doi.org/10.1007/978-3-031-87639-4_1; Zimmerman et al., Sci. Robot. 10, eadr6472, 2025). Furthermore, the additional elements of the manuscript (e.g., hydrogel choice, muscle maturation level) do not appear to introduce meaningful advancements beyond the current state of the art.

Additional major issues are outlined below:

- In the Introduction, the authors state that “the optimization of centimeter-scale biohybrid actuators accounting for muscle and scaffold material behavior has not yet been achieved.” However, Zimmermann et al. have already demonstrated optimization of macroscopic, ray-like biohybrid devices. The prototypes presented in this paper appear only slightly larger, which does not substantiate a novelty claim in the authors’ approach.

- The algorithm converges on a bilayer design with an inner muscle layer and an outer scaffold layer that bends upon contraction. This result is not particularly surprising and could likely have been derived using simpler analytical models. Similarly, the observation that stiffer scaffolds require thicker muscle layers is intuitive and aligns with prior parametric studies conducted via FEM or analytical approaches. The identification of an optimal muscle volume fraction is potentially interesting, but the authors should justify why this insight could not have been obtained through existing modeling strategies.

- The manuscript compares its results with extrapolations from prior work. This approach is problematic, as existing systems differ substantially in materials, culture conditions, and other critical parameters beyond the muscle–hydrogel volume ratio. To properly validate their hypothesis, the authors should demonstrate that their algorithm outperforms conventional computational or analytical models under consistent conditions, using the same hydrogel formulation, geometry, culture protocol, and other key variables.

- Scaffold stiffness affects not only mechanical deformation but also myotube differentiation and stability, which directly influence force generation. This important biological dimension is not addressed, limiting the validity of the conclusions.

- Key factors such as the hydrogel-tissue interface and the degree of muscle maturation are not incorporated into the algorithm. As these strongly impact device behavior, the omission raises concerns about the reliability and predictive power of the proposed framework.

- The biological data presented are relatively weak. The immunofluorescence images in Figure 3 do not show well-aligned fibers or highly matured tissue, and overall myogenic differentiation appears poor compared with numerous existing studies.

- The hydrogel formulation (gelatin methacrylate + polyethylene glycol diacrylate) is neither new nor optimal for supporting muscle differentiation and functionality.

(Remarks on code availability)

Version 1:

Reviewer comments:

Reviewer #1

(Remarks to the Author)
Thank you to the authors for the comprehensive respond to the comments and revised manuscripts. I believe most of the comments have been addressed and I also appreciate the efforts to improve the computational and methodological details. I will only comment on the design aspects, which is the main contribution of the paper.

The addition of some variation to their initial genetic algorithm definitely makes the results more meaningful. It's indeed quite interesting that optimised design with a tapered muscle towards the edges. I do have to say I did not understand Figure S2 and what it's meant to show. The authors talk about the designs forming bilayers, but all I see is some weird designs. Probably some further explanation in the caption would be useful for those slow like me.

It's also quite interesting to see how the results change when the simulation allows to remove material as well. However, I think this needs now some further explanation, since I wasn't able to locate it in the text. What do you mean exactly with "allowed to remove material"? How does it work? Likewise for the adhesion effects – those are quite interesting and potentially useful, but it's not clear from lines 197-200 in page 9 how does that work exactly in the simulation. Can different levels of adhesion be modelled in your simulation? What values did you choose? What does it mean to "remove connective tissue"?

I find showcasing this potential one of the strengths of the papers, and especially of this revision. The readers would benefit from understanding what's possible in different settings, rather than just focusing too much on a very specific example (such as in the previous version). I'd recommend the authors to streamline this initial part, and show systematically all of these potential case studies and variations. This would diminish the concerns of the case study being "too simplistic" and highlight the potential of the work. After having done that, they can focus on one particular case study for the rest of the experiments as it stands already.

Regarding that, I would add an extra challenge to the authors – which is not compulsory but if it's not too time-consuming it might be worth it. I notice that the simulation with the pillar setup is quite interesting, and manages to add interesting things like having "soft tissue" behind the pillars instead of contractile units (as those are not doing much). But I also notice that is not realistic in the sense that we can never have (at least with current capabilities) a central part full of muscle tissue between both pillars – it needs to be some sort of ring, or the cells would die. I wonder if the authors can add some constraints to their model to, for instance, limit the thickness of the contractile units, to e.g. 200-300 um of thickness. It would be interesting to see if adding these real-life constraints would make their converged designs for realistic (e.g., converging into rings), which would strengthen the potential of their approach. While I understand the authors wanted to emphasize the free search in a not-too-constrained search space, this is already demonstrated by the simpler geometries and then they can proceed to add realistic constraints and the search can still be unconstrained within those limitations.

In sum, as the authors are already showing different variations or applications of their approach, going from the simple bilayer structure to different adhesions, to removing material and the pillar structure, I think the paper would benefit for a more systematic arrangement of those cases that can show the reader what's the potential of their approach. After that, they can focus on one type of design and prove it experimentally. This is just my suggestion – I think it's clear as it is right now (except for those couple of places where I indicated details were missing), but I think it could strengthen the contribution if it's framed like that.

Rafael Mestre

(Remarks on code availability)
Did not review it this second time.

Reviewer #3

(Remarks to the Author)
I sincerely appreciate the efforts made by the authors to address the reviewers' comments and to improve the manuscript. However, my main concerns remain.

I continue to find the work interesting; however, its overall novelty appears limited. As previously noted, machine learning and evolutionary algorithms have already been applied to the optimization of biohybrid robots. The novelty highlighted by the authors, namely, the concurrent volumetric co-optimization of muscle and scaffold material distributions, does not, in my view, constitute a decisive advancement in the field. Moreover, the authors defer the development of "complex architectures," and thus more functional biohybrid robots, to future work. Overall, the level of novelty presented does not appear sufficient for Nature Communications.

The authors have reformulated their claims by emphasizing the concept of a "volumetric bilayer." While it is true that, from both design pipeline and fabrication perspectives, this approach differs from other bilayered scaffolds reported in the literature, it nevertheless remains a bilayer system. As such, it is far from the "volumetric, tissue-like biohybrid muscle actuators" claimed in the manuscript. A truly volumetric, tissue-like structure would exhibit features more closely resembling native tissue, including complex architecture, functional interfaces, and potentially vasculature. In contrast, the proposed design consists of a bilayer structure with an inner muscle layer and an outer scaffold layer that bends upon contraction. This suggests that the computation-informed design approach, although interesting, does not yet lead to outcomes that are sufficiently novel or impactful for the fields of biohybrid devices or skeletal muscle tissue engineering. The additional designs presented by the authors do not resolve this limitation.

I appreciate the authors' efforts to moderate potential overstatements; however, I remain of the opinion that, to properly validate their central hypothesis, namely, the value of computation-informed design, they should demonstrate that their algorithm outperforms conventional computational or analytical approaches under comparable conditions. The absence of such validation weakens the overall claims.

I fully acknowledge that modeling muscle contraction is challenging. Nevertheless, omitting this aspect limits the generalizability of the proposed approach. Furthermore, modeling only the material response to muscle contraction could have been achieved using simpler methods, as noted in previous comments.

I appreciate the additional biological analyses performed by the authors; however, these remain relatively limited and point to a muscle structure that is not yet well matured. Although MyoHC+ myofibers are now presented, the fibers do not appear well aligned and instead show a largely random orientation, nor do they indicate a high degree of maturation. Appropriate controls should be included to confirm the specificity of the MyoHC staining. In addition, clearer images, and a sufficient number of them, should be provided to distinguish nuclei incorporated within myotubes from those that are not, thereby supporting the fusion index calculation, which cannot be reliably assessed from the current data. Further analyses would also be beneficial, such as imaging of sarcomeric organization and mRNA-based assays.

(Remarks on code availability)

Version 2:

Reviewer comments:

Reviewer #1

(Remarks to the Author)
Thank you to the authors for their comprehensive review. I believe the authors have adequately addressed all the comments and the manuscript is suitable for publication in its current form. Congratulations for the work.

(Remarks on code availability)

Reviewer #2

(Remarks to the Author)
I appreciate the authors' responses, the clarifications provided, and the additional data included in the revised manuscript. Overall, I find the revisions convincing and believe that the authors have satisfactorily addressed my concerns.

(Remarks on code availability)

Open Access This Peer Review File is licensed under a Creative Commons Attribution 4.0 International License, which permits use, sharing, adaptation, distribution and reproduction in any medium or format, as long as you give appropriate credit to the original author(s) and the source, provide a link to the Creative Commons license, and indicate if changes were made.
In cases where reviewers are anonymous, credit should be given to 'Anonymous Referee' and the source.
The images or other third party material in this Peer Review File are included in the article's Creative Commons license, unless indicated otherwise in a credit line to the material. If material is not included in the article's Creative Commons license and your intended use is not permitted by statutory regulation or exceeds the permitted use, you will need to obtain permission directly from the copyright holder.
To view a copy of this license, visit https://creativecommons.org/licenses/by/4.0/

Rebuttal to Reviewer Comments – Nature Communications submission

‘Co-Optimized Volumetric Muscle Design for Large Dynamic Deformations in Versatile Biohybrid Robots’ (Manuscript ID NCOMMS-25-73697A)

Response to Reviewers:

Reviewer #1 (Remarks to the Author):

Reviewer 1-0: The manuscript presents a computational and experimental approach to the design of biohybrid actuators. While the topic is timely and relevant, and I appreciate the authors’ effort to combine modelling with fabrication, I find that the study, in its current form, does not sufficiently demonstrate novelty or generate new conceptual understanding. My detailed comments are outlined below.

Answer 1-0: We thank the reviewer for their careful reading of our manuscript and for their constructive feedback. We appreciate the recognition of the relevance of combining computational modeling with experimental biohybrid actuator design. We understand the

concern that the previous version may not have sufficiently highlighted the novelty or conceptual contribution of the work. In the revised manuscript, we have clarified the main methodological advance of the co-optimization of volumetric muscle and scaffold designs and adjusted the emphasis to better communicate the insights enabled by this approach. We address the specific points raised in detail below.

Reviewer 1-1: While I appreciate the benefits of performing computational modelling and the effort behind the literature review in Fig 1A, I'm not completely sure this is a fair comparison. On a first instance, the plot reduces all the characteristics of the biohybrid robots reported to contractile volume and range of motion, while there could be a myriad of other variables that affect their performance. As they claim somewhere else, traditionally the robots have been optimized for force output, or perhaps stress or force per unit length, and not necessarily for range of motion. Some works that they cite, like Pagan-Diaz (2018) or Cvetkovic (2014) are based on a very particular design based on friction with the surface, where large deflections would actually reduce contact area and compromise function. Morimoto (2018) is a complex system that is based on a pulley-like system that translates the force into displacement of a finger-like structure in a less direct way than this current work. Aydin (2019) is a biohybrid robot with a neuromuscular junction, which is even more dissimilar than any of the other ones – the purpose was to demonstrate motion with neuron-activated muscle, not to optimize range of motion. The small range of motion in that paper was probably due to inefficient translation of action potentials into force output, not due to lack of computational modelling of the scaffold and the muscle. Overall, the comparison in Fig. 1A juxtaposes systems with distinct design goals and mechanisms, and therefore does not provide a fair assessment of relative performance.

Answer 1-1: We thank the reviewer for the comprehensive analysis of Fig. 1A, and we agree that the other literature examples had distinct design goals. To better clarify this in the text, we have removed the performance comparison from Fig. 1 and changed the figure to focus on the computational and experimental pipeline for muscle and scaffold co-optimization (see above, Fig. R1). Correspondingly, we have shifted the placement and focus of the comparison to Fig. 4 (see Fig. R2). The purpose of the literature comparison is to demonstrate the effectiveness of our design for range of motion even with larger tissue volumes, without claiming that one design is superior compared to another. To ensure this point is understood and to prevent any possible overclaims or misinterpretation, we have updated the description of the performance comparison:

"*Importantly, the previously reported bioactuators were not optimized and validated for the range of motion by a computational pipeline. Therefore, we highlight that by using our computational pipeline to co-optimize muscle and scaffold geometry and developing a suitable fabrication method that enabled continuous force transmission across the*

biohybrid muscle interface, our bioactuators demonstrated a substantial increase in range of motion for actuators with similar contractile volumes" (Lines 292 – 297).

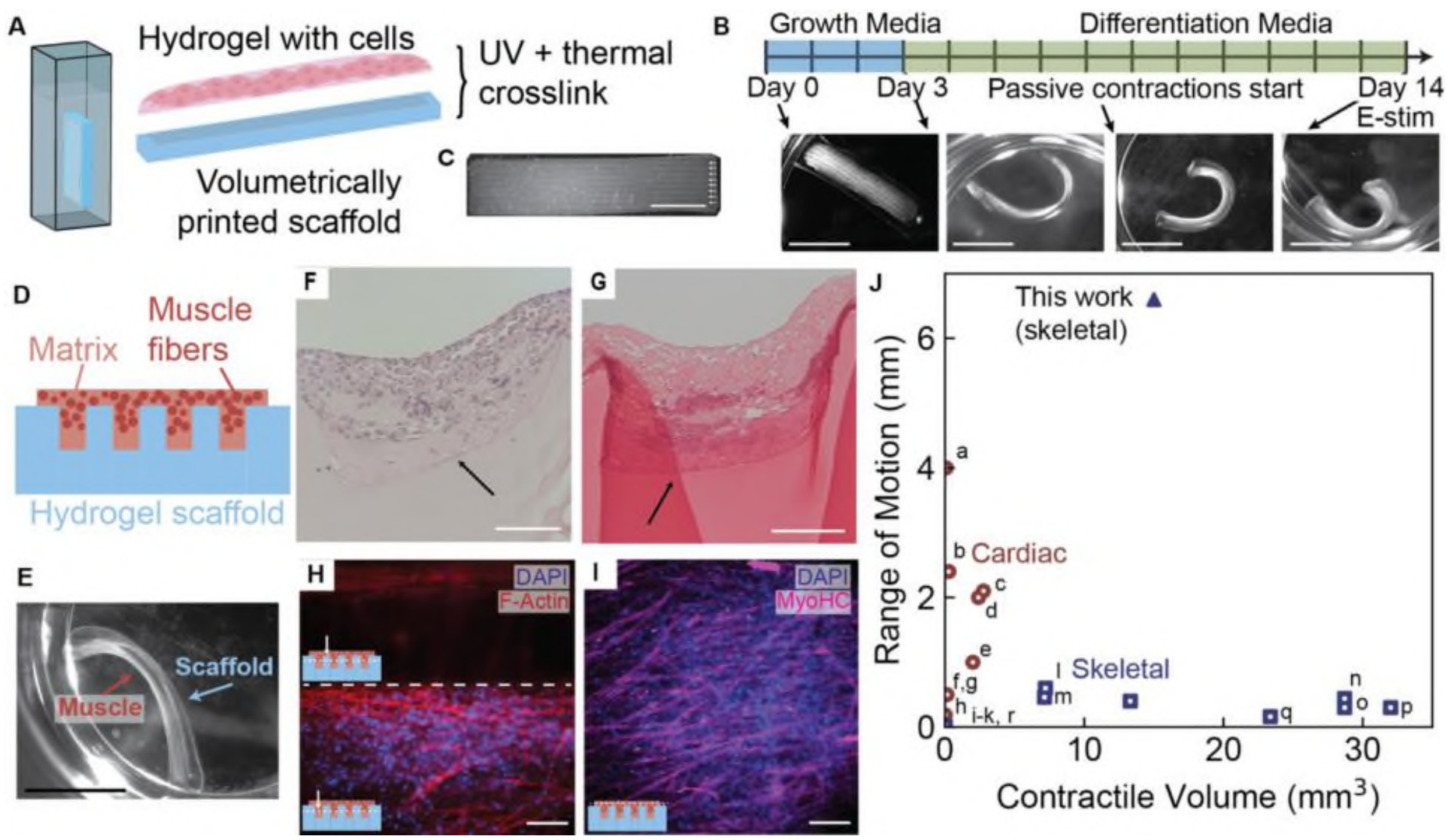

Figure R2 | Figure 4: **Volumetric bilayer bioactuator fabrication and characterization. (A)** Bioactuators (15 mm length, 3.5 mm width, 1.5 mm height) were prepared by post-seeding a hydrogel containing C2C12 mouse myoblasts to a hydrogel scaffold and cross-linking with UV and temperature. **(B)** After fabrication, bioactuators were grown and differentiated using a two-week protocol. **(C)** Image of the hydrogel scaffold prior to seeding, with arrows indicating the grooves. **(D)** Sketch of the cross-section of muscle and scaffold layers. Groove dimensions are 300 µm in width, 500 µm in depth, and 12 mm in length. **(E)** Labeled stereomicroscope image of the bioactuator (bottom). **(F)** Histological cross-section stained with H&E of a groove, showing the distribution of cells in the muscle layer (purple nuclei), corresponding to the view in panel D. The scaffold-muscle interface is indicated with an arrow. **(G)** Histological cross-section stained with picrosirius red showing a groove, corresponding to the view in panel D **(H)** Confocal microscopy image of the lower part of the muscle layer, showing myofibers stained with F-actin (red) inside of a groove. Dashed line shows the border of a microgroove. Cell nuclei are shown in blue. **(I)** Confocal microscopy image of upper muscle layer of bioactuator, with mature myofibers stained with MyoHC (purple) and nuclei stained in blue (DAPI). **(J)** Comparison of the maximum range of motion for bioactuators of different contractile volumes. See Table S1 for values and references of previously reported biohybrid robots. Scale bars in B, C, and E are 5 mm, scale bars in F, G, H, I are 100 µm.

Reviewer 1-2: I appreciate the benefit of computational modelling and I fully agree with the authors that it would significantly benefit the biohybrid robotics community. However, the chosen case study does not effectively demonstrate these advantages. For instance, I would argue that the system they have chosen to computationally model is relatively simple and does not necessarily require detailed modelling. In the end, they found that

the system achieving the most optimal performance is the one any researcher with knowledge of classical mechanics would have derived using basic principles. I do not see the point (for this particular system) of using a genetic algorithm, since it is very clear that the algorithm would converge to designs like those shown in Fig. 2D, as they are the most optimal. The first ones shown in the insets of Fig. 2B are either impossible to fabricate or make little mechanical sense (e.g., generations 0 and 50), which, of course, is expected from a genetic algorithm. However, with a system design that lacks complexity like this one, it seems unnecessary. These should have probably been incorporated as design limitations in the algorithm to avoid spending computational time on unrealistic designs. In the end, arriving at an optimal design that is "half scaffold, half muscle" is rather straightforward. Moreover, I believe this particular design does not even require finite element simulation – it could be modelled mathematically, as it is a simple symmetrical design on the XY plane that can be described as a 2D system with two regions of different stiffnesses. The contractions could be modelled as uniaxial forces acting on the muscle side of the system. It would not be elegant, but it is simple enough. The bottom line is: I agree with the authors about the power of computational modelling, but I do not think they have chosen a complex enough model to prove their point – the conclusions remain quite limited. They should have selected a more complex design to really demonstrate its potential.

Answer 1-2: We agree that analytical models could identify the optimal ratio if a bilayer structure is assumed *a priori*. However, our approach does not impose such structural assumptions. In particular, analytical models typically require predefined geometries or simplified bilayer assumptions, and therefore cannot easily explore (i) heterogeneous voxel-level allocation of active and passive materials, (ii) multiple muscle groups with arbitrary topology, or (iii) simultaneous geometry–material co-optimization within a volumetric design space. Our approach searches the allocation of muscle within the scaffold where placement can be completely arbitrary across one or more muscle groups. Exploring this design space requires a global optimization method together with a computational model that is expressive enough, such as the finite element model used in this work. To strengthen this point of expressive designs being enabled through our FEM approach, we adjusted the genetic algorithm (a different implementation where we increased the mutation rate) and ran it until convergence (as seen in the updated Fig. 2, Fig. R3 copied below). The final shape is less intuitive now, with a tapered muscle towards the edges of the scaffold. We also added an analysis on the adhesion effects in Fig. 3 (Fig. R4, copied below) and further supplementary results in Fig. S2, which ran the optimization that is allowed to remove material as well, both showing the generality of our FEM approach. In Fig. S2, we see how bilayers form as thin as possible, which we further analyze in Fig. 3 to see how this ratio of muscle and scaffold changes with stiffness (Lines 151 – 155, copied below). In conclusion, we agree that for simplified analysis, assuming

bilayered structures as a 2D mathematical model would suffice, however, for the optimization, we stay as general as possible to maximize the expressiveness of the search, and this is where our FEM shows its strength, as well as for future, more complex applications.

"*We fixed the search space to a cuboid domain, since our additional experiments* ***(Fig. S2)*** *showed a convergence towards as-thin-as-possible two-layered structures if material is allowed to be removed, and we would like to further understand the muscle allocation within such thin bioactuators.*" (Lines 151 – 155)

To further demonstrate the generalizability of our approach, we have included a comparative analysis of a bioactuator around pillars in Fig. 2B (Fig. R3, copied below), showing that our algorithm is able to solve for passive and active element distribution in a different geometry. We describe this comparative case in the text in lines 163 – 166.

"As a comparative case, we demonstrated the applicability of our algorithm to common biohybrid set-ups like a bioactuator around two pillars, maximizing for pillar deformation ***(Fig. 2B).*** *In this case, our algorithm converged to a muscle and scaffold distribution where passive elements were on the outside of the pillars, while the remainder of the structure is muscular."* (Lines 163 – 166)

We agree with the reviewer, that more constraints could have been added during optimization to only produce feasible designs to fabricate. With more complex designs in the future, especially with multiple bioactuators assembled in a larger structure, we imagine that such constraints can speed up computation significantly. Since our genetic algorithm converges to an optimal design within half an hour, searching a Boolean parameter space of 600 variables, we did not think of adding further constraints to the design space, but this will be considered for future work.

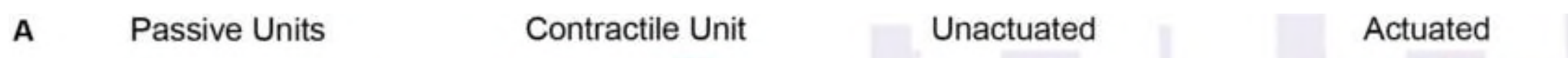

Figure R3 | Figure 2: Computational co-optimization of the volumetric muscle and scaffold elements of biohybrid actuators. (A) We defined biohybrid actuators in simulation as objects consisting of contractile and non-contractile materials. **(B)** We used an evolutionary algorithm to optimize for a geometry that generated the maximum range of movement (structure deformation). Examples of the actuator at generation 0, 25, 150, and 400 are shown above the plot. As a comparative case, we used our method to optimize the bioactuator geometry for maximum deformation of pillars (y-axis on the right), a common biohybrid setup. The pillars were modeled as a stiff, passive material. Insets in the graph show the original randomized design and the final optimized bioactuator design.

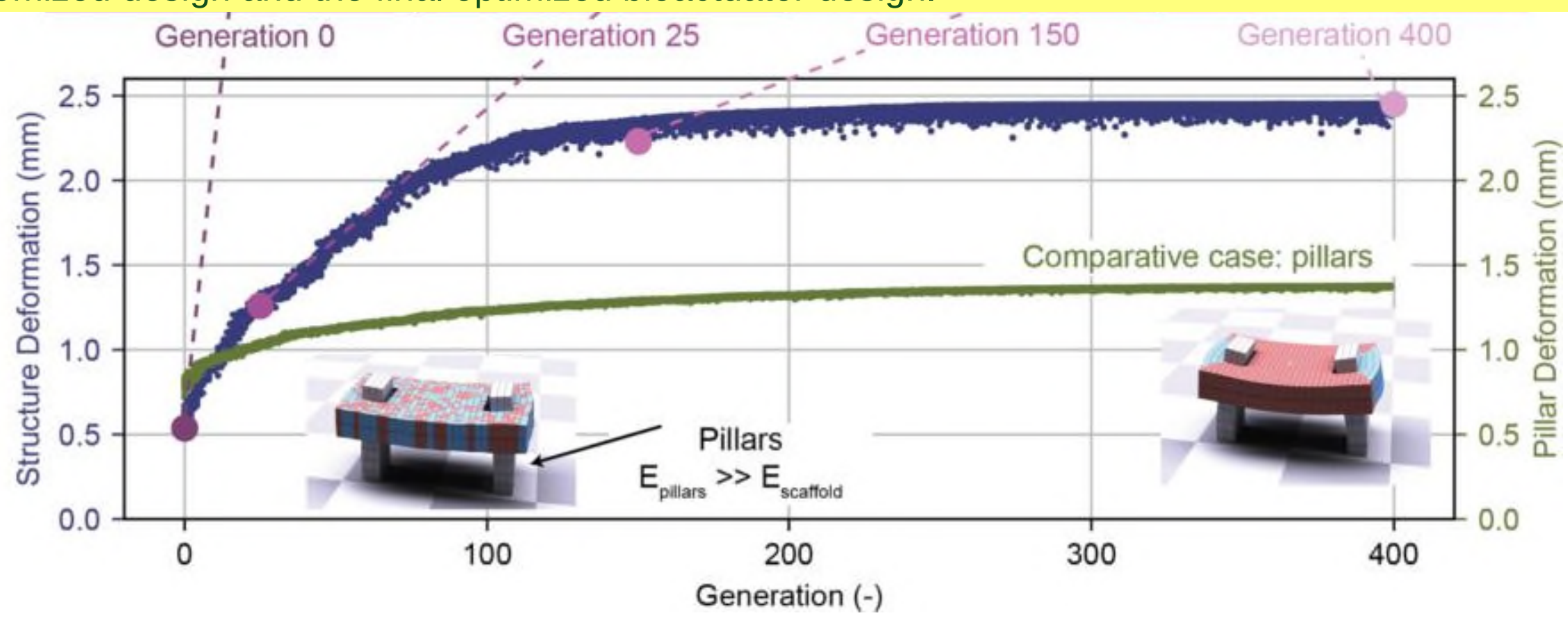

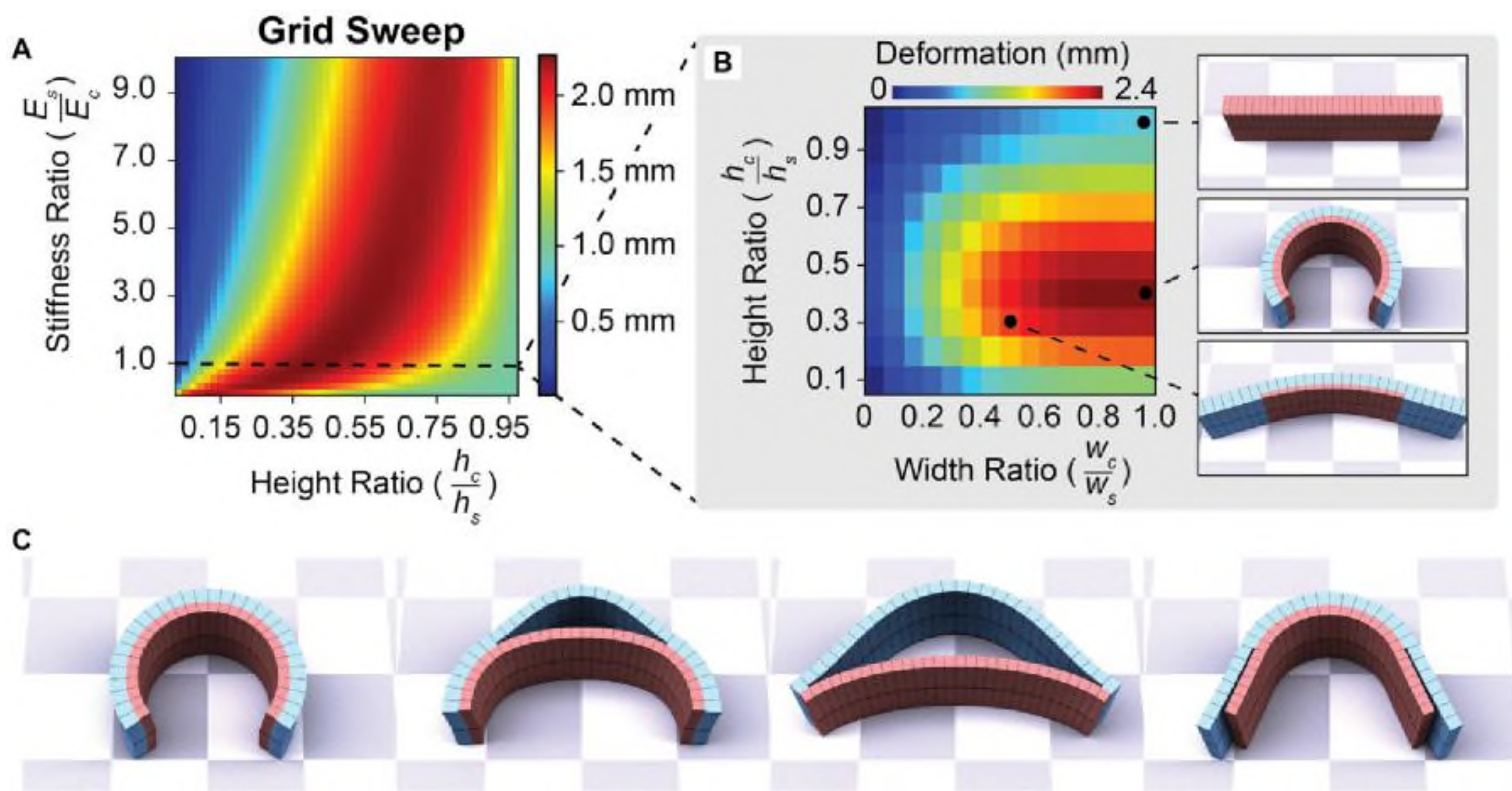

Figure R4 | Figure 3: Computational analysis on designing volumetric bilayer bioactuators. (A) Assuming a bilayered design, the distribution of muscle and scaffold of the bioactuator was refined by a grid optimization study to look at the effect of scaffold stiffness ratios and height ratios on deformation. **(B)** For a fixed ratio of 1 between the scaffold and contractile unit stiffnesses, the optimal bioactuator geometry was where the contractile layer comprises 40% of the height and spans 100% of the length. **(C)** Various structures where the muscle detaches from the scaffold. From left to right: A fully connected bilayer deforming 2.44mm, approximately 50% of the muscle is detached in the middle deforming 1.70mm, only the two ends are attached between muscle and scaffold deforming 1.46mm, and lastly roughly half the muscle is detached on the outer edges deforming 2.16mm.

Reviewer 1-3: There does not appear to be any clear novelty in the design, as it largely follows the approach of Balciunaite et al. (32). Likewise, there does not seem to be any novelty in the fabrication, or at least it was not sufficiently emphasized in the manuscript. There is some novelty in the final assembly forming a dual bioactuator, but this represents a straightforward attachment and an incremental step rather than a conceptual advance.

Answer 1-3: We agree with the reviewer that the results from our previous conference paper serve as the foundation for our current manuscript. We would like to emphasize two points in this regard. First, we identify the main novelty of our paper to be the volumetric co-optimization of scaffold and muscle, rather than the fabrication method of the bioactuator itself. To clarify this further, we have changed Fig. 1 (see Fig. R2 in this document) and lines 102 – 109. We additionally investigate the capabilities and versatility of the developed actuators through new several biohybrid robots presented in Figs. 5 – 8. The fabrication method itself was optimized in this paper compared to our previous work by changing the hydrogel composition to increase yield from 50% to 85%, as mentioned in lines 240 – 242. This improved fabrication robustness enabled the

realization and experimental evaluation of a broader set of robotic motions and bioactuator geometries presented in this manuscript.

"We designed biohybrid systems through volumetric co-optimization of muscle and scaffold elements using a soft body simulation framework combined with an evolutionary algorithm (Fig. 1). We evolved actuator geometries to maximize range of motion, finding a volumetric bilayered architecture. As a comparative case, we showed that our algorithm can be used for optimizing pillar deformation with volumetric muscle, a common biohybrid geometry. In step 2, we fabricated volumetric bilayers by stably integrating a volumetric muscle layer to a hydrogel scaffold. Finally, we experimentally demonstrated the advantages of the optimized bioactuators through diverse locomotion and manipulation tasks and further illustrated scalability through modular assembly." (Lines 102 – 109)

Further, we have clarified the dual-leg bioactuator setup as presented in Fig. 8 because it included both: a dual-unit bioactuator obtained by attaching two individual units after biofabrication; and a dual-unit bioactuator biofabricated as a single construct. These examples illustrate how the developed modeling and fabrication pipeline enables both modular assembly and direct fabrication of more complex biohybrid structures. As far as we are aware, we demonstrate the first case of a functional multi-directionally aligned tissue volumetric bioactuator that did not require assembly, presenting an advancement over the state-of-the-art fabrication that was previously limited to thin film cardiac layers.

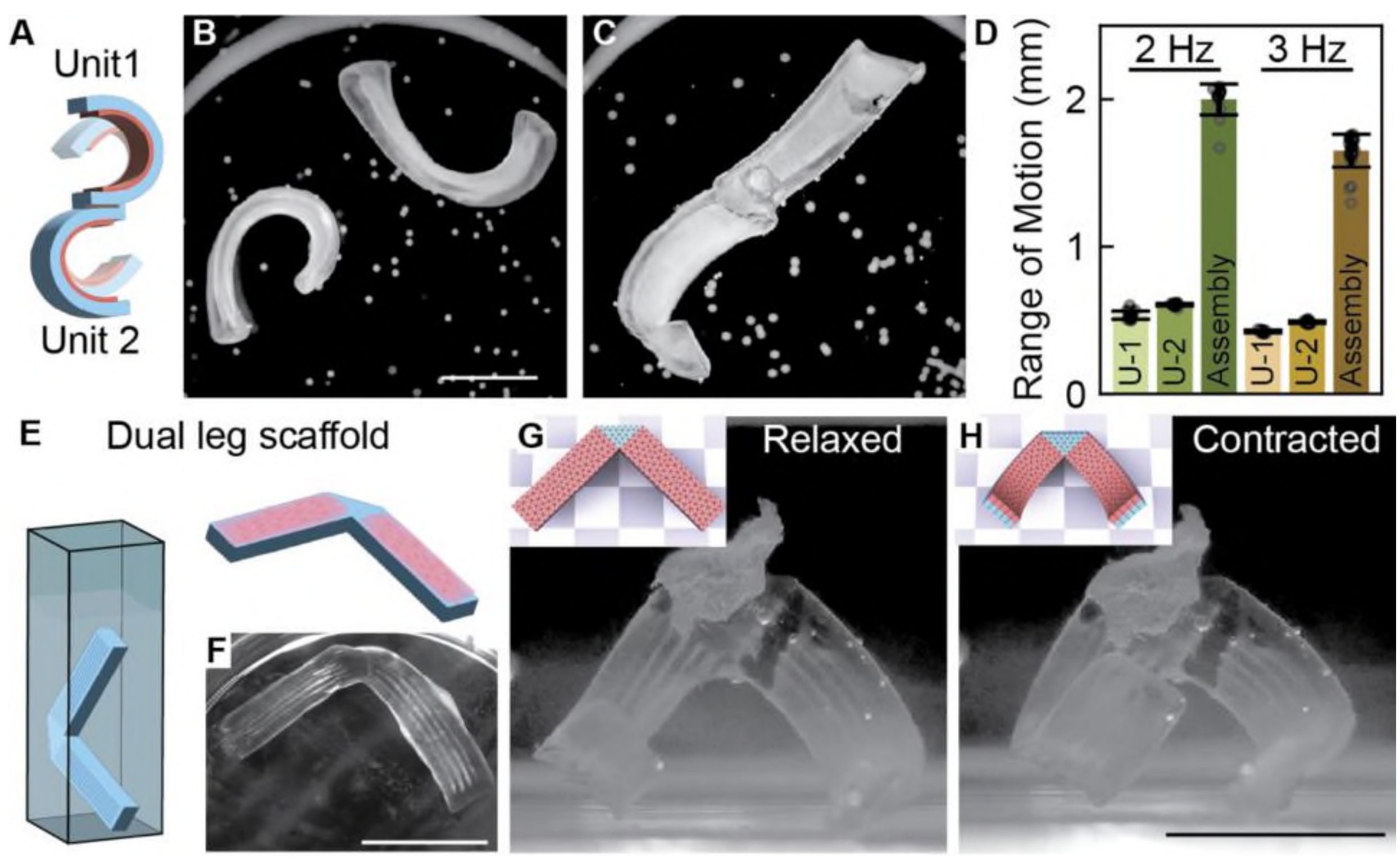

Figure R5 | Figure 8: The developed bioactuator modeling and fabrication platform supports modular assembly and complexity in scaffold design. (A) Single units were assembled using a tissue adhesive to attach the scaffold layers of two bilayered bioactuators. **(B)** The bioactuators were evaluated as single units, and **(C)** after attachment. **(D)** The contraction deformation was smallest for the single units and increased for the assembled units. **(E)** Dual-leg bioactuator was volumetrically printed as a single construct, and cells were post-seeded after. **(F)** Stereomicroscope image of leg bioactuator 24 hours after fabrication. **(G – H)** Contraction behavior of a multi-leg bioactuator, with corresponding model shown as inset. All scale bars are 5 mm.

"*Notably, the dual-leg bioactuator was fabricated as a single construct, without requiring post-fabrication assembly . The combination of light-based scaffold printing and the developed volumetric bilayer fabrication strategy enables modification of scaffold geometry to tailor the final bioactuator shape, while maintaining the same overall fabrication process."* (Lines 406 – 409)

Reviewer 1-4: The authors give much emphasis to the comparison with other works (which as I mentioned before I think it's not a very fair comparison since there are no standard metrics in the field). At this stage, what would truly advance the field is not competing for a faster or higher-performing robot, but a deeper understanding of how design and modelling choices influence behavior. Since these systems are still far from any practical application, competing on performance metrics that lack standardized definitions adds little value. The work would be more meaningful if it focused on generating knowledge and generalizable principles, rather than on achieving marginal improvements in speed or motion compared to earlier studies with different objectives. In this sense, the focus on competition detracts from what could otherwise be a strong contribution on the computational side.

Answer 1-4: Thanks for this comprehensive comment about the purpose of performance metrics. We agree with the reviewer that the previous version of our manuscript placed too much emphasis on competition, while our purpose was instead to show that the co-optimized design of scaffold and muscle in biohybrid systems is a useful tool. Therefore, we have positioned the comparison with previous works as part of Fig. 4 instead. It should serve the purpose of showing that co-optimization of scaffold and tissue is a useful approach, while avoiding the overclaim of having the "best" approach. To help readers correctly interpret this and avoid any unintended overclaims, we revised Figs. 1 – 3 and made corresponding changes in the text as detailed in the responses above. Further, we have revised the description of the performance metrics in the text to be clearer about the purpose in lines 285 – 297.

"We compared the range of motion achieved in our work with previously reported biohybrid robots to evaluate whether the volumetric bilayered shape effectively improved

range of motion, as predicted by the computational modeling pipeline ***(Fig. 4J).*** *An analysis of biohybrid robots published over the last two decades shows that bioactuators with increased tissue contractile volume exhibit a decreased absolute range of motion. Tissue contractile volume was taken from the text or estimated based on images (****Table S1****). For purposes of comparison, the largest deformation of the biohybrid actuator was taken to be the largest movement of the actuator; for planar actuators, this would be a bending motion. Importantly, the previously reported bioactuators were not optimized and validated for range of motion by a computational pipeline. Therefore, we highlight that by using our computational pipeline to co-optimize muscle and scaffold geometry and developing a suitable fabrication method that enabled continuous force transmission across the biohybrid muscle interface, our bioactuators demonstrated a substantial increase in range of motion for actuators with similar contractile volumes." (Lines 285297)*

Reviewer 1-5: For these main reasons, I believe the manuscript presents some important limitations in its current form – the limited novelty in design, the lack of new fundamental insights, and the absence of convincing evidence that computational modelling added value given the simplicity of the exemplar (though I fundamentally agree with the concept). I do think that if the authors work to show the benefit of computational modelling, providing more detailed information about this, without focusing that much on the fabrication itself (since that was already published) or achieving the best performance, it could be a strong paper.

Answer 1-5: We thank the reviewer for the feedback. We have applied the above suggested corrections to make sure that the paper better highlights the benefit of computational modeling and de-emphasizes the aspect of performance.

Reviewer 1-6: Some minor comments:

Reviewer 1-6.1: It would be good to explain more details about the simulations, for instance about DiffPD and projective dynamics.

Answer 1-6.1: As part of the revision process, we have shifted to using our own simulation framework. This is detailed in the "Computational Modeling" section in "Materials and Methods", where we describe the energy density formulations for our passive Neohookean material and active contractile tissue. We detail the implicit Euler timestepping as well as the Newton's method we used to solve for the dynamics. We believe this level of detail for the simulations will be sufficient for the reader to clearly understand the principle and replicate the method in their specific experimental scenarios.

Reviewer 1-6.2: Although the fabrication should not necessarily be a focus point of this paper, it would still be good to explain what xolographic printing is, as it's a less common form of 3D printing.

Answer 1-6.2: We agree on this observation. To address this point, we have added lines 231 – 236 in the main text to explain xolographic printing and cited the relevant works for interested readers.

"Building on the concept of a volumetric bilayer bioactuator first introduced in Balciunaite et al. (31), microgrooved hydrogel scaffolds were prepared by xolographic printing (32), a linear volumetric printing technique recently adapted for use with hydrogels (33-35). Advantageously, the use of xolography allowed quick prototyping of scaffold geometries with microscale resolution, as each scaffold took approximately 60s of printing and the fabrication method was robust to changes in geometry." (Lines 231 – 236)

Relevant references of the new text addition

33. L. Stoecker, G. Cedillo-Servin, N. F. König, F. V. de Graaf, M. García-Jiménez, S. Hofmann, K. Ito, A. S. Wentzel, M. Castilho, Xolography for Biomedical Applications: Dual-Color Light-Sheet Printing of Hydrogels With Local Control Over Shape and Stiffness. *Advanced Materials* **37**, 2410292.

34. A. Wolfel, C. Johnbosco, A. Anspach, M. Meteling, J. Olijve, N. F. König, J. Leijten, Bioxolography Using Diphenyliodonium Chloride and N-Vinylpyrrolidone Enables Rapid High-Resolution Volumetric 3D Printing of Spatially Encoded Living Matter. *Advanced Materials*, doi: 10.1002/adma.202501052 (2025).

35. E. Brauer, A. Balciunaite, M. R. Kollert, J. Weihs, R. S. Knecht, R. Behncke, S. Quach, N. Felix König, A. Badolato, S. Monestier, S. Bersini, M. Moretti, M. Filippi, R. Hägerling, M. Rezvani, S. Hecht, A. Petersen, R. K. Katzschmann, Biocompatible Ink Optimization Enables Functional Volumetric Bioprinting With Xolography. *Advanced Materials* **38**, e12058.

Reviewer 1-6.3: Future versions should prioritize the computational modelling aspect, including explicit details about the genetic algorithm – its type, implementation, parameters, and the rationale behind design choices. This should not only be described in the materials and methods but emphasized as a central contribution, as it would help other researchers implement similar computational approaches in their own designs.

Answer 1-6.3: We find this to be a very valid point concerning information that was not easy to find in the original manuscript. We have added further details to the main text about the design choices, in lines 142 – 161 as copied below. We have added also expanded on implementation details and design choices in "Evolutionary Optimization" in the "Materials and Methods" in lines 483 – 543, copied below. The implementation is fully open-source and should allow any user to customize the search space and objective to their own application.

"An individual simulation took less than 5 seconds, which allowed us to couple the bioactuator model with a parallelized evolutionary algorithm to optimize a generic rectangular bioactuator for deformation ***(Fig. 2B****). We used the non dominated sorting genetic algorithm (NSGA-II) from the PAGMO (Parallel Global Multiobjective Optimizer) package (29) to search a binary voxel grid determining whether a finite element, a hexahedron in this case, is an actively actuated muscle or not. The genetic algorithm uses a population size to run multiple simulations in parallel; we fixed this value at 20. We encouraged a broader, more random, search space by changing the default setting of the distribution index for mutation from 50 to 20. This can be seen in* ***Fig. 2B*** *as the different samples in a population have a wide spread, whereas the default mutation distribution index setting would result in a too exploitative search that might converge to a local optimum. We fixed the search space to a cuboid domain, since our additional experiments* ***(Fig. S2)*** *showed a convergence towards as-thin-as-possible two-layered structures if material is allowed to be removed, and we would like to further understand the muscle allocation within such thin bioactuators.*

We randomly seeded an initial generation and continuously permuted the assembly of contractile and scaffold elements selected for maximum deformation. To quantify the deformation, we observed the contraction after 0.25s of simulation time and then waited until it had fully stopped oscillating after a total of 0.4s. The maximal deformation was calculated as the maximum distance between the initial and the final mesh vertex positions. The final algorithm converged on a bilayered geometry with an inner muscle layer and an outer scaffold layer that resulted in a bending motion upon contraction of the inner active layer." (Lines 142 – 161)

Addition to materials and methods:

"Computational Modeling

The deformable-body simulation solves for a nonlinear Neohookean material model for passive tissue materials and a spring-like actuation model for contractile materials as described in previous work (27, 44–46). The stable Neohookean formulation (25) is robust to large deformations which might cause mesh inversions in the standard Neohookean constitutive law. The energy density for each finite element is formulated as follows:

$$\Psi = \frac{\mu}{2}(tr(F^TF) - 3) + \frac{\lambda}{2}(det(F) - \alpha)^2 - \frac{\mu}{2}ln(tr(F^TF) + 1)$$

where F is the deformation gradient of the finite element, λ and μ are the rescaled first and second Lamé parameters, and $\alpha = 1 + \frac{\mu}{\lambda}$. Similarly, we can define the actuation

energy for muscle elements based on Lee et al. (47)

as:

$$k$$

$$\Psi(=$$

where we define a stiffness k for the muscle, a contractile direction m, and an actuation signal $r = $)! |-(| such that any actuation level e E [0,1] corresponds to a percentage of contraction for the muscle element.*

These internal energy densities are integrated over the finite element mesh of hexahedrons, after which we solve an energy minimization problem. We define analytical gradients and hessians of the energy E to solve the following Newton step iteratively until the increment on vertex positions Δx is small enough:

$$\nabla \# E(x) \; \Delta x = - \nabla E(x)$$

In our case we solved a time-dependent simulation, adding an inertia term to consider, where we introduced an implicit Euler time stepping scheme for the equations of motion:

$$m \frac{v_. - v_{.*)}}{\Delta t} = - \nabla E/0.(x.)$$

*where we similarly discretized the velocity using implicit Euler $v. = $ 1!*1!"# 2.. The final energy gradient that we try to find a root for using Newton's method is:*

$$\nabla E(x.) = m \frac{v_. - v_{.*)}}{\Delta t} + \nabla E/0.(x.) \; ! = 0$$

Evolutionary Optimization

All designs in the computational optimization were actuated using the same actuation signal, which specifies how much each contractile hexahedral element in the mesh should contract. The actuation signal was chosen to be a smoothened step function that reaches half deformation $e = 1.0$ at 0.25s, where we let the simulation run until 0.4s to allow any oscillations to settle. The discretized mesh used in the genetic algorithm contained 600 hexahedral elements and we quantified the maximum deformation over the simulation time of 0.4s. The discretized mesh was 2 hexahedral elements tall in the z-direction, and we only allowed the genetic algorithm to allocate contractile material on the xy-plane (30 elements by 10 elements), so along the z-direction we would not see varying materials. This was mainly done due to computational efficiency to minimize the mesh size. Codes used for modeling can be found here: https://github.com/srl-ethz/bilayered_optimization.

Both the simulation framework and the optimization algorithm are provided open-source. This will allow researchers to easily define search spaces and objective metrics for their own applications.

For the optimization we used the PAGMO package in C++, running the genetic algorithm "NSGA-II" to search over a binary mask corresponding to the muscle allocation of the

hexahedral mesh with a population size of 20 and distribution index for mutation of 20. The metric we use is the average distance norm of all vertices between the rest shape and the final contracted shape. But we first align the final shape with the initial shape through the Kabsch algorithm (48) to find an optimal rotation to match the zero-centered meshes. Asymmetric actuation patterns might cause nonzero net torques on the deformable body, which we do not want to consider, and can be removed with this alignment during metric computation.

Computational Analysis of Volumetric Bilayer Designs

*The same metric is used as described in the previous section; however, we now change the design space to only consider one muscle group, which we change in width and height. The grid search over the width and height of the muscle presented in **Fig. 3B** is applied to the same 30x10x2 voxel grid as for the evolutionary algorithm. To change the stiffness of the scaffold, we ran at a much finer resolution to search over more height ratios, i.e., how many cells in the y-direction are muscle compared to scaffold. For the stiffness sweep in **Fig. 3A** we ran the simulation with a 15x50x2 voxel grid for the bioactuator, staying with the same physical dimensions as before of 15 mm x 1 mm x 3.5 mm. The muscle is always full width." (Lines 483 – 543)*

Reviewer 1-6.4: It was good to talk about the variability in the designs as they did in page 10-11, as this is a large unaddressed limitation in the field. I'd encourage to make it even more systematic and provide more details about all of the different performances they obtained, sharing as many details as possible.

Answer 1-6.4: We thank the reviewer for this assessment of the importance to talk about variability in performance. To address this, we have provided the details of the different actuators in Table S2.

Reviewer 1-7: I genuinely enjoyed reading the paper and I think it has potential - it's well written and relevant. I appreciate the effort that went into combining computational modelling and experimental work. That said, I feel the focus should shift from proving that the design performs best to showing what can be learned from it. With that adjustment, the study could make a meaningful contribution to how computational approaches are applied in biohybrid systems.

Answer 1-7: We thank the reviewer for the positive ending, and hope that the changes have addressed the main concerns.
In summary, we have:

- Shifted the focus of the performance metrics from Fig. 1 to Fig. 4, and better clarified the purpose of the literature comparison, as can be seen in Figs. R1 and R2 in this document.
- Expanded upon the computational modeling by adding an extra figure and more computational modeling results, as seen in Figs. R3 and R4 in this document.
- Emphasized the application of computational approaches as the purpose of the paper by revising Fig. 1 (Fig. R1 in this document).
- Added additional detail on the computational framework to materials and methods.

Reviewer #1 (Remarks on code availability)

The code has a README file with instructions to reproduce. It seems to contain all the files necessary for reproducing the results. However, I haven't attempted to reproduce it now.

Answer: Thanks for checking this. We have updated the github repository (https://github.com/srl-ethz/bilayered_optimization) and checked for clarity.

Reviewer #3 (Remarks to the Author)

Reviewer 3-0: The paper "Co-Optimized Muscle Design for Large Dynamic Deformations in Versatile Biohybrid Robots" presents a computationally guided experimental pipeline for designing biohybrid robots. The authors' work is interesting, but the overall novelty appears limited. Machine learning and evolutionary algorithms have already been applied to the optimization of biohybrid robots (e.g., Blackiston et al., Sci. Robot. 6, eabf1571, 2021; Hugo Alcaraz-Herrera et al., https://doi.org/10.1007/978-3-031-87639-4_1; Zimmerman et al., Sci. Robot. 10, eadr6472, 2025). Furthermore, the additional elements of the manuscript (e.g., hydrogel choice, muscle maturation level) do not appear to introduce meaningful advancements beyond the current state of the art.

Answer 3-0: We thank the reviewer for their thoughtful assessment of our manuscript and for highlighting concerns regarding novelty relative to prior work employing evolutionary algorithms and machine learning in biohybrid robotics. We appreciate the opportunity to clarify the specific contribution of our study.

While previous works have applied optimization strategies to biohybrid systems, our primary contribution lies in the concurrent volumetric co-optimization of muscle and scaffold material distributions within a finite element framework*,* rather than optimization of external morphology or kinematics alone. Our approach does not rely on thin-film approximations or predefined bilayer assumptions but instead allows contractile and passive elements to be distributed voxel-by-voxel in a fully volumetric design space. This enables a broader and less constrained search space for actuator architectures. We demonstrate the approach on a relatively simple geometry to clearly illustrate the capabilities of the co-optimization framework before applying it to more complex biohybrid architectures. For future work, we anticipate that this framework will be useful for guiding the design of increasingly complex biohybrid constructs.

With respect to the hydrogel formulation and muscle maturation level, we agree that these components do not represent standalone material innovations. Our intention was not to introduce a new biomaterial platform, but rather to demonstrate a computationally informed design strategy using experimentally accessible and widely adopted materials. We have clarified this scope and tempered novelty claims where appropriate.

We address each of the reviewer's major points in detail below.

Additional major issues are outlined below:

Reviewer 3-1: In the Introduction, the authors state that "the optimization of centimeter-scale biohybrid actuators accounting for muscle and scaffold material behavior has not

yet been achieved." However, Zimmermann et al. have already demonstrated optimization of macroscopic, ray-like biohybrid devices. The prototypes presented in this paper appear only slightly larger, which does not substantiate a novelty claim in the authors' approach.

Answer 3-1: We thank the reviewer for highlighting the important work by Zimmermann et al. We agree with the reviewer that slightly larger prototypes of muscle construct would not constitute a sufficient novelty claim per se. However, through our manuscript we intended to communicate that the novelty would not lie in device scale, but in the *volumetric co-optimization* of both muscle tissue and scaffold material. We are proposing a method that specifically applies to systems where the muscle is "**volumetric"** (not just larger). The cardiac thin films developed by the Parker group consist of a monolayer of cells cultured on thin elastic substrates and are appropriately modeled using thin-film approximations such as Stoney's equation. In contrast, our system integrates a 3D muscle tissue layer with a hydrogel scaffold, forming a true volumetric bilayer architecture. Thin-film approximations are not valid for such tissue-based actuators. Therefore, we implemented our muscle and scaffold bioactuators into a finite element method that allows arbitrary elements to contract, enabling a more expressive search space of volumetric designs.

We believe that this clarification is important, also because, beyond the method novelty, the relevance of this work lies in addressing volumetric muscle architectures, an increasingly important target morphology not only in biohybrid robotics but also in tissue engineering, where achieving functional, scalable 3D tissue volume remains a critical objective for the field.

We understand that in the prior version of the manuscript, these points were not fairly or properly expressed and claimed for the correct understanding of the readers, and that clearer statements were needed to identify more exactly the novel contribution provided by this work, as well as the potential future areas of application and further implications.

Thus, to better differentiate our bilayer tissue geometry from the cardiac thin film work, we have used the terminology of "volumetric bilayer" and changed the paper title to "Co-Optimized Volumetric Muscle Design for Large Dynamic Deformations in Versatile Biohybrid Robots". We have edited the introduction for clarity in Lines 46, 62-63, 88-91, so that the purpose of the methodology and the scope of the novelty becomes better recognizable from the very beginning.

"Biohybrid robots can take a variety of forms, such as swimming biohybrids in the shape offish (4), rays (5–7), or jellyfish (8) fabricated from single cell layers (< 10 µm thickness) of cardiac cells combined with soft scaffold materials to propel themselves through fluid

using beating motions. Other designs use ring- or block-shaped skeletal muscle tissues (5 – 16 mm in length, 100 – 500 µm thickness) [...]" (Lines 42 – 46)

"Models developed for volumetric, tissue-like biohybrid muscle actuators must enable a bottom-up combination of constitutive materials to successfully probe the entire parameter space." (Lines 62-63)

"Therefore, the optimization of centimeter-scale biohybrid actuators accounting for volumetric muscle and scaffold material behavior has not yet been achieved.

In this work, we present a pipeline to co-optimize the volumetric muscle and scaffold elements of a biohybrid muscle-driven robot [...]" (Lines 88 – 91)

Reviewer 3-2: The algorithm converges on a bilayer design with an inner muscle layer and an outer scaffold layer that bends upon contraction. This result is not particularly surprising and could likely have been derived using simpler analytical models. Similarly, the observation that stiffer scaffolds require thicker muscle layers is intuitive and aligns with prior parametric studies conducted via FEM or analytical approaches. The identification of an optimal muscle volume fraction is potentially interesting, but the authors should justify why this insight could not have been obtained through existing modeling strategies.

Answer 3-2: We acknowledge the simplicity of the bilayered solution; however, we believe that simplicity alone does not diminish the validity of the co-optimization method showcased in the manuscript. An analytical solution could be found if assumptions are made on how many contractile muscle groups exist or how the structure deforms. We do not make such assumptions and keep the most general possible case for a bioactuator where contractile muscle elements are allocated within a scaffold structure. Our FEM and optimization implementation provides a simple, open-source framework for researchers to use, where the search space is general enough to be applied in any setting. Even when the optimal architecture appears simple, the ability to discover such solutions automatically from a large volumetric search space is essential for extending the approach to more complex geometries where analytical intuition is insufficient.

To strengthen this point of expressiveness, we adjusted the genetic algorithm (by proposing a different implementation where we increased the mutation rate) and ran it until convergence (as seen in Fig. 2, Fig. R3 in this document). The final shape is less intuitive now, with a tapered muscle towards the edges of the scaffold. We also added an analysis on adhesion effects in Fig. 3 (Fig. R4 in this document) and further supplementary results in Fig. S2, which ran the optimization that is allowed to remove

material as well, both showing the generality of our FEM approach. In Fig. S2 we see how bilayers form as thin as possible, which we further analyze in Fig. 3 to see how this ratio of muscle and scaffold changes with stiffness.

Please see the updated Figs. 2 and 3 copied below, referred to as Figs. R3 and R4 in this document.

Further, we have applied our computational pipeline to a pillar-based system, which is commonly used in the biohybrid robotics field. The results of this are see in Fig. 2 (Fig. R3 in this document) and described in lines 163 – 166, copied below.

"As a comparative case, we demonstrated the applicability of our algorithm to common biohybrid set-ups like a bioactuator around two pillars, maximizing for pillar deformation ***(Fig. 2B).*** *In this case, our algorithm converged to a muscle and scaffold distribution where passive elements were on the outside of the pillars, while the remainder of the structure is muscular." (Lines 163-166)*

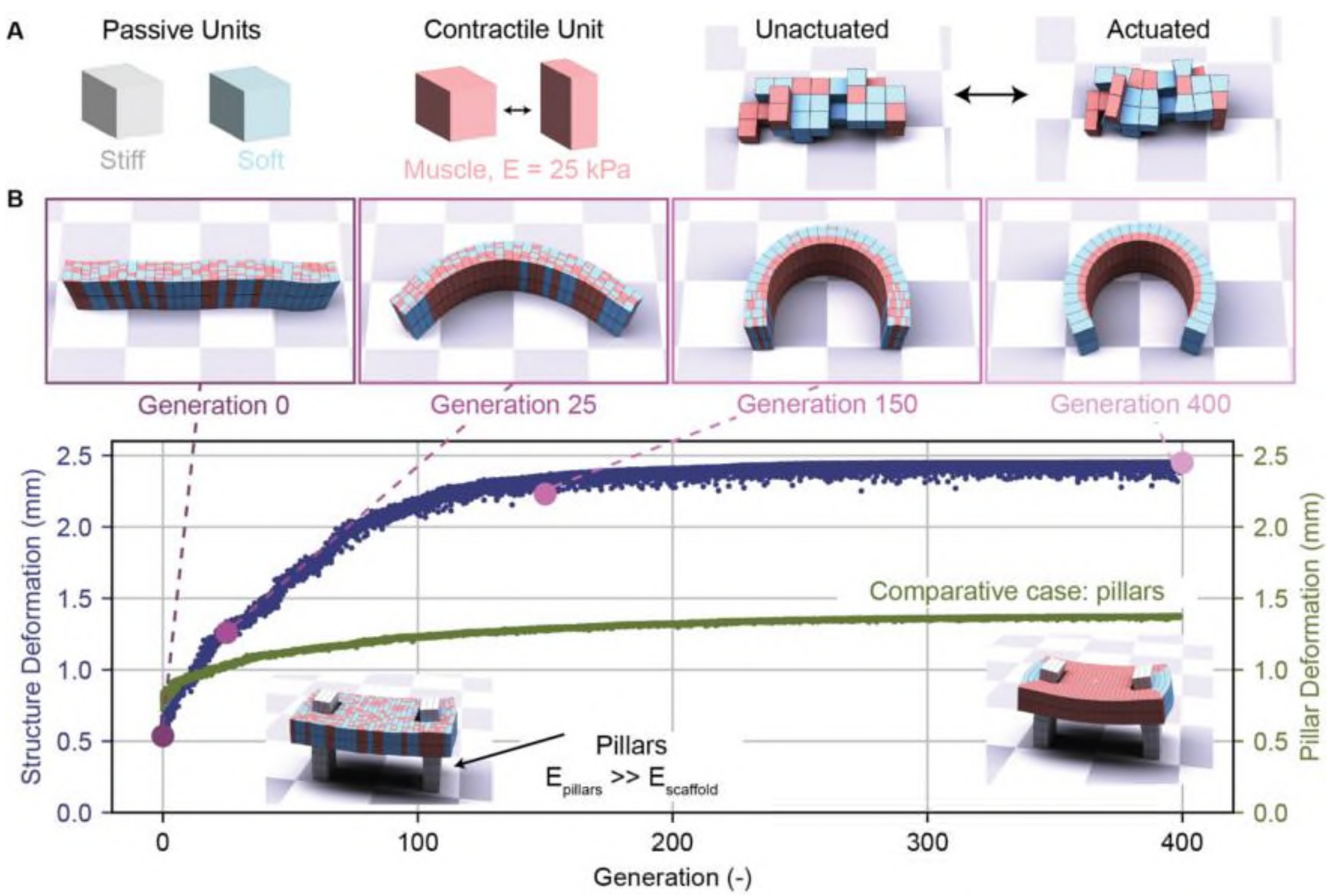

Figure R3 | Figure 2: Computational co-optimization of the volumetric muscle and scaffold elements of biohybrid actuators. (A) We defined biohybrid actuators in simulation as objects consisting of contractile and non-contractile materials. **(B)** We used an evolutionary algorithm to optimize for a geometry that generated the maximum range of movement (structure deformation). Examples of the actuator at

generation 0, 25, 150, and 400 are shown above the plot. As a comparative case, we used our method to optimize the bioactuator geometry for maximum deformation of pillars (y-axis on the right), a common biohybrid setup. The pillars were modeled as a stiff, passive material. Insets in the graph show the original randomized design and the final optimized bioactuator design.

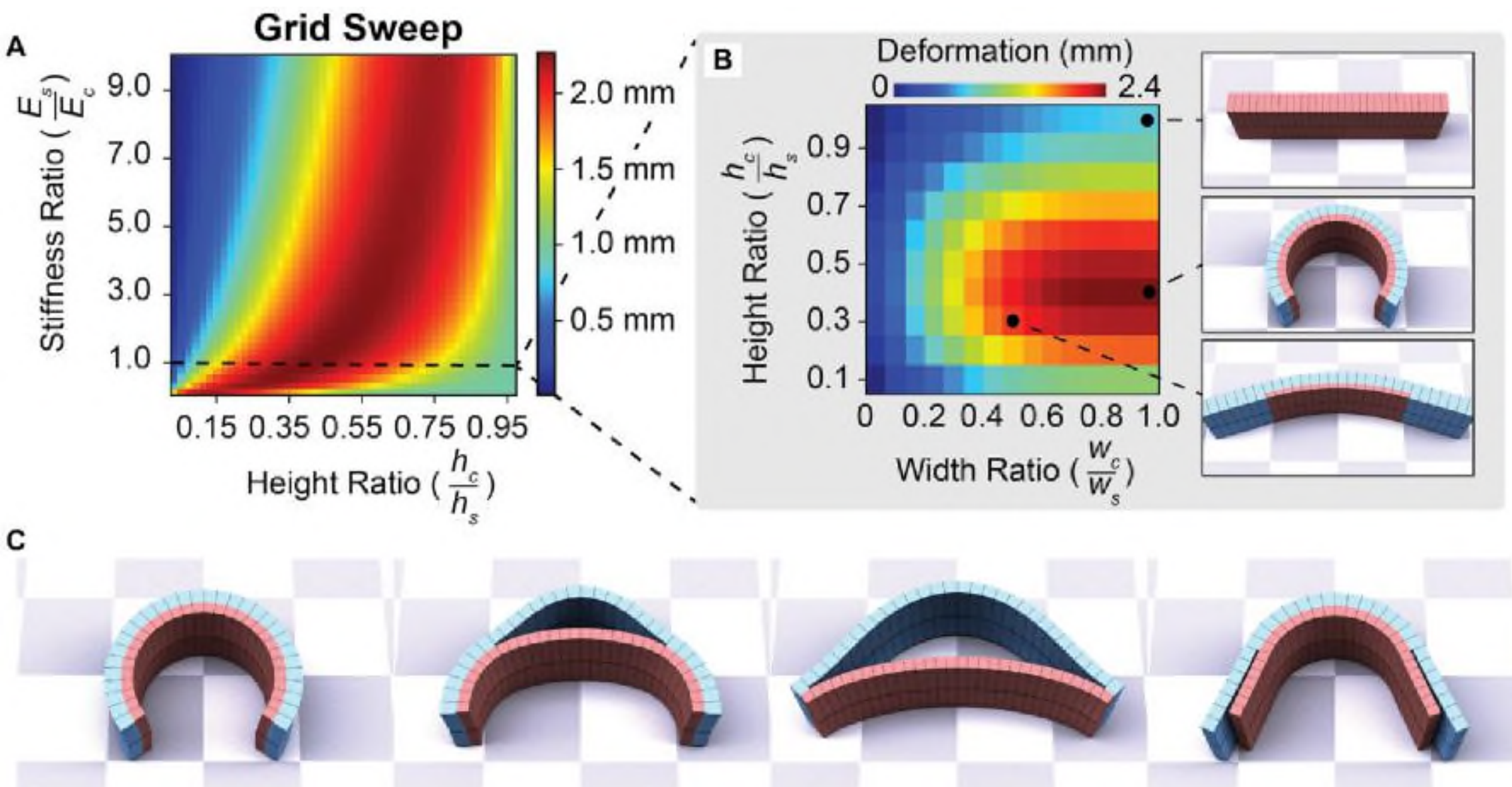

Figure R4 | Figure 3: Computational analysis on designing volumetric bilayer bioactuators. (A) Assuming a bilayered design, the distribution of muscle and scaffold of the bioactuator was refined by a grid optimization study to look at the effect of scaffold stiffness ratios and height ratios on deformation. **(B)** For a

was where the contractile layer comprises 40% of the height and spans 100% of the length. **(C)** Various structures where the muscle detaches from the scaffold. From left to right: A fully connected bilayer deforming 2.44mm, approximately 50% of the muscle is detached in the middle deforming 1.70mm, only the two ends are attached between muscle and scaffold deforming 1.46mm, and lastly roughly half the muscle is detached on the outer edges deforming 2.16mm.

fixed ratio of 1 between the scaffold and contractile unit stiffnesses, the optimal bioactuator geometry

Reviewer 3-3: The manuscript compares its results with extrapolations from prior work. This approach is problematic, as existing systems differ substantially in materials, culture conditions, and other critical parameters beyond the muscle–hydrogel volume ratio. To properly validate their hypothesis, the authors should demonstrate that their algorithm outperforms conventional computational or analytical models under consistent conditions, using the same hydrogel formulation, geometry, culture protocol, and other key variables.

Answer 3-3: We agree with the reviewer that the comparison between previous biohybrid works is challenging because there are no established and always-reported metrics for biohybrid systems. In our case, we wanted the comparison to previous work to show that the co-optimized design of scaffold and muscle in biohybrid systems is a useful approach.

To de-emphasize any unintended overclaims, we have removed the performance comparison from Fig. 1 and instead placed it into Fig. 4 and made corresponding changes in the text. Please see the corresponding Figs. copied below, with the captions omitted for conciseness. The captions can be found in the Figs. R1 and R2 copied below.

With regards to the comparison of the computational or analytical models under consistent conditions, our computational framework operates at the level of effective mechanical parameters (e.g., elastic modulus and contractile stress), rather than explicitly modeling specific hydrogel formulations or culture protocols. These experimental variables are therefore represented only through their mechanical equivalents in the model. As a result, meaningful benchmarking of the computational approach requires consistency in modeled parameters, rather than identical fabrication conditions. We specifically chose to use variables such as Young's modulus and contractile stress that are widely reported or can be extrapolated from images and videos with scale bars. For the comparison to other computational methods, we have expanded on our computational framework and genetic algorithm (see Figs. 2 – 3, in this document Figs. R3 and R4) to explain the advantages of our approach compared to computational approaches previously used in the literature.

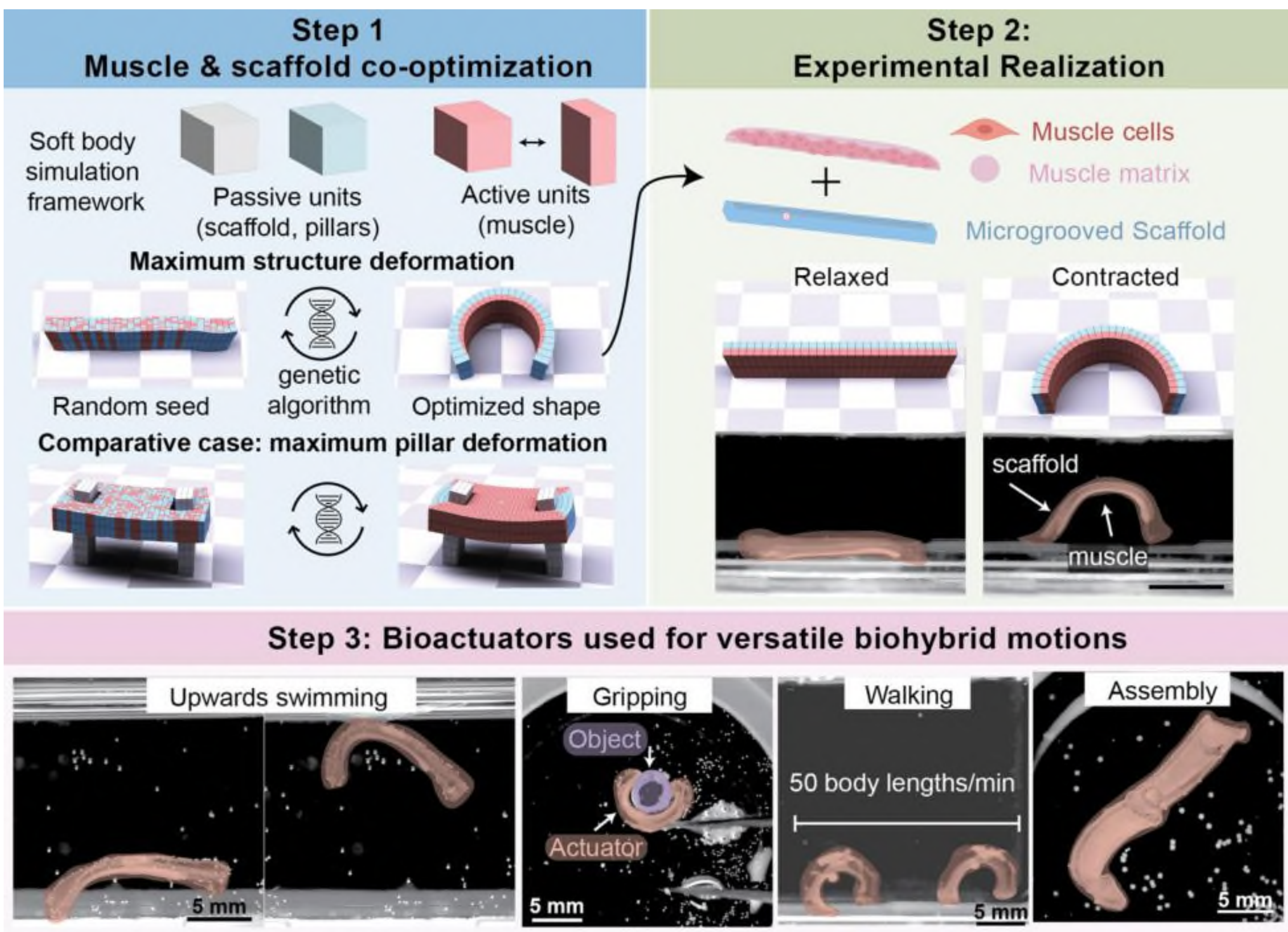

Figure R1 | Figure 1: **Computationally guided experimental pipeline for bioactuators with large dynamic deformations.** (caption omitted for conciseness, see first instance of figure above)

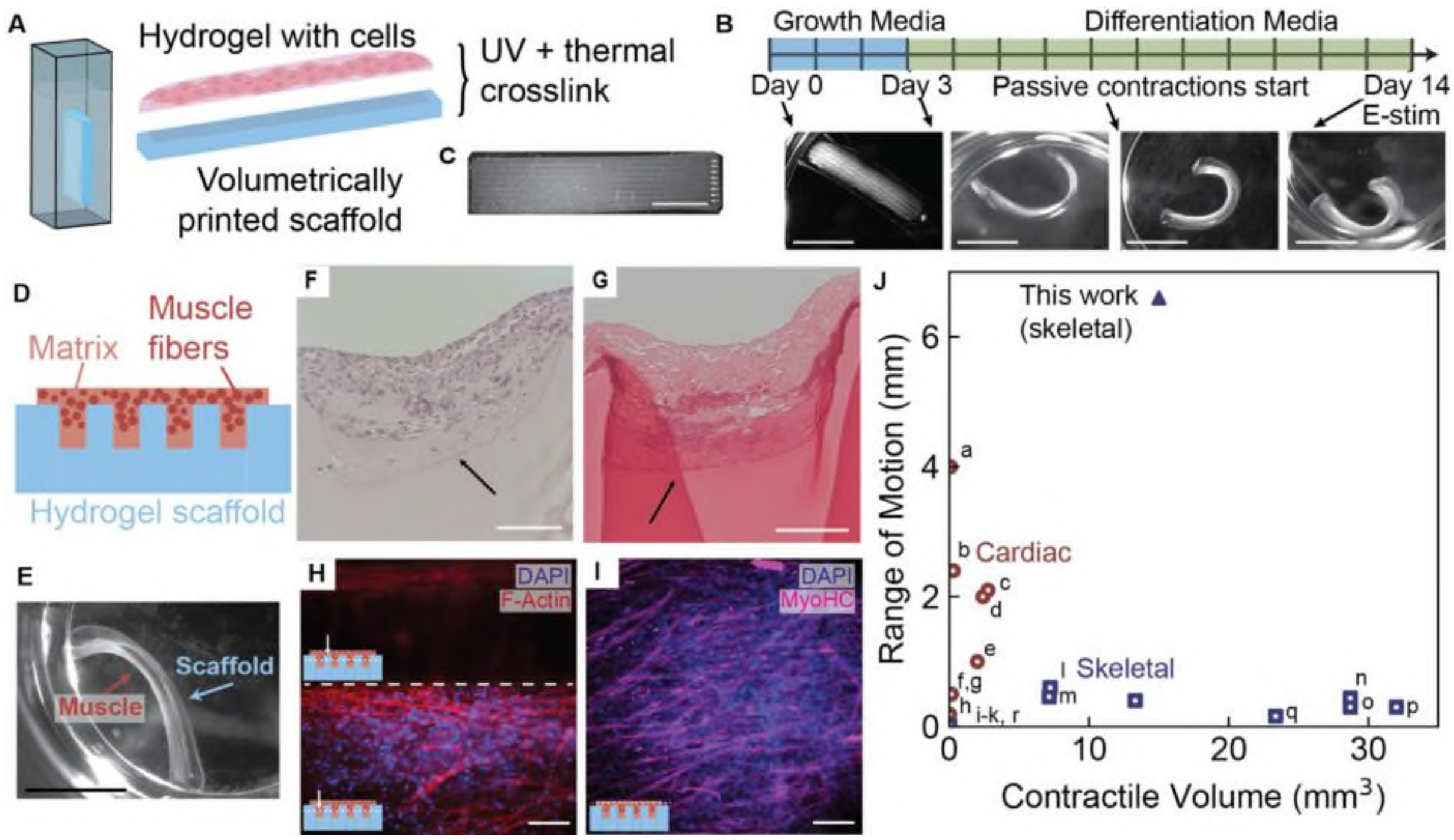

Figure R2 | Figure 4: **Volumetric bilayer bioactuator fabrication and characterization.** (caption omitted for conciseness, see first instance of figure above)

Reviewer 3-4: Scaffold stiffness affects not only mechanical deformation but also myotube differentiation and stability, which directly influence force generation. This important biological dimension is not addressed, limiting the validity of the conclusions.

Answer 3-4: We agree with the reviewer about the importance of the scaffold stiffness for myotube differentiation. We have included experimental results about the scaffold stiffness on the bilayered actuator formation in Fig. S1. In terms of the modeling results, we agree that accurately modeling muscle contraction is the next important step in the realm of modeling biohybrid muscle actuators; however, this topic is an open area of research and was not the focus of our work. In this work, we do not claim a matching force generation of the computational approach. Instead, we consider the material behavior in response to muscle contractions. The main topic of investigation is the interplay of active contractile units with passive elastic scaffold units, whereas much of the intricacies of how force is generated within the muscle units is left for future work. Thus, to emphasize this point and to clearly define the limitations of our current work, we have added lines 439 – 443 in the conclusion, copied below.

"Advantageously, our current framework is customizable and could readily integrate future computational models of muscle function and maturation. Incorporating such models would strengthen the conclusions drawn from our framework, for example, by enabling a more mechanistic investigation of how scaffold stiffness influences bioactuator functionality through the interplay between scaffold material properties and muscle tissue maturation." (Lines 439 – 443)

Reviewer 3-5: Key factors such as the hydrogel-tissue interface and the degree of muscle maturation are not incorporated into the algorithm. As these strongly impact device behavior, the omission raises concerns about the reliability and predictive power of the proposed framework.

Answer 3-5: With regards to the hydrogel-tissue interface, we assumed for the purposes of the model that the interface was continuous, which was supported by our experimental observations of the tissue-scaffold interface (Fig. 4F, 4G, S8). We have added computational results in Fig. 3C (Fig. R4 in this document) that demonstrate the importance of a stable and continuous hydrogel-tissue interface on the bioactuator performance. We show that the range of motion is negatively impacted if the muscle layer is not continuously integrated with the scaffold, as described in lines 197 – 200.

"Lastly, we investigated the influence of adhesion between muscle and scaffold ***(Fig. 3C)****. We removed connective tissue between the two and found that full adhesion results in the largest range of motion, where especially the central area matters and the muscle's outer edges have a smaller impact on the final deformation." (Lines 197 – 200)*

For the muscle maturation, the reviewer is correct in identifying that it is not incorporated into the algorithm, because the development of computational models for the maturation process is an open field of research. We proved the validity of our computational framework by using it to inform the bioactuator design and then showing that the computationally informed bioactuator design performed as expected compared to the relevant examples in literature. However, we agree with the reviewer that bridging the sim-to-real gap of biohybrid muscle actuation is a crucially needed step to take in future work, as explicitly stated in lines 434 – 443.

"Further, the simulation of a single actuation was fast enough to use a genetic algorithm to evolve bioactuators for range of motion and optimize for the distribution and material properties of muscle and scaffold layers. While the computational model informed the bioactuator design, more accurate modeling of muscle biomechanics and environmental interactions is necessary to enable sim-to-real fidelity. Advantageously, our current

framework is customizable and could readily integrate future computational models of muscle function and maturation. Incorporating such models would strengthen the conclusions drawn from our framework, for example, by enabling a more mechanistic investigation of how scaffold stiffness influences bioactuator functionality through the interplay between scaffold material properties and muscle tissue maturation." (Lines 434 – 443)

Reviewer 3-6: The biological data presented are relatively weak. The immunofluorescence images in Fig. 3 do not show well-aligned fibers or highly matured tissue, and overall myogenic differentiation appears poor compared with numerous existing studies.

Answer 3-6: In agreement with the suggestion of the reviewer, we have provided deeper considerations of the biological status of the muscle tissue. In particular, we have now added data concerning the crucial maturation markers, such as the fusion index and MyoHC+ myofiber diameter (lines 281-282 and in methods).

"As markers of maturation, we measured an average myofiber thickness of 14.4 ± 5.5 µm and a fusion index of 75 ± 11 %." (Lines 281 – 282)

Interestingly, we calculated the fusion index to be 75%, which is in line with other reported values of mature myofibers. However, the MyoHC+ myofiber thickness is 14.4 µm, which is relatively low for mature myofibers when compared to the literature. Despite the varied maturation markers, the constructs were functional, as demonstrated by active contractions observed, recorded, and analyzed in Figs. 5-8 and supplementary videos S1 – S12. Therefore, we conclude that to further improve the bio-actuator functionality, future work should focus on increasing the degree of maturation by optimizing culture conditions and topographical cues. This has been further clarified in the discussion in lines 465 – 469.

"The maturation markers of the final bioactuators were varied: the fusion index was in line with mature engineered muscle constructs (40, 41), but the MyoHC+ myofiber diameter was lower than expected for mature muscle (42). Therefore, future work to optimize culture conditions and/or topographical cues provided by the scaffold would aid in improving the differentiation process and could improve the bioactuator performance (39, 43)." (Lines 465 – 469)

Relevant references used in the text excerpt above:

39. S. Rangarajan, L. Madden, N. Bursac, Use of Flow, Electrical, and Mechanical Stimulation to Promote Engineering of Striated Muscles. *Ann Biomed Eng* **42**, 1391–1405 (2014).

40. M. Filippi, A. Balciunaite, A. Georgopoulou, P. Paniagua, F. Drescher, M. Nie, S. Takeuchi, F. Clemens, R. K. Katzschmann, Sensor-Embedded Muscle for Closed-Loop Controllable Actuation in Proprioceptive Biohybrid Robots. *Advanced Intelligent Systems* **n/a**, 2400413.

41. M. Filippi, O. Yasa, J. Giachino, R. Graf, A. Balciunaite, L. Stefani, R. K. Katzschmann, Perfusable Biohybrid Designs for Bioprinted Skeletal Muscle Tissue. *Advanced Healthcare Materials* **12** (2023).

42. C. Vesga-Castro, J. Aldazabal, A. Vallejo-Illarramendi, J. Paredes, Contractile force assessment methods for in vitro skeletal muscle tissues. *eLife* **11**, e77204 (2022).

43. R. Raman, C. Cvetkovic, R. Bashir, A modular approach to the design, fabrication, and characterization of muscle-powered biological machines. *Nat Protoc* **12**, 519–533 (2017).

Reviewer 3-7: The hydrogel formulation (gelatin methacrylate + polyethylene glycol diacrylate) is neither new nor optimal for supporting muscle differentiation and functionality.

Answer 3-7: We thank the reviewer for this observation and clarify that the muscle cell-containing hydrogel is composed of 5wt% GelMA, 0.1 wt% LAP, and 66% Matrigel (thus, a relatively standard polymer mix that is functional for the chosen biofabrication approach). In contrast, the hydrogel formulation of gelatin methacrylate + polyethylene glycol diacrylate is only used to fabricate the scaffold, which does not contain any cells inside.

As proof of cell viability in the cell-containing hydrogel, we have included Live/Dead stainings in Fig. S5. To help avoid any future reader confusion, we have now specified the contents of the cell-containing hydrogel in the main text, in line 237, whereas previously these details were contained only in the methods section.

"A skeletal muscle tissue layer of dimensions 12 mm x 3 mm x 0.5 mm was formed on top of the scaffold by depositing a mixture of 5 wt% GelMA, 0.1 wt% LAP, and 66% Matrigel with 5 x 10^6 C2C12 cells onto the scaffold and then following a growth and differentiation protocol to form contractile skeletal muscle tissue ***(Fig. 4A, 4B)****." (Lines 236 – 239)*

Rebuttal to Reviewer Comments – Nature Communications submission

‘Co-Optimized Volumetric Muscle Design for Large Dynamic Deformations in Versatile Biohybrid Robots’ (Manuscript ID NCOMMS-25-73697A)

Reviewer #1 (Remarks to the Author):

Thank you to the authors for the comprehensive respond to the comments and revised manuscripts. I believe most of the comments have been addressed and I also appreciate the efforts to improve the computational and methodological details. I will only comment on the design aspects, which is the main contribution of the paper.

1.1 The addition of some variation to their initial genetic algorithm definitely makes the results more meaningful. It’s indeed quite interesting that optimised design with a tapered muscle towards the edges. I do have to say I did not understand Figure S2 and what it’s meant to show. The authors talk about the designs forming bilayers, but all I see is some weird designs. Probably some further explanation in the caption would be useful for those slow like me.

Thank you for pointing out this lack of clarity, we have improved on the figure caption. In the rest of paper we assume the only choice of the optimization algorithm is to set a cell as either active (muscle) or passive (scaffold). In Figure S2, we also allow the cell to be marked empty, which means the mesh becomes disconnected at that location. In the figure you can see how some cells are missing, and for the first shape, it specifically created disconnected 2-layer thin sheets (1 layer blue scaffold and 1 layer red muscle), and multiple of them, so they created this “M”-shape since it’s three bilayers attached at the edges. In the shape on the right, for a lower resolution mesh, it again creates these 2-layer structures, proving heuristically the designs try to evolve into structures with one layer active and one layer passive cells. I hope this clears up any confusion the Reviewer had.

“***Figure S2****: Unconstrained topology optimization for muscle allocation. Every cell is marked as either empty, passive (blue scaffold), or active (red muscle).* ***(A)*** *A constant depth (z-axis) search over a 10x10x1 grid where self-collision is allowed. We see a natural formation of bilayers with empty space between the active-passive layer combination. For 10 layers along the y-axis, the optimal amount of bilayers is 3, which the genetic algorithm finds.* ***(B)*** *For a 3D search over a 9x3x3 voxel grid, we find the algorithm converging to*

two distinct bilayers. These experiments prove that heuristically the designs try to evolve into structures with one layer of active and one layer of passive cells."

1.2 It's also quite interesting to see how the results change when the simulation allows to remove material as well. However, I think this needs now some further explanation, since I wasn't able to locate it in the text. What do you mean exactly with "allowed to remove material"? How does it work?

Answer: Thank you for raising this — we have expanded the explanation in the text. As described above for Figure S2, the optimization normally assigns each cell one of two states: active (muscle) or passive (scaffold). When we "allow the algorithm to remove material," we add a third option: a cell can also be marked empty. An empty cell is removed from the simulation mesh entirely, so the structure becomes disconnected at that location rather than being filled with either muscle or scaffold. We have clarified this mechanism in the relevant section of the text and in the Figure S2 caption.

1.3 Likewise for the adhesion effects – those are quite interesting and potentially useful, but it's not clear from lines 197-200 in page 9 how does that work exactly in the simulation. Can different levels of adhesion be modelled in your simulation? What values did you choose? What does it mean to "remove connective tissue"?

Thanks for the question. We have clarified this in the text by writing "*We modeled removing connective tissue between the two layers by using empty voxels between muscle and scaffold layers to model a separated tissue and scaffold.*" (lines 200-202) We can change the amount of separation of tissue along the length of the structure by changing the number of cells we mark empty.

1.4 I find showcasing this potential one of the strengths of the papers, and especially of this revision. The readers would benefit from understanding what's possible in different settings, rather than just focusing too much on a very specific example (such as in the previous version). I'd recommend the authors to streamline this initial part, and show systematically all of these potential case studies and variations. This would diminish the concerns of the case study being "too simplistic" and highlight the potential of the work. After having done that, they can focus on one particular case study for the rest of the experiments as it stands already.

We thank the Reviewer for this positive feedback and for highlighting the potential of the work. We agree that showcasing the breadth of possible designs and settings is a strength of the revision. To make the flexibility of the modeling framework more explicit, we have added the following sentence to the text: "*The voxel-based modeling approach enabled geometric freedom and unrestricted material selection, allowing for a variety of*

bioactuator designs to be explored within the simulation framework, as well as modeling aspects such as empty space between muscle and scaffold." (Lines 141 – 144)

The different designs and variations are then systematically showcased in the figures that follow.

1.5 Regarding that, I would add an extra challenge to the authors – which is not compulsory but if it's not too time-consuming it might be worth it. I notice that the simulation with the pillar setup is quite interesting, and manages to add interesting things like having "soft tissue" behind the pillars instead of contractile units (as those are not doing much). But I also notice that is not realistic in the sense that we can never have (at least with current capabilities) a central part full of muscle tissue between both pillars – it needs to be some sort of ring, or the cells would die. I wonder if the authors can add some constraints to their model to, for instance, limit the thickness of the contractile units, to e.g. 200-300 um of thickness. It would be interesting to see if adding these real-life constraints would make their converged designs for realistic (e.g., converging into rings), which would strengthen the potential of their approach. While I understand the authors wanted to emphasize the free search in a not-too-constrained search space, this is already demonstrated by the simpler geometries and then they can proceed to add realistic constraints and the search can still be unconstrained within those limitations.

Answer: We thank the Reviewer for this thoughtful suggestion, and we agree it is an interesting direction. The Reviewer is correct that the diffusion limit in tissues makes a fully muscle-filled core between two pillars biologically unrealistic, and that introducing constraints such as a maximum contractile-layer thickness would be a natural way to push the converged designs toward more physically realizable geometries, potentially recovering ring-like solutions.

We would note that our framework is, by design, well suited to exactly this kind of extension: because the search operates on a free voxel allocation with adjustable penalties, thickness limits or other fabrication constraints can be incorporated directly into the optimization without changing the underlying method. In that sense, the suggestion reinforces rather the generality we aim to demonstrate.

That said, we feel a full constrained-optimization study of this kind — implementing the diffusion-informed thickness limits, re-running convergence, and validating the resulting geometries — goes somewhat beyond the scope of the present manuscript, whose focus is on establishing the co-optimization pipeline and its experimental realization. We would prefer to treat it as a clearly motivated next step rather than fold a partial version into this paper. We have therefore noted in the discussion that incorporating biologically motivated

fabrication constraints (such as diffusion-limited tissue thickness) is a promising avenue for future work.

"*Advantageously, our co-optimization framework can readily accommodate biologically-motivated constraints such as diffusion-limited tissue thickness, and we identify this as a promising next step to inform the design of larger-scale, perfusable tissues.*" (Lines 487 – 489)

1.6 In sum, as the authors are already showing different variations or applications of their approach, going from the simple bilayer structure to different adhesions, to removing material and the pillar structure, I think the paper would benefit for a more systematic arrangement of those cases that can show the reader what's the potential of their approach. After that, they can focus on one type of design and prove it experimentally. This is just my suggestion – I think it's clear as it is right now (except for those couple of places where I indicated details were missing), but I think it could strengthen the contribution if it's framed like that.

Answer: We thank the Reviewer for this constructive suggestion and for noting that the paper is clear as it stands. We agree that systematically laying out the range of cases our approach can address before focusing on one design for experimental validation is the right way to convey its potential. This was our goal when we reorganize Figure 1 in the previous revision to present this progression. We considered moving the adhesion aspects into Figure 1 but will keep them in Fig. 3, so as not to crowd the first figure. We have therefore not made further structural changes, but we are grateful for the Reviewer's suggestions in improving the paper.

Reviewer #1 (Remarks on code availability):

Did not review it this second time.

Reviewer #3 (Remarks to the Author):

I sincerely appreciate the efforts made by the authors to address the reviewers' comments and to improve the manuscript. However, my main concerns remain.

3.1 I continue to find the work interesting; however, its overall novelty appears limited. As previously noted, machine learning and evolutionary algorithms have already been applied to the optimization of biohybrid robots. The novelty highlighted by the authors, namely, the concurrent volumetric co-optimization of muscle and scaffold material distributions, does not, in my view, constitute a decisive advancement in the field. Moreover, the authors defer the development of "complex architectures," and thus more functional biohybrid robots, to future work. Overall, the level of novelty presented does not appear sufficient for Nature Communications.

We thank the Reviewer for continuing to find the work interesting, and we welcome the chance to state our contribution more sharply. We believe the novelty does not rest on the use of evolutionary optimization per se, but on what our integrated pipeline accomplishes and the substantial, experimentally validated performance gains it delivers. We would like to clarify three points.**1. Our contribution is distinct from prior optimization of biohybrid robots in both scope and physical regime.** We agree that machine learning and evolutionary methods have been applied to biohybrid design, and we cite the two prior demonstrations directly. However, both differ fundamentally from our work:

- Kriegman et al. used a voxel-based approach to optimize **microscale** reconfigurable organisms (< 1 mm in all dimensions), without engineered scaffold materials.
- Zimmerman et al. used a **purely geometric, biologically agnostic** model that did not consider the shape or material properties of the muscle layer, and was restricted to thin, single-cell-layer (~10 μm) contractile films a few millimeters in their longest dimension.

Neither accounts for the coupled, volumetric behavior of muscle tissue and scaffold material at the centimeter scale. Our pipeline is, to our knowledge, the first to co-optimize the volumetric material distribution of both the living and non-living components of a centimeter-scale biohybrid actuator. This required a simulation framework with Neohookean constitutive behavior and implicit time integration that remains stable and accurate under the large deformations our actuators undergo. Based on these observed difference, our contribution qualifies as a conceptual advance rather than a scaling-up of existing approaches: previous studies optimized either microscale biological constructs or simplified thin-film systems, whereas our framework enables the **integrated design of**

volumetric biohybrid actuators whose behavior emerges from the coupled mechanics of living tissue and engineered scaffold materials

2. The novelty is the unified modeling–optimization–fabrication loop, validated by substantial experimental results. The point of our work is not optimization in the abstract, but that computationally derived designs were realized experimentally and produced large, measurable gains. Concretely, our co-optimized bioactuators achieved up to an eleven-fold increase in range of motion relative to previously reported designs of comparable contractile volume, a comparison drawn from previous biohybrid literature (Table S1). The same platform produced robots that jump, swim with frequency-tunable buoyancy control, walk (at a normalized speed that outperforms all previously reported biohybrid walkers, Table S5), and grip and translocate macroscale objects. We suggest that demonstrating a single design framework that translates into this breadth of validated, state-of-the-art function is itself the impactful result, beyond the optimization machinery that enabled it. Thus, also from this angle, the novelty of this work lies in a fully integrated pipeline leading to reliable experimental realization of computationally designed biohybrid systems with large, validated performance gains across multiple robotic behaviors.

3. We do demonstrate complex architecture and scalability; what we defer is a narrower, well-defined extension. We would like to clarify that the manuscript goes beyond outlining future directions: we do present steps toward more complex and functional biohybrid robots, not only a promise of future work. In Figure 8, we show (i) modular assembly of matured units into multi-unit actuators with increased deformation, and (ii) a dual-leg actuator fabricated as a single construct, in which microgroove-directed alignment produces independent, perpendicular contraction of each leg. What we defer is specifically the computational optimization of motion profiles that incorporate environmental interaction, which is a targeted next step, not the absence of complex architecture in the present work.

In summary, we believe the combination of a new co-optimization capability for volumetric muscle–scaffold systems, a fabrication method enabling continuous force transmission across a stable biohybrid interface, and the resulting state-of-the-art, multi-modal robot performance constitutes a substantial advance for the field. We would be glad to make these contributions more prominent in the manuscript text if the Editor or Reviewer feels they are currently understated.

3.2 The authors have reformulated their claims by emphasizing the concept of a "volumetric bilayer." While it is true that, from both design pipeline and fabrication perspectives, this approach differs from other bilayered scaffolds reported in the literature, it nevertheless remains a bilayer system. As such, it is far from the "volumetric, tissue-like biohybrid muscle actuators" claimed in the manuscript. A truly volumetric, tissue-like

structure would exhibit features more closely resembling native tissue, including complex architecture, functional interfaces, and potentially vasculature. In contrast, the proposed design consists of a bilayer structure with an inner muscle layer and an outer scaffold layer that bends upon contraction. This suggests that the computation-informed design approach, although interesting, does not yet lead to outcomes that are sufficiently novel or impactful for the fields of biohybrid devices or skeletal muscle tissue engineering. The additional designs presented by the authors do not resolve this limitation.

Answer: We thank the Reviewer for appreciating our concept of a "volumetric bilayer" which highlights that our system differs, in both design and fabrication, from the literature. We are fully in agreement that our engineered skeletal muscle tissue layer is not replicating a biological tissue completely. In order to ensure that we are as clear as possible on this note with readers, we have removed the term "tissue-like" from line 62 in the introduction, and added a clarifying sentence stating that we use the words "skeletal muscle tissue layer" to refer to the engineered, contractile, muscle layer on the bioactuator. While the skeletal muscle tissue layer is in line with 3D biohybrid muscle tissues, it is not completely representative of a physiological tissue that would require multiple cell types, perfusability, etc.

"A skeletal muscle tissue layer of dimensions 12 mm x 3 mm x 0.3 mm was formed on top of the scaffold by depositing a mixture of 5 wt% GelMA, 0.1 wt% LAP, and 66% Matrigel with 5 x 10^6 C2C12 cells onto the scaffold and then following a growth and differentiation protocol to form contractile biohybrid muscle ***(Fig. 4A, 4B)****. We use the term "skeletal muscle tissue layer" throughout this manuscript to refer to the engineered, contractile layer of the bioactuator; while this layer is consistent with 3D biohybrid muscle tissues, it is not fully representative of a physiological tissue, which would require multiple cell types, perfusability, and other features." (Lines 240 – 247)*

In terms of biohybrid literature, the state-of-the-art biohybrid tissue actuators are predominantly small-scale, mold-cast hydrogel constructs, typically rings (e.g. Raman et al., 2017 Nat. Prot.; Kim et al., 2023 Sci. Rob.), sheets (e.g. Morimoto et al., 2018, Sci. Rob.), or rods (e.g. Ren et al., 2025, Sci. Rob.) with thickness below 1 mm. None of these incorporate perfusion, complex internal architecture, or engineered functional interfaces. Against this benchmark, we believe our system represents a meaningful step forward along several of the axes the Reviewer raises:

- **Tissue-like dimensions.** We agree with the Reviewer that our method differs in both design and fabrication from bilayers reported in the literature. As demonstrated by histology in Figure S8 and stereomicroscope images of the constructs (Figs. 4, 5, 7, 8), the muscle layer reaches a thickness of 200 - 300 µm, which is approximately 20-fold greater than a single cell layer (~10 µm). This

places our construct well beyond the regime of thin single-cell-layered actuators and into a 3D tissue volume.

- **Functional interface.** The muscle–scaffold interface is not a passive contact but a mechanically functional one, exhibiting consistent and reproducible adhesion throughout maturation and actuation. This is, to our knowledge, not a feature of the molded hydrogel actuators referenced above.
- **Architectural complexity.** Our scaffold imposes a grooved topography that directs cell alignment and shapes the resulting tissue organization. While we agree this is not "complex" in the sense of native musculature with vasculature and connective hierarchies, it constitutes a designed architectural feature absent from comparable biohybrid systems.
- **Perfusion.** We fully concede that our construct does not incorporate perfusion. However, this limitation is shared by essentially all existing biohybrid muscle actuators, including the ring, sheet, and rod geometries that define the current state of the art. Engineering perfused biohybrid actuators remains an open challenge for the field as a whole, not only for our approach.

Finally, regarding the novelty and impact of the computation-informed design approach: the purpose of the modeling framework is precisely to optimize biohybrid actuator design within the present technological constraints of the field (including the absence of vasculature). We therefore believe it offers a useful contribution by enabling rational, predictive design choices rather than by claiming to resolve all open challenges of skeletal muscle tissue engineering at once.

3.3 I appreciate the authors' efforts to moderate potential overstatements; however, I remain of the opinion that, to properly validate their central hypothesis, namely, the value of computation-informed design, they should demonstrate that their algorithm outperforms conventional computational or analytical approaches under comparable conditions. The absence of such validation weakens the overall claims.

Answer: We thank the Reviewer for this comment. We believe that we have addressed this point in the previous revision and reproduce a similar response below. In addition, we want to clarify what we do and do not claim, as we suspect this might be the source of the confusion.

We do not claim that our simulation framework (SORS) or the genetic algorithm we use outperforms other methods. Neither component is novel, and several alternative simulators and optimizers could in principle have reached similar conclusions. We chose SORS because it is a stable, open-source soft-body framework developed in our own lab, which made it a natural and practical basis for this work. The algorithm was never our claimed contribution. Our contribution is what the pipeline enables: the co-optimization of

volumetric muscle and scaffold elements, the experimental realization and validation of the resulting designs, and the open-source code we provide alongside them.

For this reason, we believe a benchmark here would not be informative. Because neither the simulator nor the optimizer is new, comparing them against other methods would, at best, show that any reasonable method performs adequately, which does not bear on the central hypothesis of the paper. A methods comparison would require surveying the field's various modeling and optimization approaches and re-running them on our problem, which would constitute a separate methodological study. What matters here is the end result and its experimental validation, not the choice of implementation.

The two prior optimization studies operate in fundamentally different regimes: microscale organisms without engineered scaffolds (Kriegman et al.) and biologically agnostic, single-cell-layer thin films (Zimmerman et al.). As stated in the manuscript:

"To date, only two studies have demonstrated optimization of biohybrid designs. Kriegman et al. used a voxel-based approach to optimize microscale reconfigurable organisms (< 1 mm in all dimensions) (24). Zimmerman et al. showed a purely geometric, biologically agnostic model to optimize contractile thin films in fluid that did not consider the shape or properties of the muscle layer (26). The contractile films were only a few millimeters in the longest dimension and the active layer was a thin, single cell layer of approximately 10 μm. Therefore, the optimization of centimeter-scale biohybrid actuators accounting for volumetric muscle and scaffold material behavior has not yet been achieved." (Lines 82–89)

If we have nonetheless misunderstood the Reviewer's intent, we would be grateful for clarification on two points so that we can respond concretely:

First, which "algorithm" should be benchmarked: the modeling approach (SORS), or the optimization (the genetic algorithm)? Neither is new nor reported for the first time in this paper, so it is unclear what a comparison of either would establish about our actual contribution.

Second, what is meant by "comparable conditions": should the comparison hold the computational parameters constant (modulus, contractile stress, search space), or the experimental conditions (hydrogel formulation, culture protocol)?

Absent a concrete comparator, we maintain that the value of the approach is substantiated by the novelty of the co-optimization itself, its experimental realization, and the open-source pipeline we provide, rather than by a benchmark against methods built on different assumptions and intended for different use cases.

3.4 I fully acknowledge that modeling muscle contraction is challenging. Nevertheless, omitting this aspect limits the generalizability of the proposed approach. Furthermore, modeling only the material response to muscle contraction could have been achieved using simpler methods, as noted in previous comments.

Answer: We thank the Reviewer for this comment, and we want to be clear at the outset: we fully agree that incorporating a biological muscle model is the next important step for our framework. Our discussion explicitly identifies it as a priority, noting that more accurate modeling of muscle biomechanics and environmental interactions is necessary for sim-to-real fidelity, and that our framework is deliberately built to be customizable so that future muscle-function and maturation models can be integrated directly into it.

Where we would respectfully push back is on the suggestion that omitting an explicit contraction model limits the generalizability of the approach, or that simpler methods would have sufficed. We addressed both points in detail in our previous response, and we are not certain which part of that reasoning the Reviewer finds unconvincing. To make the discussion as productive as possible, we reproduce our earlier argument below and would be grateful if the Reviewer could indicate specifically where they disagree, so that we can respond precisely rather than in general terms.

On generalizability and the sufficiency of simpler methods (from our previous response):

An analytical solution could be found if assumptions are made about how many contractile muscle groups exist or how the structure deforms. We deliberately make no such assumptions, keeping the most general possible case: contractile muscle elements freely allocated within a scaffold structure. Our FEM and optimization implementation provides a simple, open-source framework in which the search space is general enough to apply in any setting. Even when the optimal architecture appears simple in hindsight, the ability to discover such solutions automatically from a large volumetric search space is essential for extending the approach to more complex geometries where analytical intuition is insufficient.

To strengthen this point regarding the expressiveness of the method, we revised the genetic algorithm (using a different implementation with an increased mutation rate) and ran it to convergence (Fig. 2). The resulting shape is now less intuitive, with a muscle layer that tapers toward the edges of the scaffold — a solution unlikely to be reached by assumption. We additionally added an analysis of adhesion effects (Fig. 3) and further supplementary results (Fig. S2) in which the optimization is also permitted to remove material; both demonstrate the generality of our FEM approach. In Fig. S2, bilayers form

as thin as possible, which we then analyze in Fig. 3 to show how the muscle-to-scaffold ratio shifts with stiffness.

Finally, to demonstrate that the framework is not specific to one geometry, we applied the same pipeline to a pillar-based system, a configuration widely used in the biohybrid robotics field. As described in the manuscript (lines 166–169):

"As a comparative case, we demonstrated the applicability of our algorithm to common biohybrid set-ups like a bioactuator around two pillars, maximizing for pillar deformation (Fig. 2B). In this case, our algorithm converged to a muscle and scaffold distribution where passive elements were on the outside of the pillars, while the remainder of the structure is muscular."

Taken together, these results — a general, assumption-free search space, material-removal and adhesion analyses, and successful application to a distinct pillar-based geometry — are intended to show that the validity and generality of the method do not depend on an explicit contraction model. We therefore maintain that the approach is both valid and generalizable in its present form, while agreeing that a biological muscle model is the natural and important next extension.

We hope this clarifies our position, and we would welcome the Reviewer's specific feedback on which element of this argument they find insufficient, so that we can address it directly.

3.5 I appreciate the additional biological analyses performed by the authors; however, these remain relatively limited and point to a muscle structure that is not yet well matured. Although MyoHC+ myofibers are now presented, the fibers do not appear well aligned and instead show a largely random orientation, nor do they indicate a high degree of maturation. Appropriate controls should be included to confirm the specificity of the MyoHC staining. In addition, clearer images, and a sufficient number of them, should be provided to distinguish nuclei incorporated within myotubes from those that are not, thereby supporting the fusion index calculation, which cannot be reliably assessed from the current data. Further analyses would also be beneficial, such as imaging of sarcomeric organization and mRNA-based assays.

Answer: We thank the Reviewer for these comments. We address each point below.

MyoHC$^+$ myofibers and maturation. MyoHC$^+$ myofibers were presented in the original submission [originally Fig. 3], and we apologize if this was not sufficiently prominent; we have made these data clearer in the revised manuscript [now Fig. 4]. We agree that the structure represents an early stage of maturation and that fiber orientation is not fully

aligned as in a natural muscle, and we have stated this explicitly in the text. We would note that the primary aim of this work was to demonstrate functional biohybrid actuators, for which the achieved degree of maturation is sufficient; fuller maturation and better alignment are important goals we identify as future directions.

Controls for MyoHC specificity. We find that MyoHC staining is well-known and generally reported in the biohybrid literature without additional specificity controls (e.g. Chen et al., 2026, Nature Comm.; Guix et al., 2021 Sci. Rob.; Filippi et al., 2023 Adv. Health. Mater.). However, we agree that controls are biologically relevant and therefore we are happy to add the request controls. First, undifferentiated C2C12 cells, which lack mature myofibers, showed no MyoHC signal using the same staining antibodies (Fig. S10), directly confirming that the signal in differentiated samples is specific to mature myofibers. Second, an isotype control (IgG2b–eFluor 450) confirmed the absence of nonspecific binding attributable to the antibody isotype (Fig. S11). We note that the isotype conjugate (eFluor 450) differs from the MyoHC conjugate (eFluor 660); fluorophore-specific background in the 660 channel is instead addressed by the undifferentiated C2C12 control in Fig. S10.

Image quality and number, and the fusion index. To better support the fusion index quantification, we have assembled a new Supplementary Figure S10 presenting 5 representative images of ROIs used for calculating the fusion index. They are representative of the dataset, which included 3 independent samples, 7 images, and 3 ROIs per image. In these images, nuclei incorporated within myotubes can be distinguished from those outside, allowing the basis of the quantification to be assessed directly.

Quantitative data of this kind are conventionally reported as summary graphs accompanied by a few representative images. In the interest of transparency, we have additionally provided this new Supplementary Figure, and the complete set of high-resolution images underlying the fusion index calculation is available from the authors upon request. To avoid disrupting the narrative of the main text, these complementary data are presented in the Supplementary Materials rather than in the main figures. We believe the data are now presented in a complete and convincing manner. Further, as noted previously, the aim of this manuscript is not to demonstrate that our biofabrication method yields the most mature tissues; the fusion index serves as an additional indicator of myofiber formation and organization, which is demonstrated by the functional video data.

Further analyses (sarcomeric organization, mRNA assays). We appreciate this suggestion. Though we did not specifically look for sarcomeres, we would like to note that

the MyoHC+ images do include sarcomeric organization. We have added this into the manuscript main text and into the supplementary as Fig. S10.

*"While the imaging approach was not specifically intended to evaluate sarcomeric organization, features consistent with sarcomeric structure were nevertheless observed. (**Fig. S11**)."* (Lines 289 – 291)

Moreover, assays such as sarcomeric imaging mRNA-based readouts are typically used to infer whether a tissue is likely to be functional. In our case, we assessed functionality directly by demonstrating contractility and motion, which provides a functional validation that these proxy measures are intended to predict. Such detailed assays would be useful in future steps while trying to increase the maturation level of the muscle, and we thank the Reviewer for the suggestion.
