# Supplementary material for: Co-optimized volumetric muscle design for large dynamic deformations in versatile biohybrid robots
Source: Nat Commun. 2026 Sep 11;17:10732. doi: 10.1038/s41467-026-77655-1 (PMC13658790; doi:10.1038/s41467-026-77655-1)
Supplement: Supplementary file 2 [file 41467_2026_77655_MOESM1_ESM.pdf]

Supplementary information for Co-Optimized Volumetric Muscle Design for Large Dynamic Deformations in Versatile Biohybrid Robots

Aiste Balciunaite, Mike Y. Michelis, Miriam Filippi, Pablo Paniagua, Oncay Yasa, Robert K. Katzschmann

Table S1: Values and references used for plotting the contractile tissue volume compared to the maximum contraction deformation of biohybrid robots published in the last two decades.

Reference	Cell Type	Contractile Tissue Volume (mm^3)	Maximum range of motion (mm)
**(a) Feinberg 2007 (*50*)**	cardiac	0.08	4
**(b) Lee 2022 (*4*)**	cardiac	0.27	2.4
**(c) Tetsuka 2022 (*6*)**	cardiac	2.8	2.1
**(d) Tetsuka 2024 (*7*)**	cardiac	2.4	2
**(e) Park 2016 (*5*)**	cardiac	2	1
**(f) Chan 2012 (*51*)**	cardiac	0.14	0.5
**(g) Nawroth 2012 (*8*)**	cardiac	0.13	0.5
**(h) Kim 2007 (*52*)**	cardiac	0.011	0.19
**(i) Xi 2005 (*53*)**	cardiac	0.000055	0.06
**(j) Morita 2024 (*54*)**	cardiac	0.3	0.03
**(k) Williams 2014 (*55*)**	cardiac	0.000022	0.013
**(l) Guix 2021 (*14*)**	skeletal	7.2	0.6
**(m) Cvetkovic 2014 (*9*)**	skeletal	7.1	0.46
**(n) Pagan-Diaz 2018 (*10*)**	skeletal	28.9	0.44
**(o) Kim 2023 (*11*)**	skeletal	29	0.3
**(p) Kinjo 2024 (*12*)**	skeletal	32	0.3
**(q) Morimoto 2018 (*15*)**	skeletal	23	0.16
**(r) Aydin 2019 (*56*)**	skeletal	0	0.02
**(s) Yang 2024 (*13*)**	skeletal	13.3	0.4
**This work**	skeletal	15	6.6

Table S2: Summary of contraction values observed for reported single constructs actuated at 2 Hz

Construct	Frequency	Range of Motion	Percent standard deviation
**Upwards swim**	2 Hz	3.4 mm ± 0.2	6%
**Walker/Gripper**	2 Hz	2.0 mm ± 0.04	2%
**Multi-unit, Unit 1**	2 Hz	0.53 mm ± 0.03	5%
**Multi-unit, Unit 2**	2 Hz	0.6 mm ± 0.01	2%
**Jump**	N/A	6.6 mm	N/A

Table S3: Tukey's post-hoc results for Fig. 4E (height/stroke). ANOVA F-statistic = 18.6, p-value = 5.8e-7

Group	p-value
**1 Hz – 2 Hz**	0.89
**1 Hz – 3 Hz**	8.6e-8
**2 Hz – 3 Hz**	2.7e-8

Table S4: Tukey's post-hoc results for Fig. 4F (contraction).

Group	p-value
**1 Hz – 2 Hz**	2.9e-8
**1 Hz – 3 Hz**	2.9e-2
**2 Hz – 3 Hz**	4.9e-5

Table S5: Values and references used for comparing speed of biohybrid robots

Reference	Cell Type	Propulsion	Characteristic length (mm)	Speed (mm/min)	Body lengths/min
**Xi 2005 (*53*)**	cardiac	swim	0.512	2.28	3.72
**Feinberg 2007 (*50*)**	cardiac	swim	6	20	3.33
**Kim 2007 (*52*)**	cardiac	walk	2	6	3
**Nawroth 2012 (*57*)**	cardiac	swim	6	71.5	11.9
**Chan 2012 (*51*)**	cardiac	walk	7	14.2	2
**Park 2016 (*5*)**	cardiac	swim	14	90	6.42
**Tetsuka 2022 (*6*)**	cardiac	swim	35	34.8	1.0
**Lee 2022 (*4*)**	cardiac	swim	22	660	30
**Tetsuka 2024 (*7*)**	cardiac	swim	30	34.3	1.1
**Cvetkovic 2014 (*9*)**	skeletal	crawl	5.5	9.6	1.5
**Pagan-Diaz 2018 (*10*)**	skeletal	crawl	13.6	30	2.2
**Aydin 2019 (*56*)**	skeletal	swim	3	0.042	0.014
**Guix 2021 (*14*)**	skeletal	swim	12	33.2	2.77
**Kim 2023 (*11*)**	skeletal	walk	14	49.8	3.56
**Balciunaite 2023 (*31*)**	skeletal	swim	7.9	122	15.4
**Kinjo 2024 (*12*)**	skeletal	walk	16	5.4	0.34
**Yang 2024 (*13*)**	skeletal	walk	18.6	143	7.6

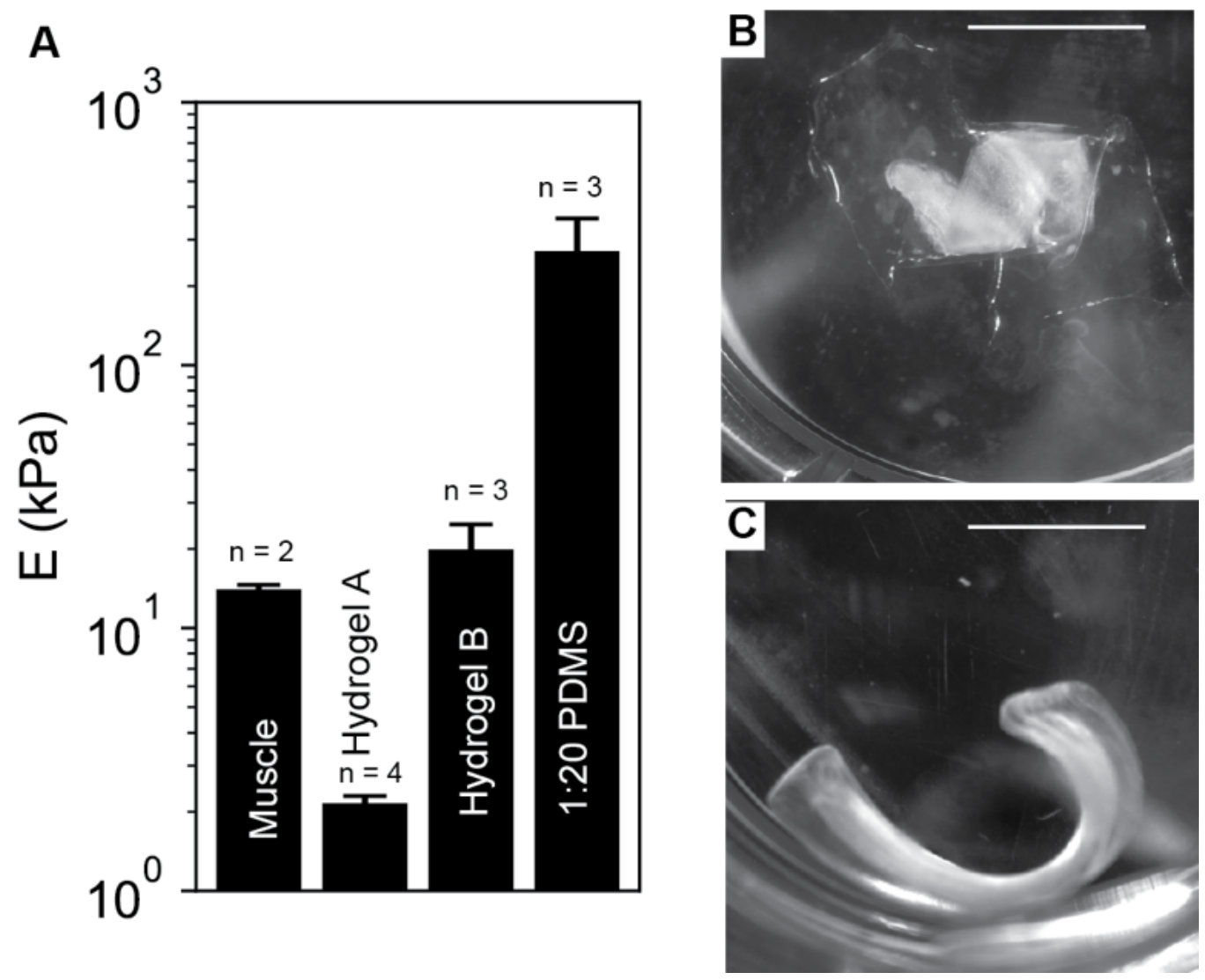

Figure S1: Effect of scaffold stiffness of bilayered actuator maturation. **(A)** Young's modulus of muscle and two different hydrogels and 1:20 PDMS. Hydrogel A: 20% PEGDA M.W. 575 + 5% GelMA. Hydrogel B: 15% PEGDA M.W. 700 + 5% GelMA. **(B)** Bilayered bioactuator at the end of maturation on softer Hydrogel A. **(C)** Bilayered bioactuator at the end of maturation on optimal Hydrogel B. 1:20 PDMS included for comparison, as this is a common material for thin film formation.

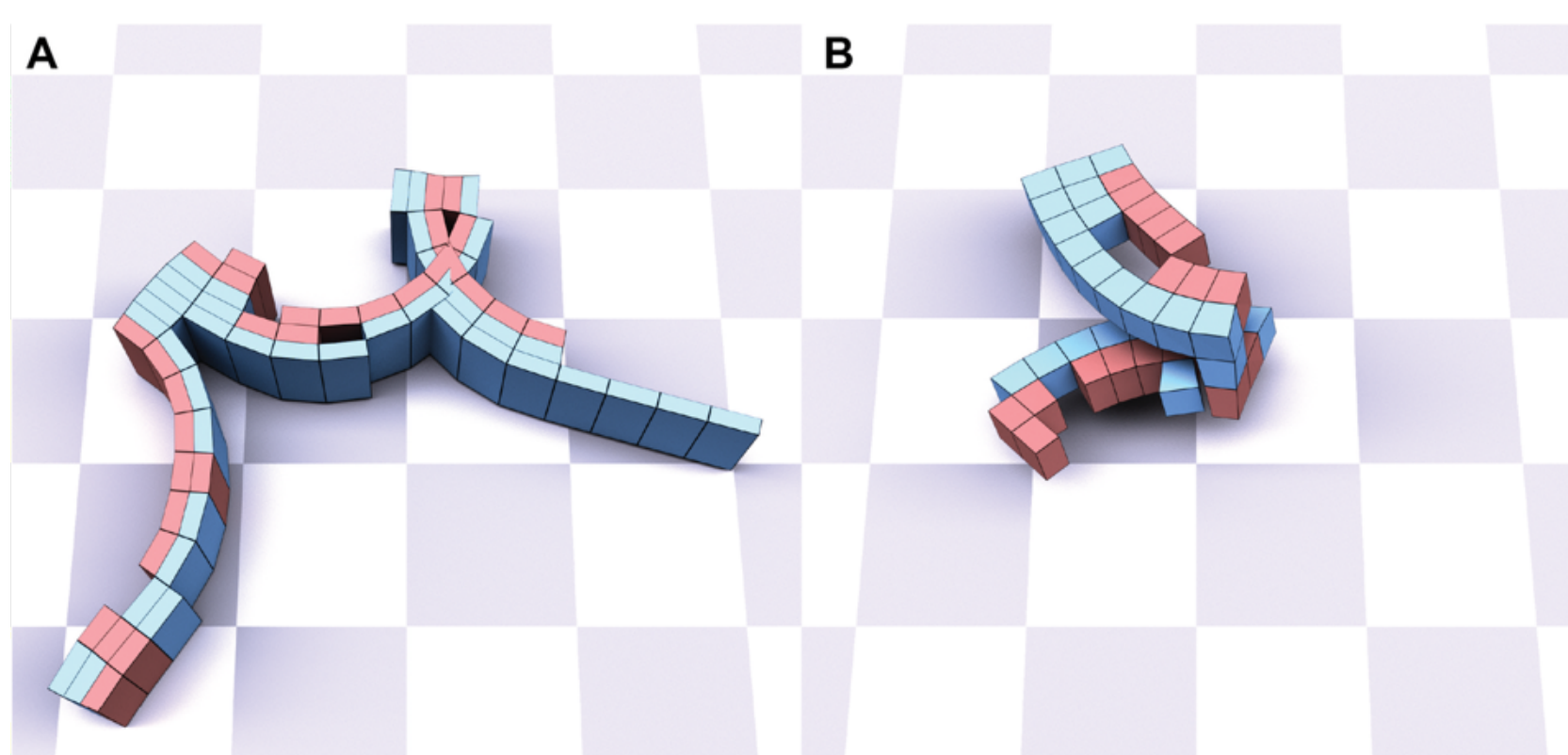

Figure S2: Unconstrained topology optimization for muscle allocation. Every cell is marked as either empty, passive (blue scaffold), or active (red muscle). **(A)** A constant depth (z-axis) search over a 10x10x1 grid where self-collision is allowed. We see a natural formation of bilayers with empty space between the active-passive layer combination. For 10 layers along the y-axis, the optimal amount of bilayers is 3, which the genetic algorithm finds. **(B)** For a 3D search over a 9x3x3 voxel grid, we find the algorithm converging to two distinct bilayers. These experiments prove that heuristically the designs try to evolve into structures with one layer of active and one layer of passive cells.

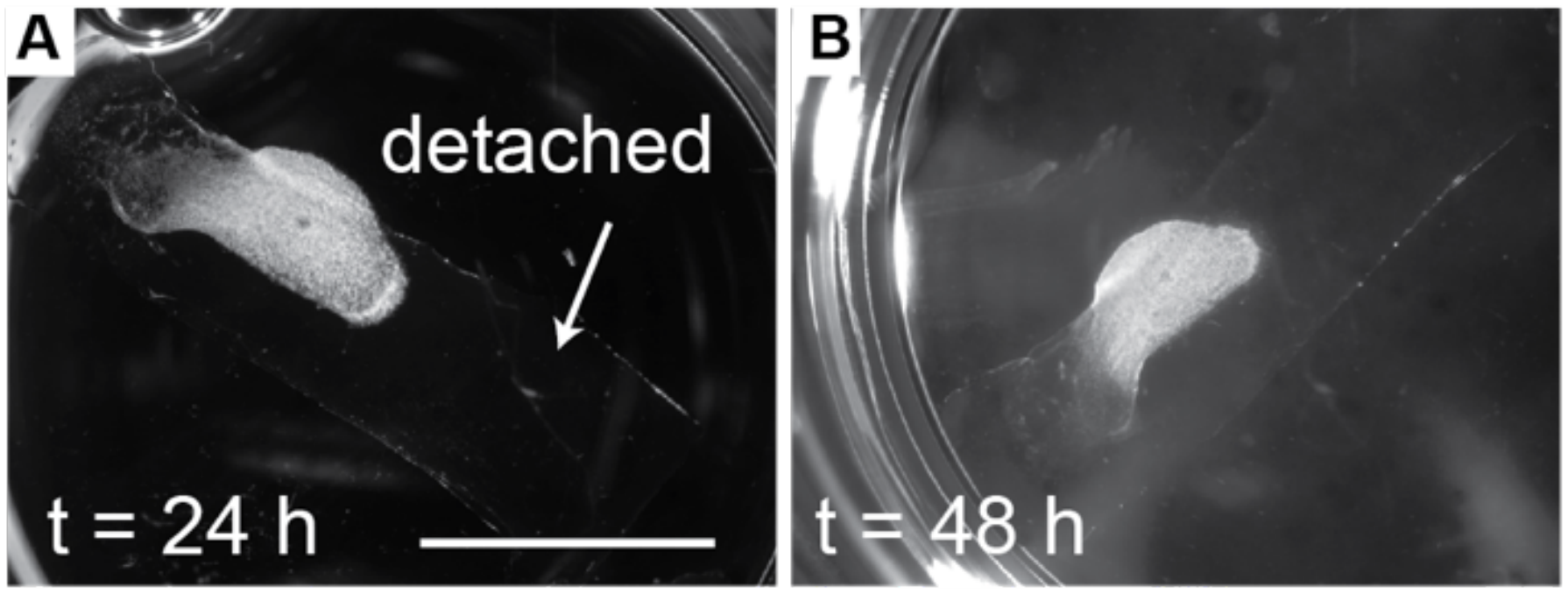

Figure S3: Stereomicroscope image of muscle layer detaching from a scaffold at **(A)** 24 hours and **(B)** 48 hours post-seeding. The detached section is indicated with an arrow. The scale bar is 5 mm.

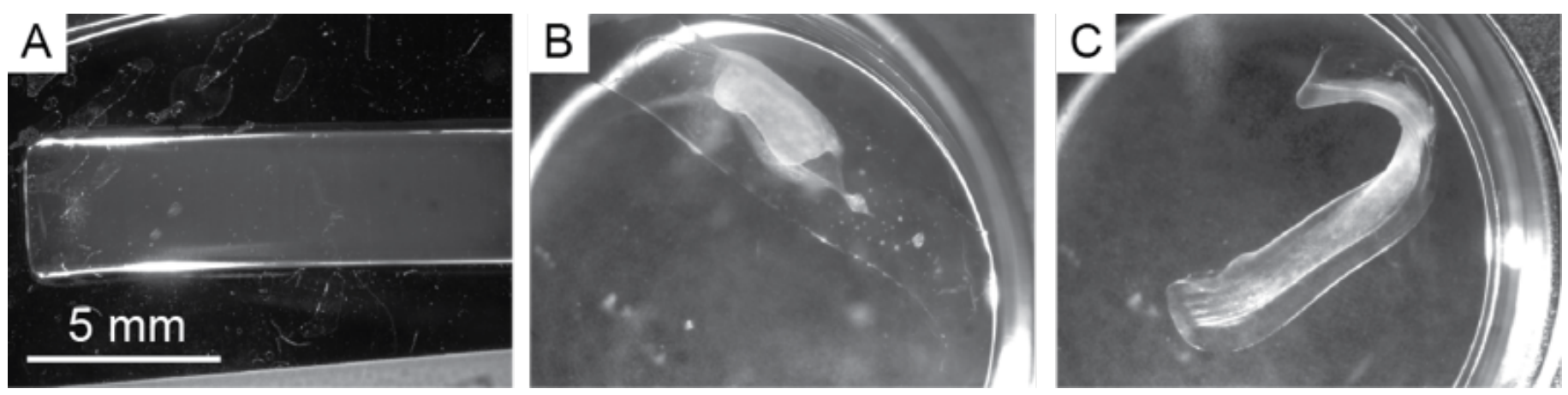

Figure S4: Comparison of muscle layer adhesion to a microgrooved and not grooved scaffold. **(A)** Image of the scaffold without grooves; all other dimensions are the same as the micro-grooved scaffold. **(B)** Image of the bilayered actuator after 4 days on an ungrooved scaffold, showing that the muscle tissue unadhered from the sides and shrunk inwards. **(C)** Image of the bilayered actuator after 4 days on a microgrooved scaffold, showing that the tissue is adhering along the length of the actuator. Scale is equivalent for all images.

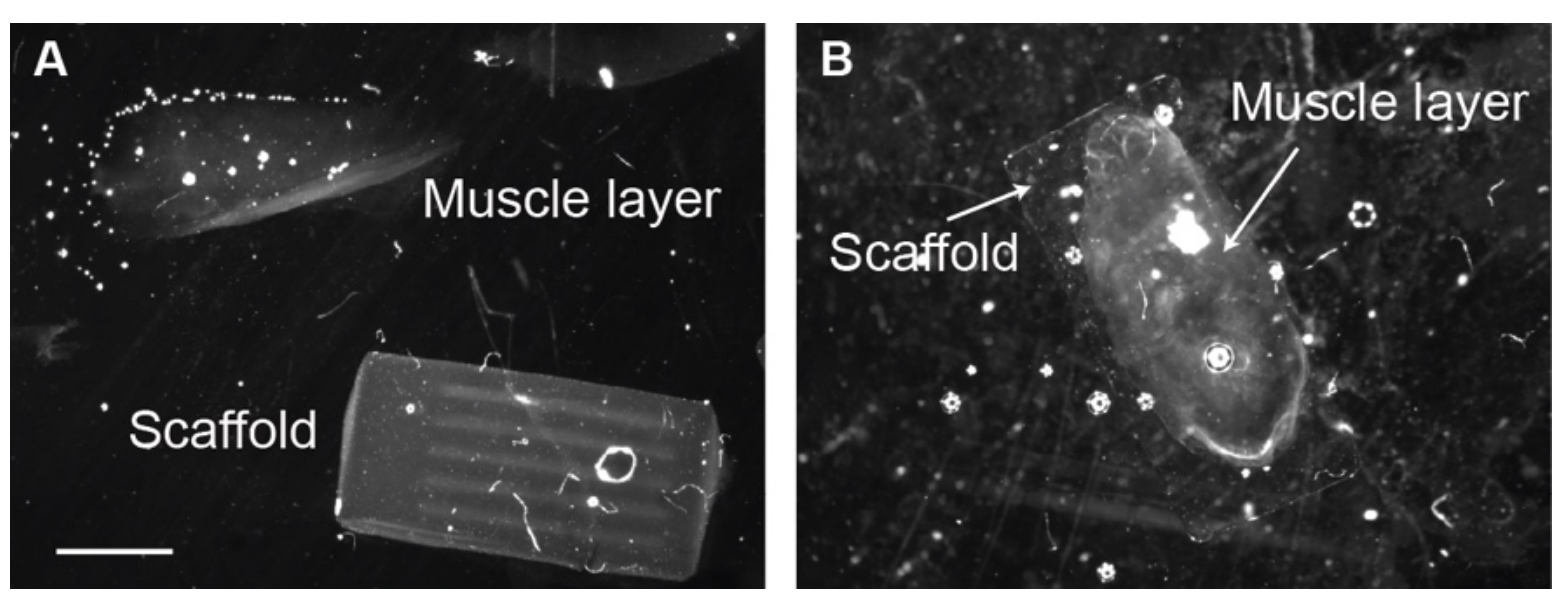

Figure S5: Examples of a **(A)** wet scaffold that unadhered after adding media, and a **(B)** dry scaffold that stayed adhered. Scale bar 5 mm.

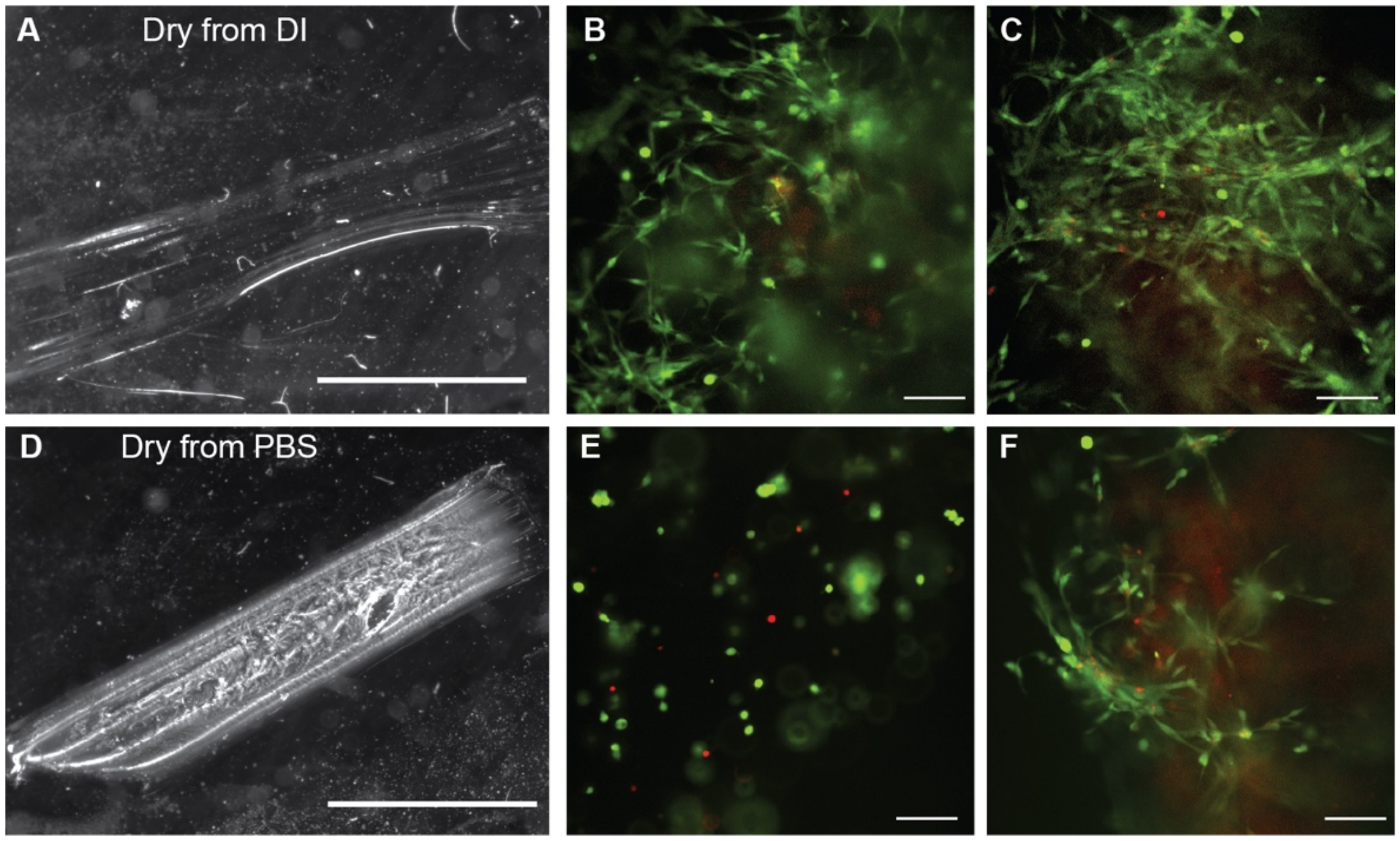

Figure S6: Optimization of protocol for myoblast viability using live/dead staining. **(A)** Scaffold post-processed and dried in DI water. **(B, C)** Confocal microscopy images of live/dead staining of C2C12 seeded in muscle hydrogel on top of DI-dried scaffold after 24 hours. **(C)** Scaffold post-processed and dried in PBS. **(E, F)** Confocal microscopy images of live/dead staining of C2C12 seeded in muscle hydrogel on top of PBS-dried scaffold after 24 hours. Green cells are alive, and red cells are dead. Spreading of cells into an elongated shape indicates good cell health. Scale bars are 5 mm (A, D) and 100 µm (B, C, E, F).

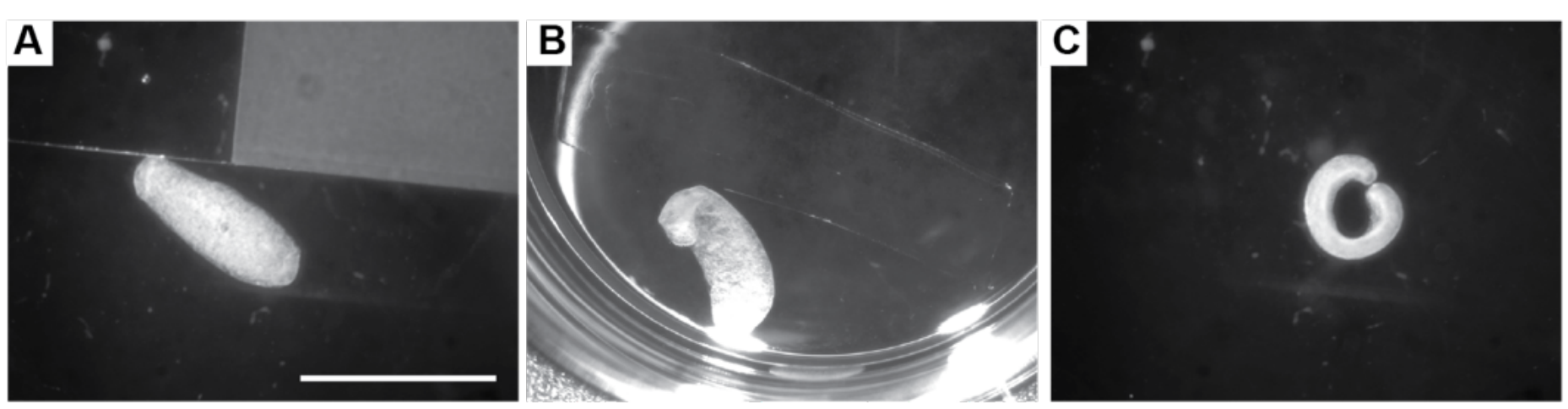

Figure S7: Unadhered muscle tissue layer. **(A-C)** Muscle tissue layer unadhered after 2 days of growth media. All muscle tissue started with a length of 15 mm. The muscle tissue shrinks when it does not have the scaffold to provide a restoring force.

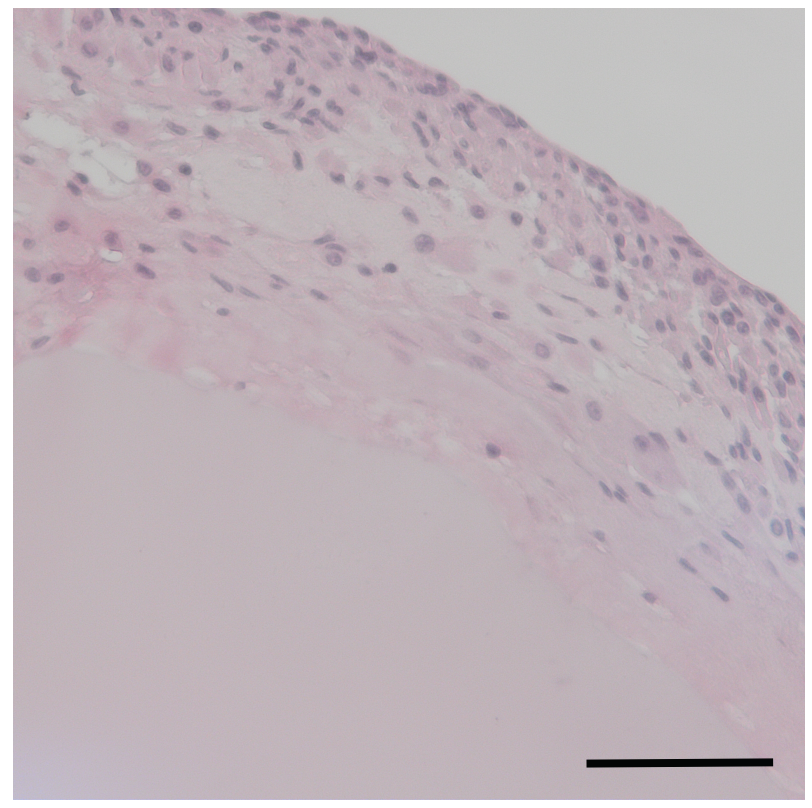

Figure S8: Example of scaffold and muscle tissue separation in a histological cross-section. Scale bar 100 µm.

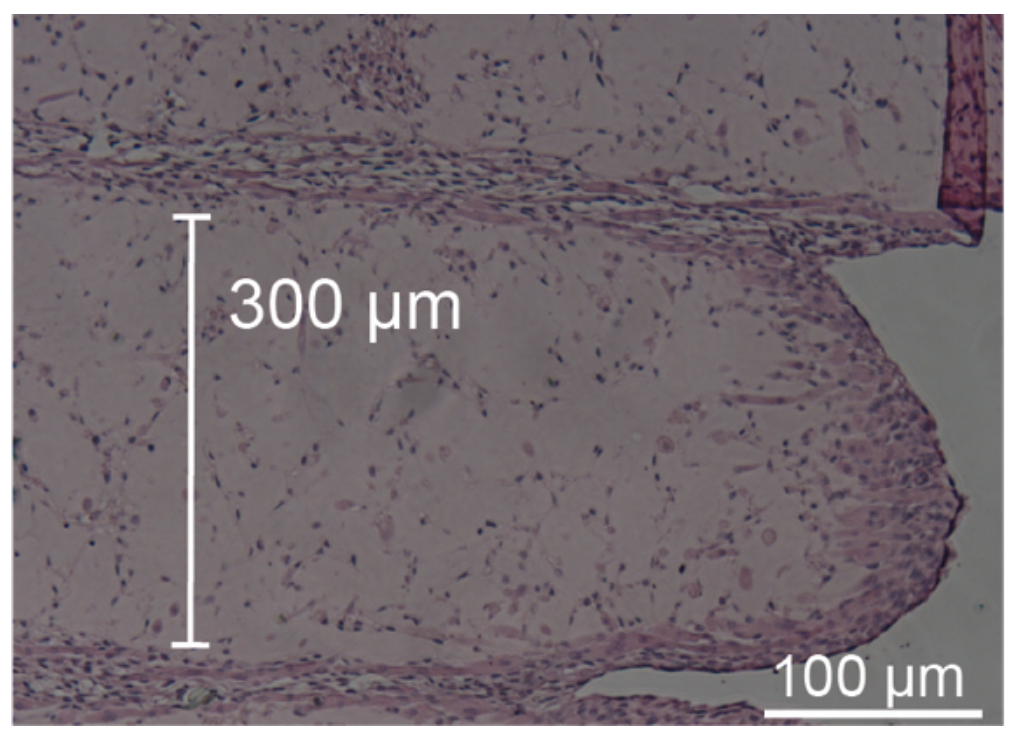

Figure S9: Histological cross-section showing the preferential myofiber alignment at groove edges (spacing between stripes of myofibers is 300 µm).

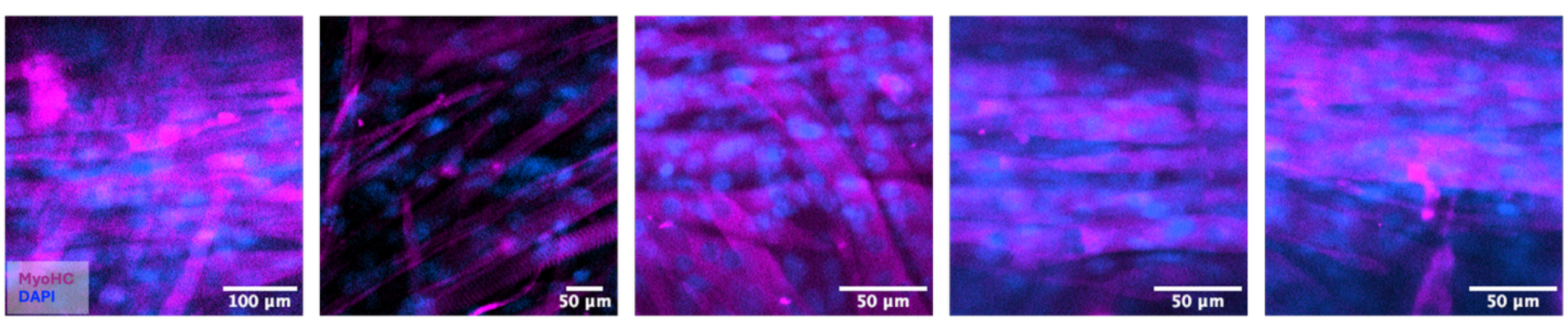

Figure S10: Representative ROIs used to calculate fusion index. Original images are available from the authors upon request.

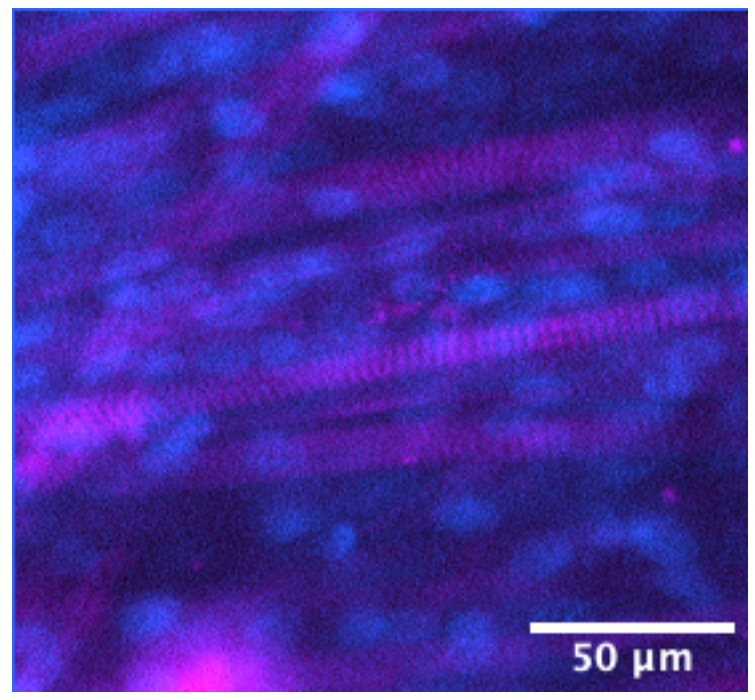

Figure S11: Close-up of the MyoHC+ staining image in Fig. 4, showing sarcomeric organization.

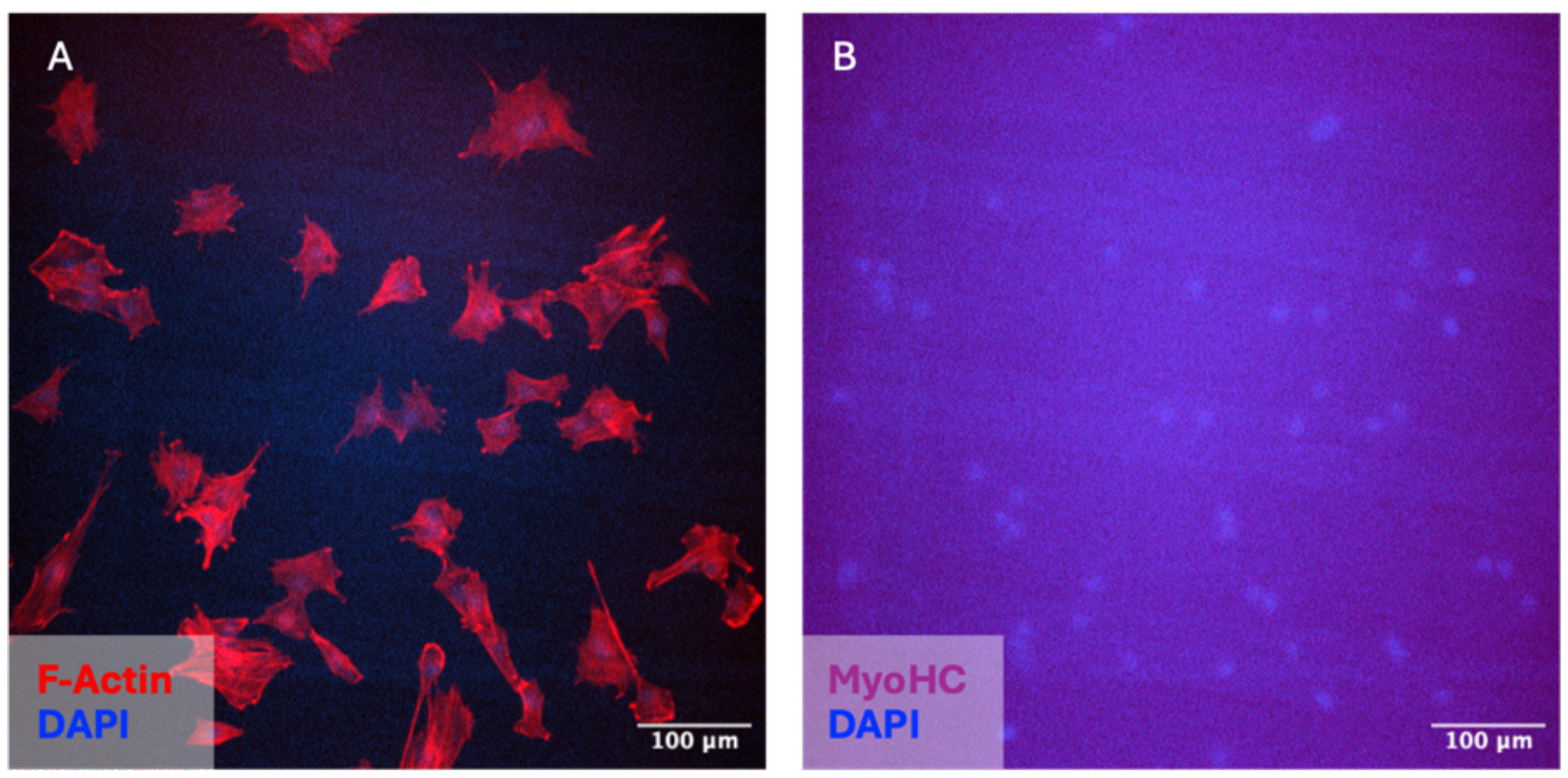

Figure S12: Control for the specificity of MyoHC staining for mature myofibers, conducted on 2D undifferentiated C2C12 cells using the same MyoHC-eFluor 660 conjugate. (A) F-Actin and DAPI staining of C2C12 cells. (B) Same region stained for MyoHC. As seen in (B), there is no MyoHC signal on undifferentiated C2C12 cells.

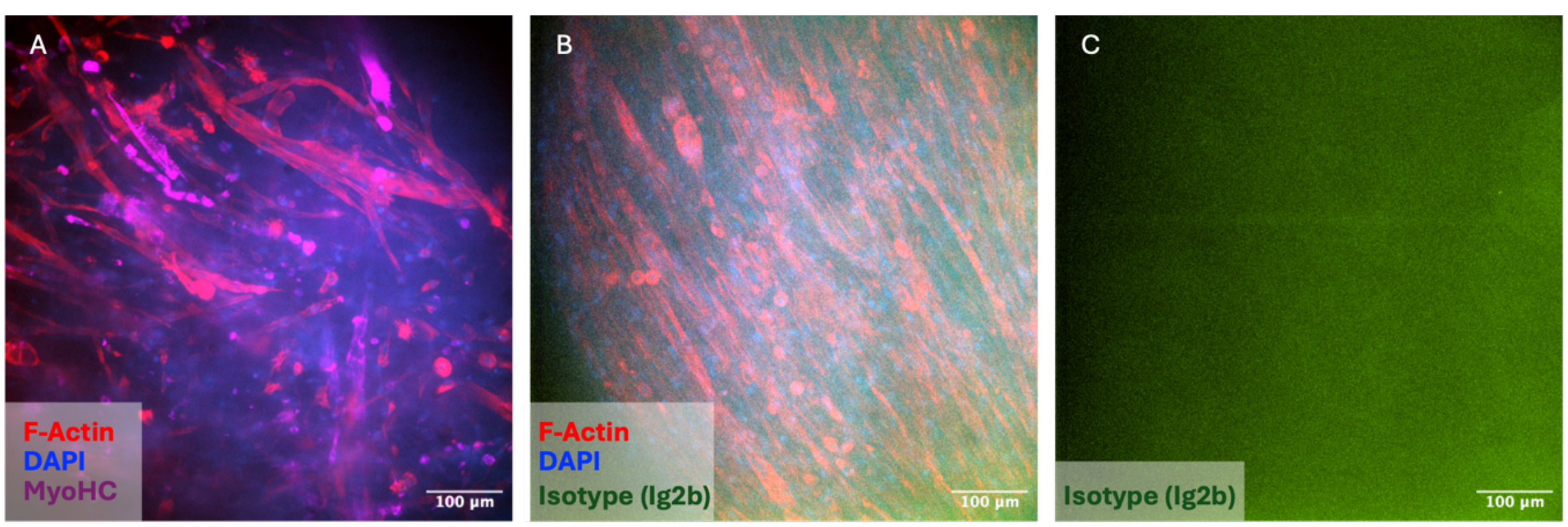

Figure S13: Control for nonspecific MyoHC staining on differentiated volumetric bioactuators. (A) F-Actin (555 nm), DAPI (395 nm), and MyoHC (640 nm) staining of a differentiated muscle sample. (B) F-Actin (555 nm), DAPI (395 nm), and isotype control (IgG2b, 405 nm) of a differentiated sample. (C) Single-color isotype control (IgG2b, 405 nm) showing the lack of staining of any structures.

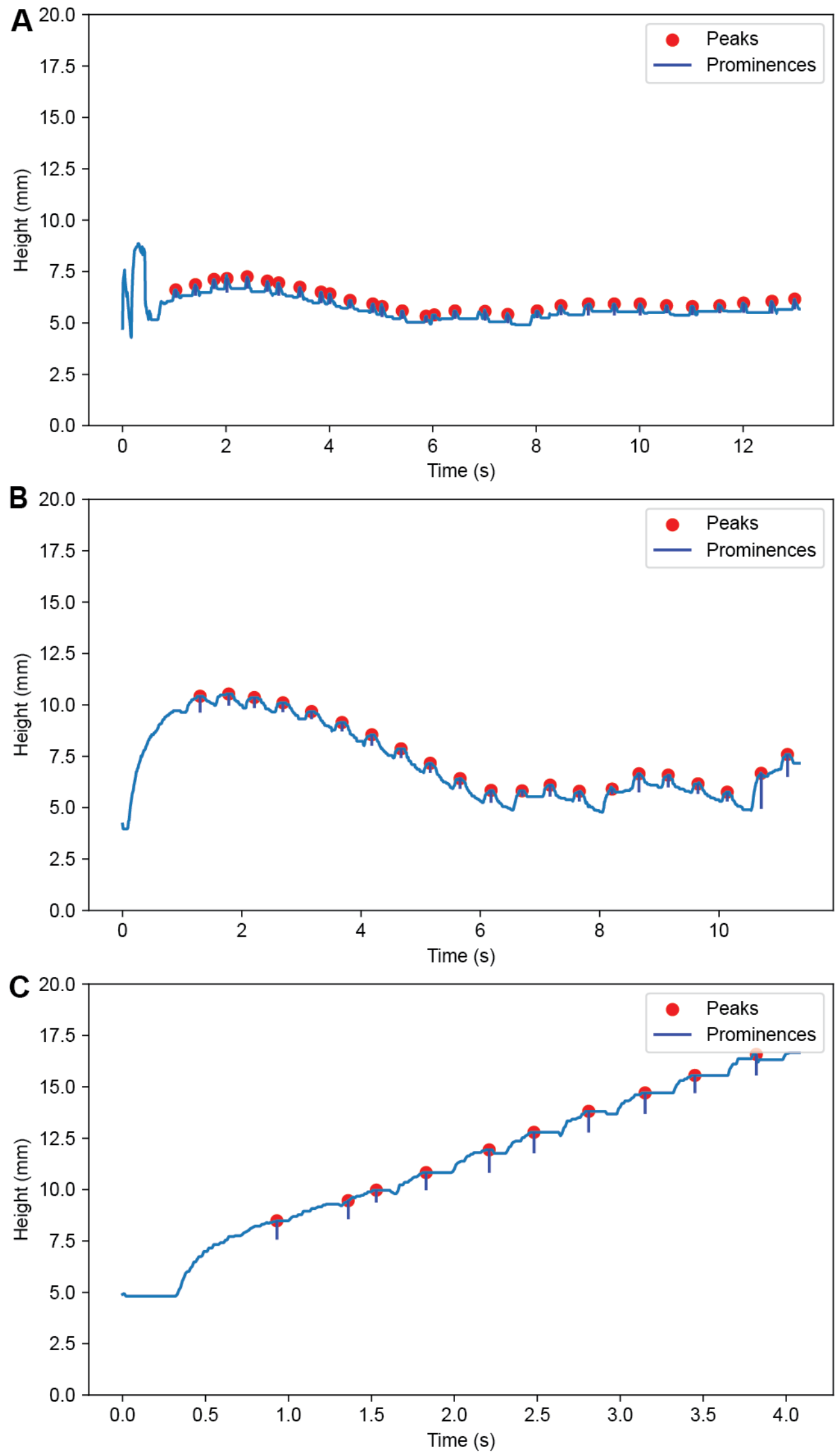

Figure S14: Swimming biohybrid robot tracks with peaks and peak heights indicated. The tracks correspond to height gain over time for **(A)** 1 Hz actuation, **(B)** 2 Hz actuation, and **(C)** 3 Hz actuation.

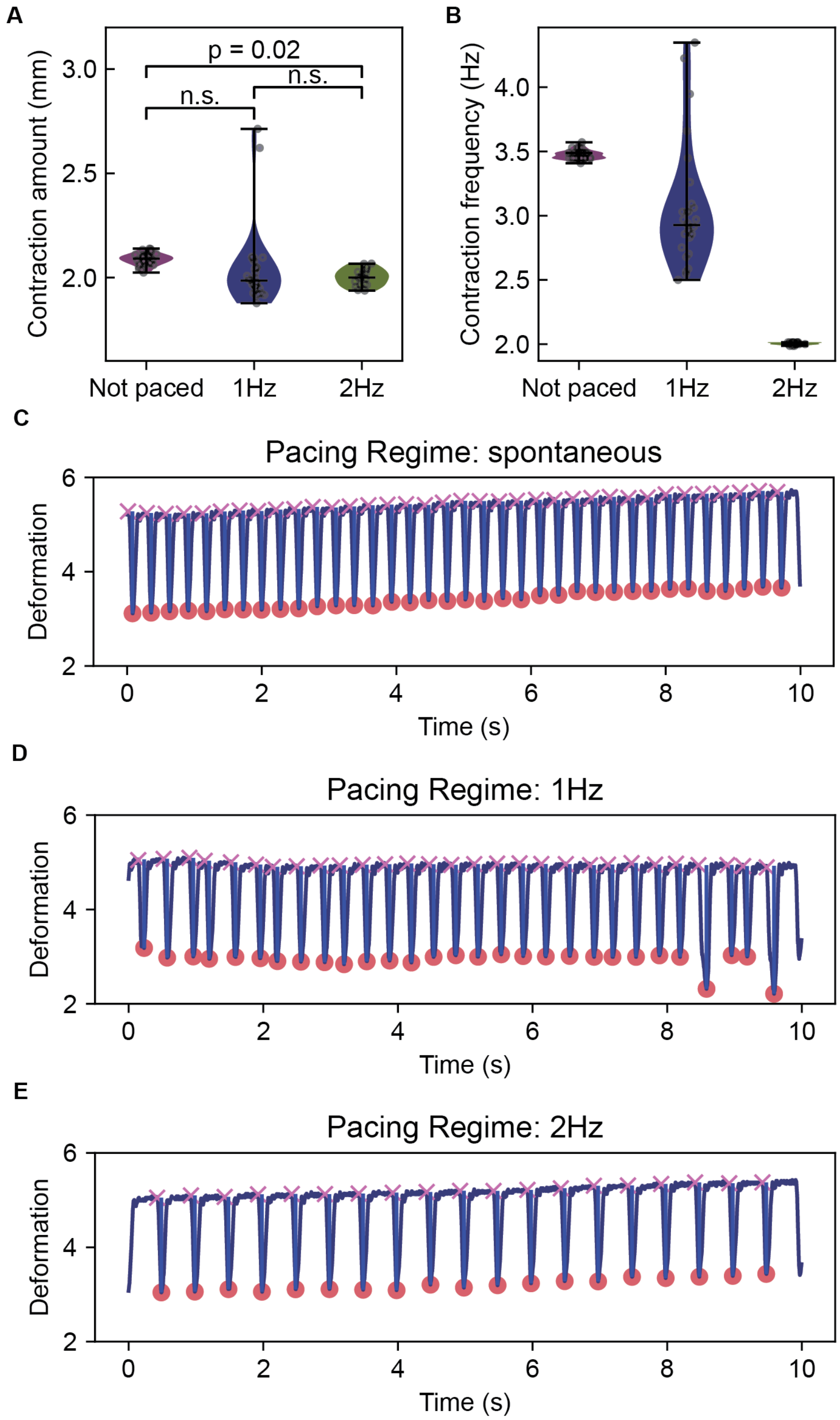

Figure S15: Muscle dynamics analysis of bilayered bioactuator used for gripping and walking. **(A)** Contraction amount of the bioactuator with spontaneous contractions and paced at 1 Hz and 2 Hz. (B) Contraction frequency of the bioactuator when not paced and at 1 Hz and 2 Hz pacing. Note that at 1 Hz, the bioactuator is still contracting spontaneously and therefore the pacing frequency is varied and not consistent at 1 Hz. (C – E) Tracks of the bioactuator opening and closing used for the analysis in panels A and B. Peaks (closed state) are indicated with a red dot and the base of the peak (open state) is indicated with an 'x'.

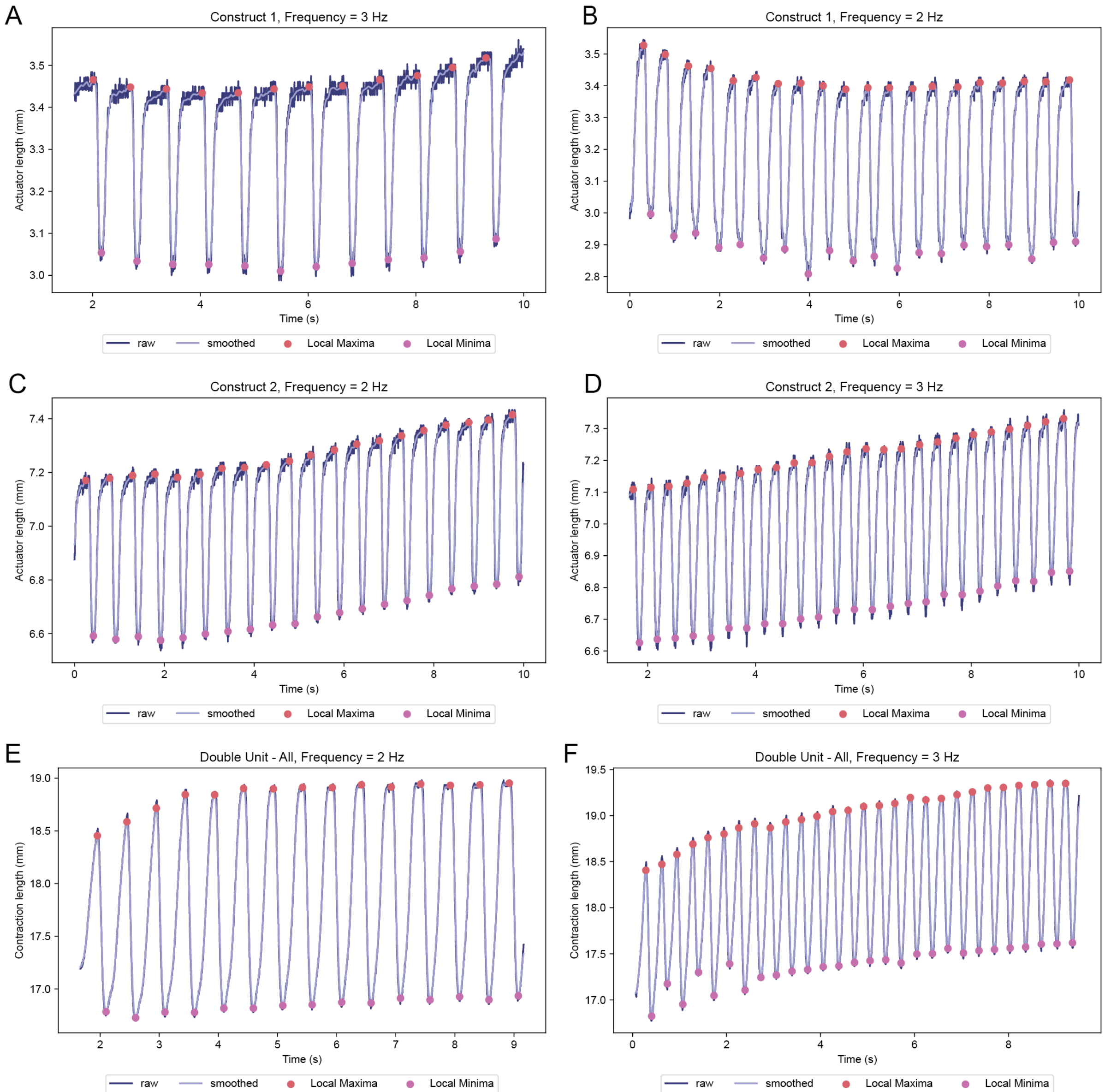

z

Figure S16: Single and multi-unit contraction dynamics analysis. Absolute actuator length is shown on the y-axis over time on the x-axis. Data was smoothed (dark line) to find maxima and minima of contraction peaks (dots). **(A)** Construct 1 paced at 2 Hz. **(B)** Construct 1 paced at 3 Hz. **(C)** Construct 2 paced at 2 Hz. **(D)** Construct 2 paced at 3 Hz. **(E)** Double unit paced at 2 Hz. **(F)** Double unit paced at 3 Hz.

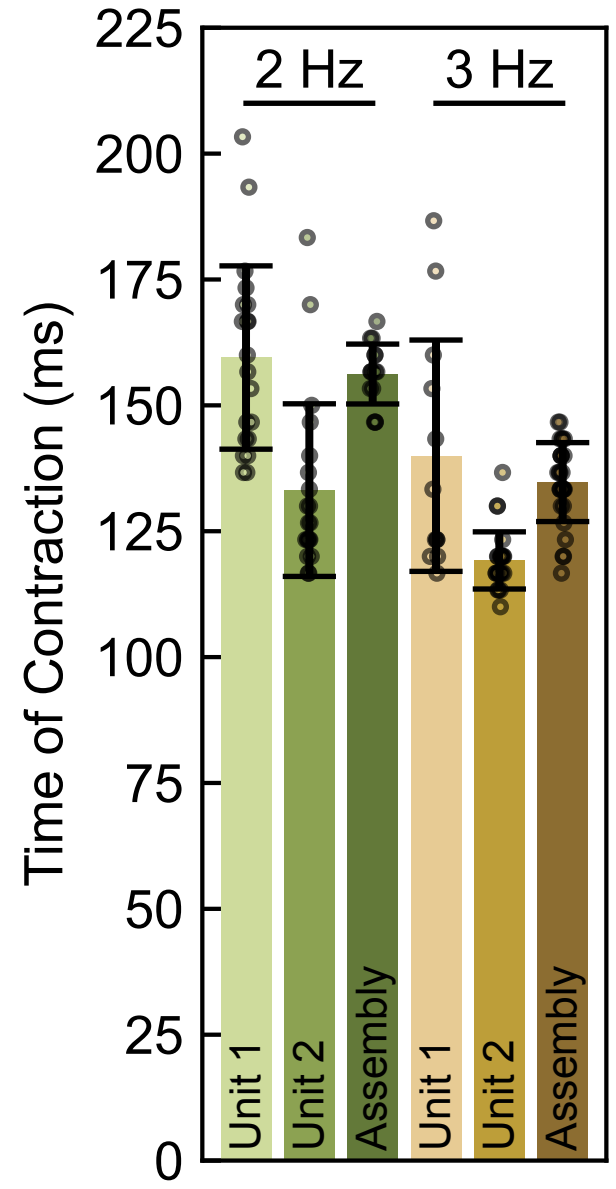

Figure S17: Single and multi-unit time of contraction at 2Hz and 3Hz.
