# Supplementary material for: Co-optimized volumetric muscle design for large dynamic deformations in versatile biohybrid robots
Source: Nat Commun. 2026 Sep 11;17:10732. doi: 10.1038/s41467-026-77655-1 (PMC13658790; doi:10.1038/s41467-026-77655-1)
Supplement: Supplementary file 3 [file 41467_2026_77655_MOESM2_ESM.pdf]

**<u>Description of Additional Supplementary Files</u>**

Supplementary Movie 1: Biohybrid jumping robot (5 V/cm)

Supplementary Movie 2: Biohybrid swimming robot actuated at 1 Hz (3.3 V/cm)

Supplementary Movie 3: Biohybrid swimming robot actuated at 2 Hz (3.3 V/cm)

Supplementary Movie 4: Biohybrid swimming robot actuated at 3 Hz (3.3 V/cm)

Supplementary Movie 5: Walking bilayered bioactuator un paced and paced contraction at 1 Hz and 2 Hz (4 V/cm)

Supplementary Movie 6: Biohybrid walker travelling around a pillar

Supplementary Movie 7: Biohybrid walker turning in front of pillar

Supplementary Movie 8: Biohybrid gripper actuated by a bilayered bioactuator (4 V/cm)

Supplementary Movie 9: Single bilayered bioactuator paced at 2 and 3 Hz (3 V/cm)

Supplementary Movie 10: Double unit bilayered bioactuator paced at 2 and 3 Hz (3 V/cm)

Supplementary Movie 11: Multi-leg bilayered bioactuator paced at 2Hz.

Supplementary Movie 12: Multi-leg bilayered bioactuator paced at 3Hz.
